# Marine Indole Alkaloids

**DOI:** 10.3390/md13084814

**Published:** 2015-08-06

**Authors:** Natalie Netz, Till Opatz

**Affiliations:** Institute of Organic Chemistry, Johannes Gutenberg-University Mainz, Duesbergweg 10-14, 55128 Mainz, Germany; E-Mail: netz@uni-mainz.de

**Keywords:** indoles, alkaloids, marine natural products, bisindoles, nitrogen heterocycles, carbolines, prenylated indoles, diketopiperazines

## Abstract

Marine indole alkaloids comprise a large and steadily growing group of secondary metabolites. Their diverse biological activities make many compounds of this class attractive starting points for pharmaceutical development. Several marine-derived indoles were found to possess cytotoxic, antineoplastic, antibacterial and antimicrobial activities, in addition to the action on human enzymes and receptors. The newly isolated indole alkaloids of marine origin since the last comprehensive review in 2003 are reported, and biological aspects will be discussed.

## 1. Introduction

Alkaloids represent a large and highly structurally diverse group of secondary metabolites. The presence of nitrogen in their molecular architecture confers biological activity to an exceptionally large fraction of this compound class. Therefore, it comes as no surprise that mammals—including man—have acquired the ability to detect the potentially toxic alkaloids by their bitter taste.

As the origin of life on Earth presumably was the early hydrosphere, the evolution of aquatic life forms has the longest history and a connection may be seen in the enormous chemical complexity of natural marine products.

This review focuses on marine indole alkaloids, discovered since the last comprehensive report by Aygün and Pindur in 2003 [1]. In addition to structures and occurrence, known biological activities of marine indole alkaloids will be discussed. We will make use of Pelletier’s general definition of 1983, according to which alkaloids are “cyclic organic compounds containing nitrogen in a negative oxidation state which are of limited distribution among living organisms” [2]. As an additional demarcation against the world of peptides, polypeptidic structures and macrocyclic peptides derived from tryptophan, such as terpeptins [3] and related structures [4], milnamides [5,6], diazonamides [7], lucentamycin B [8], pipestelides [9], kahalalides [10,11,12], jaspamides [13], jasplakinolides [14], *etc.*, will not be discussed here. Indole alkaloids which were isolated from genetically engineered marine derived organisms, from organisms with an artificially altered gene regulation, or which were obtained through genetic engineering of terrestrial organisms using genes of marine organisms will not be discussed in this review [15,16,17,18,19,20,21].

The indole nucleus is one of the most important ring systems for pharmaceutical development and has been termed a “privileged structure” in this respect [22]. It is frequently associated with the action on G-protein coupled receptors, in particular with the modulation of neuronal signal transmission through receptors for serotonin (5-hydroxytryptamine, 5-HT). A large variety of effects on other molecular targets have also been reported, including glycine-gated chloride channel receptors, human protein tyrosine phosphatase-1B, the CXCR4 (C-X-C chemokine receptor type 4) chemokine receptor, Na^+^/K^+^-ATPase, nitric oxide synthase, β-secretase, protein kinase C-α, butyrylcholinesterase, and acetylcholinesterase. Furthermore, cytotoxic, antineoplastic, antibacterial, antifungal, antiinsecticidal, and antiplasmodial activities have been detected.

Apart from its capacity to act as a hydrogen bond donor through a free NH function, the high π-electron density and the high HOMO (highest occupied molecular orbital) energy of the planar indole skeleton permit interactions with nucleobases—in particular protonated ones—as well as target proteins, some of which exhibit a high binding specificity for the indole nucleus. The electronic properties together with the relatively low resonance energy of the five-membered ring also determine the chemical behavior of indoles and many of their derivatives. Thus, electrophilic substitutions or oxidative transformations, partly under loss of the aromatic stabilization, are paramount for this compound class, which is also reflected by the structures of many of the compounds discussed in this review.

Regarding related work, an overview of the biosynthesis of indole alkaloids from fungal origin has been published by Xu *et al.* [23]. Alkaloids from marine algae are discussed by Güven *et al.* [24] and halogenated indole alkaloids from marine invertebrates have been reviewed by Pauletti *et al.* [25].

## 2. Marine Indole Alkaloids

### 2.1. Simple Indole Alkaloids

The simple indole alkaloids are mostly derived from tryptophan or its direct precursor indole, which itself is formed from chorismate through anthranilate and indole*-*3-glycerol-phosphate in microorganisms and plants. As the ultimate step of the tryptophan biosynthesis is reversible, free indole can also be formed in this catabolic process [26]. Electrophilic substitutions with iodine and especially bromine are frequently encountered in this and other subclasses presented here while the even more common prenylated indoles, with and without halogen substituents, will be discussed in a separate section.

**Figure 1:**
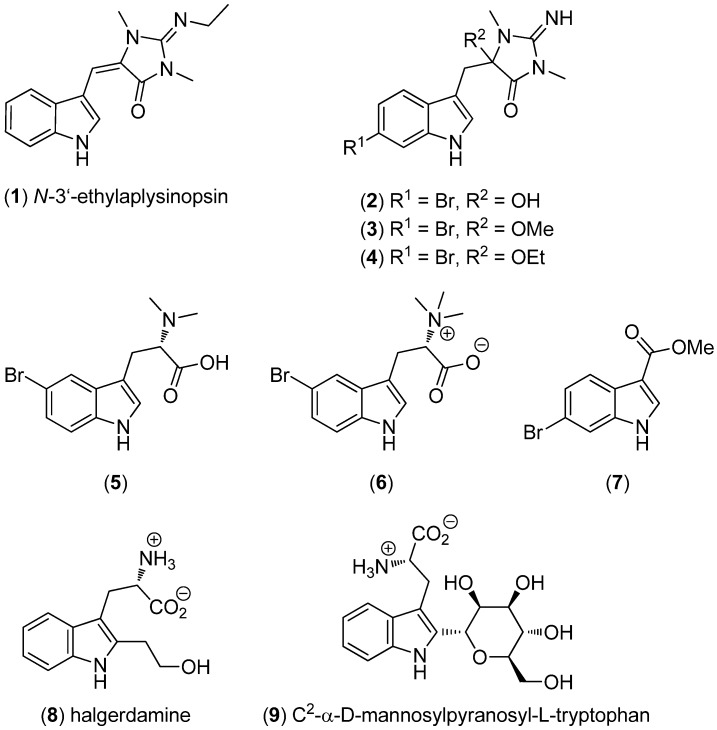
Simple indole derivatives.

*N-*3′-Ethylaplysinopsin (**1**) was obtained from the Jamaican sponge *Smenospongia aurea* and exhibited a high affinity for 5-HT_2A_ and 5-HT_2C_ receptors (Figure 1) [27]. 6-Bromo-1′-hydroxy-1′,8-dihydroaplysinopsin (**2**), 6-bromo-1′-methoxy-1′,8-dihydroaplysinopsin (**3**), 6-bromo-1′-ethoxy-1′,8-dihydroaplysinopsin (**4**), (−)-5-bromo-*N*,*N*-dimethyltryptophan (**5**), (+)-5-bromohypaphorine (**6**) and 6-bromo-1*H*-indole-3-carboxylic acid methyl ester (**7**) were obtained by investigation of an NCI-DTP (National Cancer Institute*,* Developmental Therapeutics Program) collection of *Thorectandra* sp. and a UCSC (University of California, Santa Cruz) collection of *Smenospongia* sp. They show weak inhibitory activity towards *Staphylococcus epidermidis* [28]. Halgerdamine (**8**) and C^2^-α-d-mannosylpyranosyl-l-tryptophan (**9**) were isolated from the nudibranch mollusc *Halgerda aurantiomaculata*. C^2^-α-d-Mannosylpyranosyl-l-tryptophan (**9**) was isolated from a mollusc for the first time [29].

**Figure 2:**
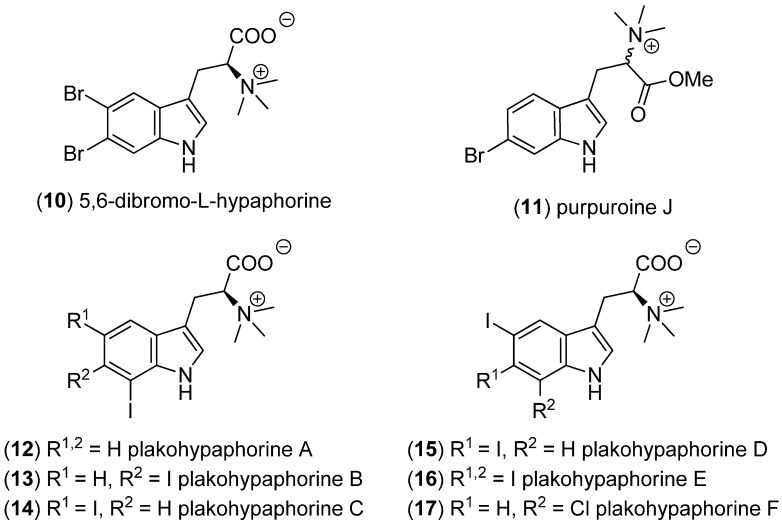
5,6-Dibromo-l-hypaphorine, purpuroine J and plakohypaphorines A–F.

5,6-Dibromo-l-hypaphorine (**10**) has been isolated from the South Pacific marine sponge *Hyrtios* sp. and displays weak inhibitory effects against bee venom phospholipase A_2_ (IC_50_—inhibitory concentration to effect a 50% inhibition—0.2 mM) and a significant antioxidant activity (Figure 2) [30]. Purpuroines A–J were isolated from the marine sponge *Iotrochota purpurea,* purpuroine J (6-bromohypaphorine methylate, **11**) being the only indole-derived congener [31]. Iodinated plakohypaphorines A–F (**12**–**17**) have been isolated from the Caribbean sponge *Plakortis simplex*. Diiodinated species plakohypaphorines B (**13**), C (**14**) and D (**15**) exhibited significant antihistaminic activity [32,33].

**Figure 3:**
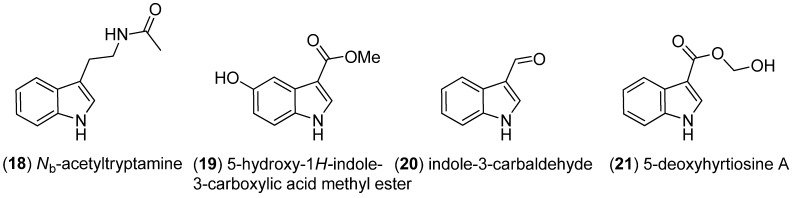
Simple indole derivatives.

Examination of the organic extract of the broth of an unidentified algicolous fungus collected from the surface of the marine red alga *Gracilaria verrucosa* led to the isolation of *N*_b_-acetyltryptamine (**18**) (Figure 3) [34]. Analysis of the Red Sea marine sponge *Hyrtios erectus* led to the isolation of 5-hydroxy-1*H*-indole-3-carboxylic acid methyl ester (**19**), indole-3-carbaldehyde (**20**) and 5-deoxyhyrtiosine A (**21**) [35,36].

**Figure 4:**
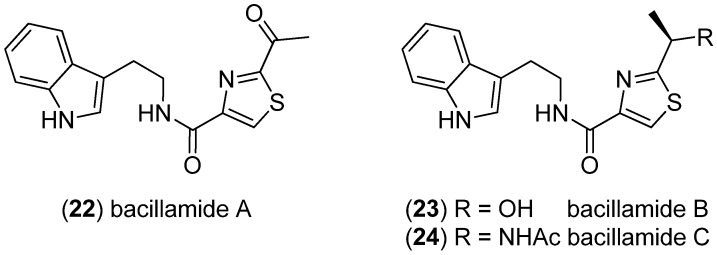
Bacillamides A–C.

Bacillamide A (**22**) was obtained from the marine bacterium *Bacillus* sp. SY-1 and displayed algicidal activity against *Cochlodinium polykrikoides* (Figure 4) [37]. Bacillamides A–C (**22**–**24**) were found in a *Bacillus endophyticus* isolate SP31 [38].

**Figure 5:**
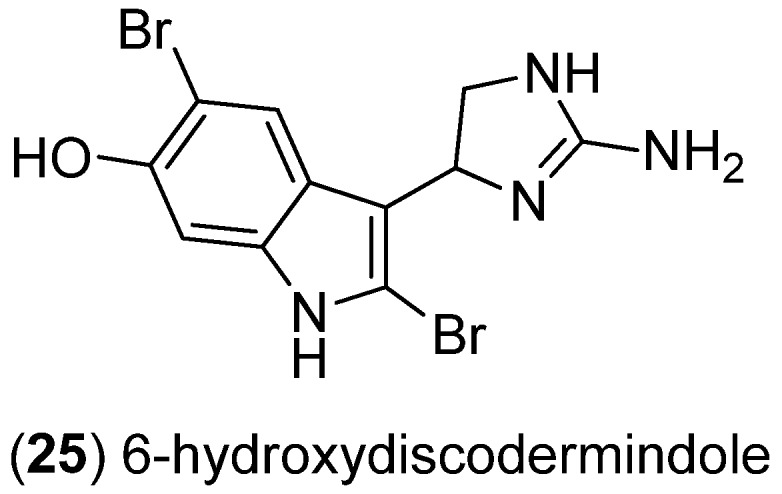
6-Hydroxydiscodermindole.

6-Hydroxydiscodermindole (**25**) was obtained from the deep-water sponge *Discodermia polydiscus* and showed weak cytotoxic effects towards the murine leukemia cell line P388 (Figure 5) [39].

**Figure 6:**
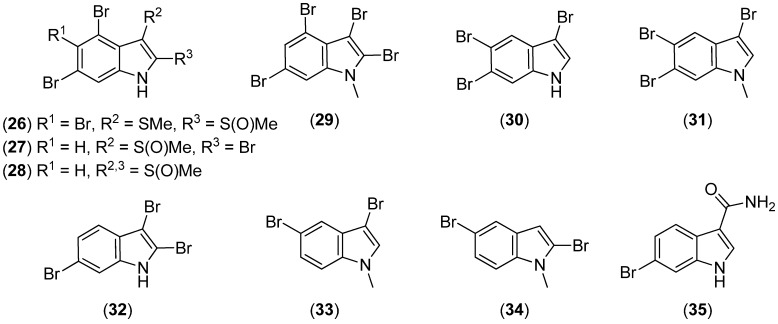
Simple brominated indole derivatives.

2-Methylsulfinyl-3-methylthio-4,5,6-tribromoindole (**26**), 3-methylsulfinyl-2,4,6-tribromoindole (**27**) and 4,6-dibromo-2,3-di(methylsulfinyl)indole (**28**) were isolated from the Formosan red alga *Laurencia brongniartii* (Figure 6) [40]. 2,3,4,6-Tetrabromo-1-methyl-1*H*-indole (**29**) was isolated from the marine red alga *Laurencia decumbens* [41]. 3,5,6-Tribromo-1*H*-indole (**30**), 3,5,6-tribromo-1-methyl-1*H*-indole (**31**), 2,3,6-tribromo-1*H*-indole (**32**), 3,5-dibromo-1-methyl-1*H*-indole (**33**) and 2,5-dibromo-1-methyl-1*H*-indole (**34**) have been isolated from the marine red alga *Laurencia similis* [42,43,44]. 6-Bromo-1*H*-indole-3-carboxamide (**35**) was obtained from the marine sponge *Mycale fibrexilis* [45].

**Figure 7:**
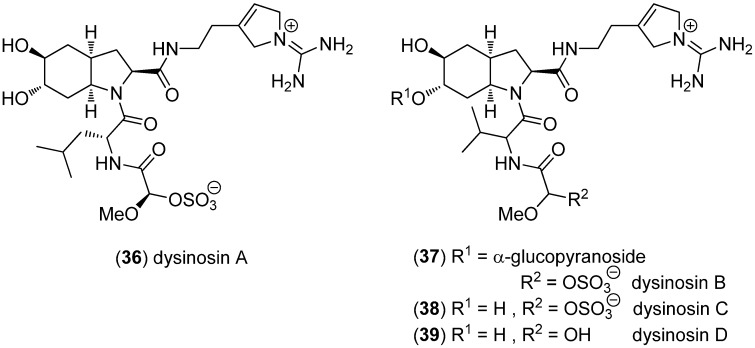
Dysinosins A–D.

Dysinosins B–D (**37**–**39**) were isolated from the Australian sponge *Lamellodysidea chlorea* [46], while dysinosin A (**36**) [47] was obtained from an Australian sponge of the family Dysideidae (Figure 7). Dysinosins A–D exhibit inhibitory activity towards factor VIIa (*K*_i_—dissociation constant—values 0.108, 0.090, 0.124 and 1.320 μM, respectively) and thrombin (*K*_i_ 0.452, 0.170, 0.550 and >5.1 μM, respectively), with desulfated dysinosin D (**39**) being ten times less active [46,47].

**Figure 8:**
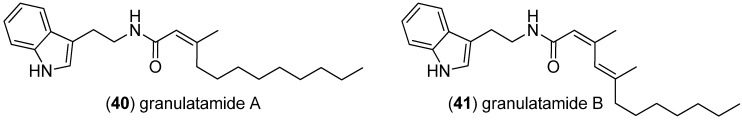
Granulatamides A and B.

Granulatamides A (**40**) and B (**41**) were isolated from the soft coral *Eunicella granulate* and showed cytotoxic activity against the tumor cell lines DU-145, LNCaP, SK-OV-3, IGROV, IGROV-ET, SK-BR3, SK-MEL-28, HMEC1, A549, K562, PANC1, HT-29, LOVO, LOVO-DOX, HeLa and HeLa-APL (GI_50_—concentration which effects a growth inhibition of 50%—1.7–13.8 μM) (Figure 8) [48].

**Figure 9:**
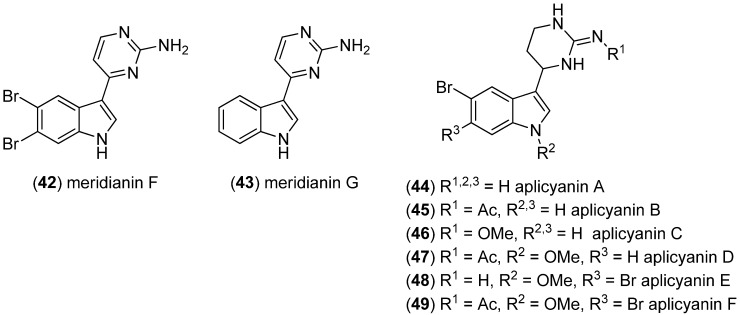
Meridianins F and G and aplicyanins A–F.

Meridianins F (**42**) and G (**43**) have been isolated and identified from the tunicate *Aplidium meridianum* by tandem mass spectrometry (Figure 9) [49]. Analysis of the extract of the related tunicate *Aplidium cyaneum*, collected in Antarctica, afforded bromoindole derivatives, aplicyanins A–F (**44**–**49**). *N-*Acetylated aplicyanins B, D and F were found to have potent antimitotic, as well as cytotoxic, activities against the tumor cell lines HT-29 (GI_50_ 0.39, 0.33, and 0.47 μM, respectively), A549 (GI_50_ 0.66, 0.63, and 1.31 μM, respectively) and MDA-MB-231 (GI_50_ 0.42, 0.41, and 0.81 μM, respectively), while aplicyanin E showed only mild cytotoxic effects (GI_50_ values 7.96–8.70 μM) [50,51].

**Figure 10:**
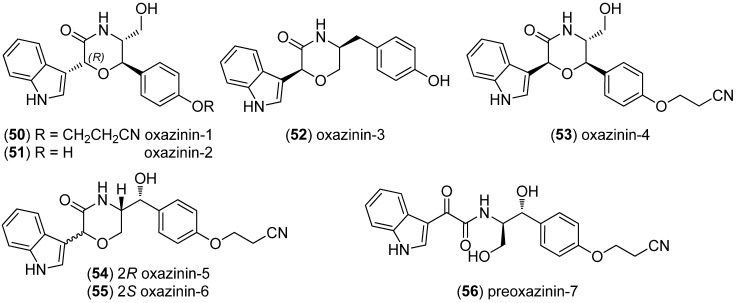
Oxazinins 1–7.

Oxazinins 1–6 (**50**–**55**) and their linear precursor preoxazinin-7 (**56**) were isolated from toxic edible mussels (*Mytilus galloprovincialis*) from the Northern Adriatic coast (Figure 10). The stereochemistry of oxazinins 1 and 2 was revised to 2*R* [52,53,54,55].

**Figure 11:**
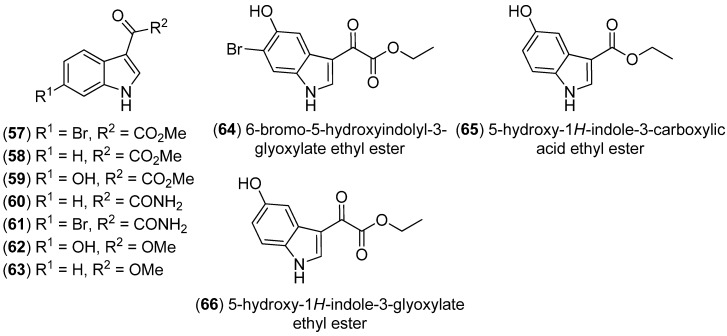
Indole-3-glyoxylates and indole-3-carboxylates.

Monoindole derivatives (**57**–**63**) were isolated from the marine sponge *Spongosorites sp.* by bioactivity-guided fractionation (Figure 11). Compound **63** displayed weak cytotoxic activity towards several human cancer cell lines. Those compounds, mostly indole pyruvic acid derivatives, are hypothetical biosynthetic precursors of co-occurring bisindoles, such as the hamacanthins and the topsentins, see Figure 98 [56]. 6-Bromo-5-hydroxyindolyl-3-glyoxylate ethyl ester (**64**) was isolated from the far eastern ascidian *Syncarpa oviformis* [57]. 5-Hydroxy-1*H*-indole-3-carboxylic acid ethyl ester (**65**) and 5-hydroxy-1*H*-indole-3-glyoxylate ethyl ester (**66**) were isolated from the marine sponge *Ircinia* sp., Irciniidae, collected at Iriomote Island [58].

**Figure 12:**
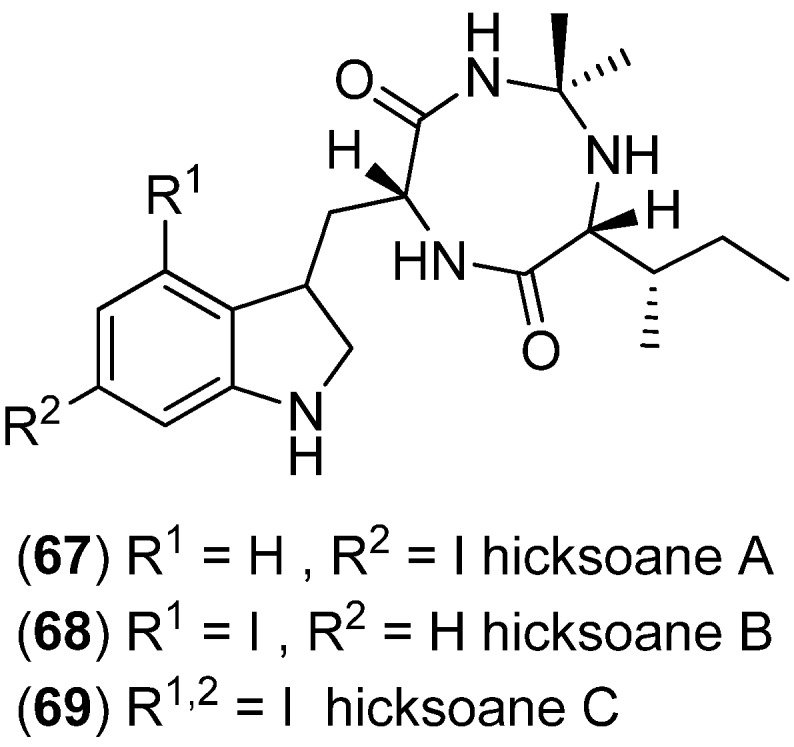
Hicksoanes A–C.

Iodinated heterocycles hicksoanes A–C (**67**–**69**) are derived from the gorgonian *Subergorgia hicksoni* and show antifouling effects (Figure 12) [59].

**Figure 13:**
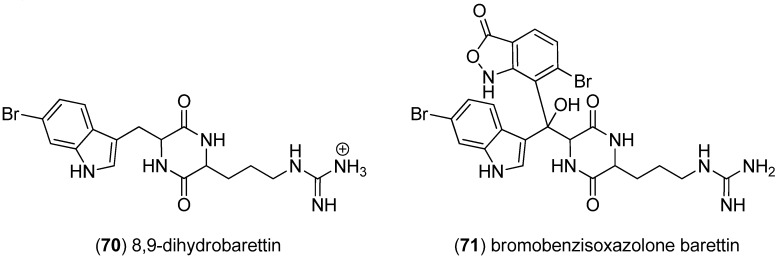
Barettin derivatives.

8,9-Dihydrobarettin (**70**) and bromobenzisoxazolone barettin (**71**), natural products with entirely unprecedented benzisoxazolone substructure were reported as isolates from the marine sponge *Geodia barretti* and showed inhibitory effects on the settlement of the barnacle larvae *Balanus improvises* (Figure 13) [60,61].

**Figure 14:**
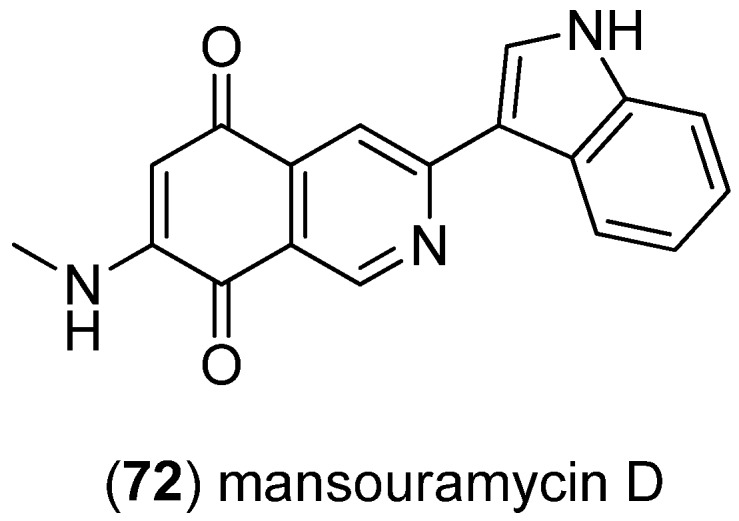
Mansouramycin D.

Analysis of the marine-derived *Streptomyces* sp. isolate Mei37 led to isolation of mansouramycins A–D with mansouramycin D (**72**), being the only indole-derived congener (Figure 14). Mansouramycins A–C display significant cytotoxic effects [62].

**Figure 15:**
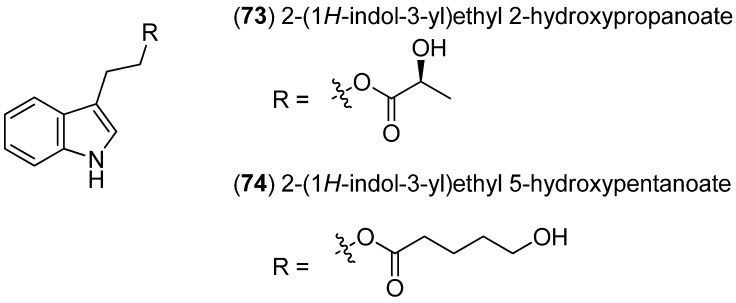
2-(1*H*-Indol-3-yl)ethyl 2-hydroxypropanoate and 2-(1*H*-indol-3-yl)ethyl 5-hydroxypentanoate.

With 2-(1*H*-indol-3-yl)ethyl 2-hydroxypropanoate (**73**) and 2-(1*H*-indol-3-yl)ethyl 5-hydroxypentanoate (**74**), two new indole derivatives were isolated from a marine sponge-derived yeast strain USF-HO25, identified as *Pichia membranifaciens* (Figure 15). Both compounds showed weak activity as 2,2-diphenyl-1-picrylhydrazyl (DPPH) radical scavengers [63].

**Figure 16:**
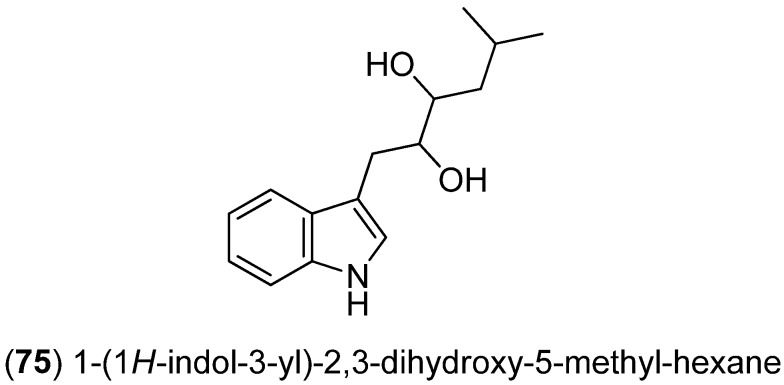
1-(1*H*-Indol-3-yl)-2,3-dihydroxy-5-methyl-hexane.

1-(1*H*-Indol-3-yl)-2,3-dihydroxy-5-methyl-hexane (**75**) was isolated from the South China Sea sponge *Axinella* sp. (Figure 16) [64].

**Figure 17:**
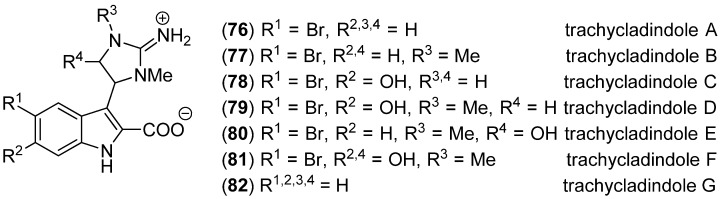
Trachycladindoles A–G.

Trachycladindoles A–G (**76**–**82**), isolated from the southern Australian marine sponge *Trachycladus laevispirulifer*, bear a 2-amino-4,5-dihydroimidazole moiety (Figure 17). They displayed a substitution pattern-dependent cytotoxic activity against human cancer cell lines A549, HT-29 and MDA-MB-231 [65].

**Figure 18:**
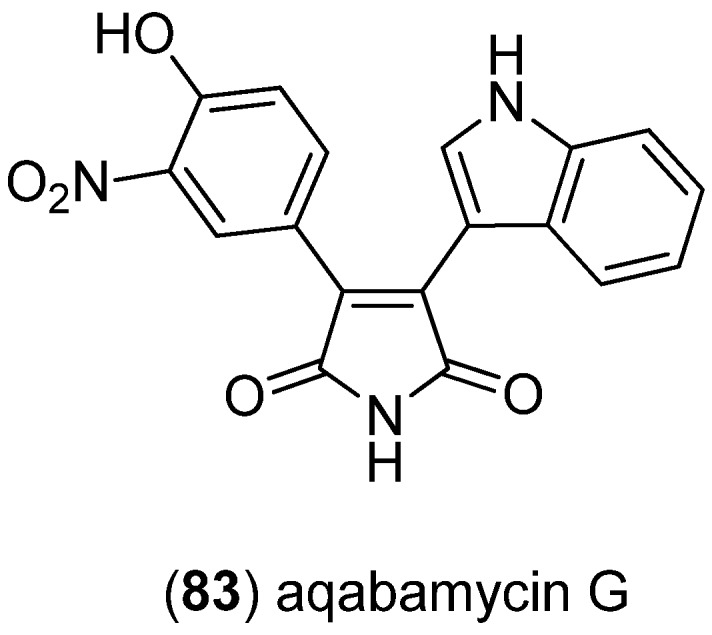
Aqabamycin G.

Aqabamycins A–G were isolated from fermentation broths of a marine bacterium, *Vibrio* sp., isolated from Red Sea soft coral *Sinularia polydactyla*, aqabamycin G (**83**) being the only indole derivative (Figure 18) [66,67].

**Figure 19:**
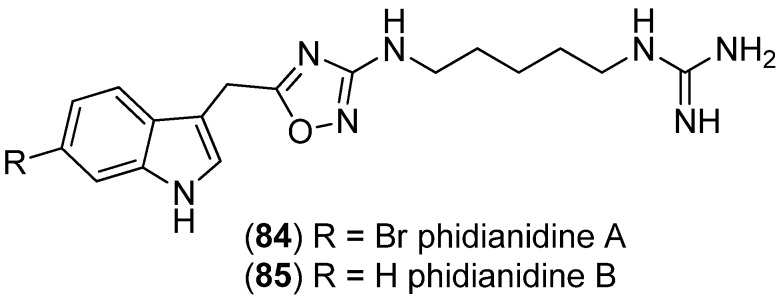
Phidianidines A and B.

Phidianidines A (**84**) and B (**85**) have been isolated from the marine opisthobranch mollusc *Phidiana militaris* and contain an uncommon 1,2,4-oxadiazole fragment (Figure 19) [68]. Both showed high cytotoxic activity towards several tumor cells [68], although other authors indicated a lack of cytoxicity [69,70]. Phidianidine A (**84**) was identified as a new CXCR4 (chemokine receptor) ligand and inhibits CXCL12 (C-X-C motif chemokine 12)-induced DNA synthesis, cell migration, and ERK1/2 (extracellular-signal-regulated kinases) activation [69]. Phidianidines displayed inhibitory activity on the dopamine transporter (DAT), but no activity towards the norepinephrine (NET) and the serotonin transporter (SERT). Furthermore, they represent selective and potent ligands of the μ-opioid receptor with no activity on δ- or κ-opioid receptors [70]. Phidianidine analogs were tested as neuroprotective agents, showing activity against Aβ_25–35_-, H_2_O_2_- and OGD (oxygen-glucose deprivation)-induced neurotoxicity in SH-SY5Y cells [71].

**Figure 20:**
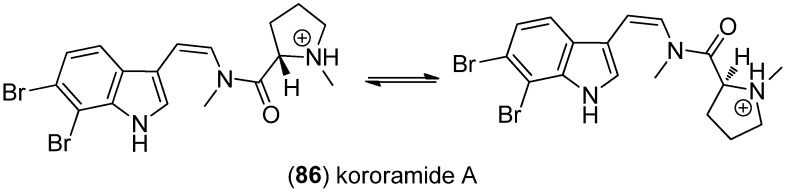
Koroamide A, mixture of *cis*- and *trans* amide rotamer.

Kororamide A (**86**) was obtained from the Australian bryozoan *Amathia tortuosa* and exists as a mixture of its *cis* and *trans* amide rotamer (Figure 20) [72].

**Figure 21:**
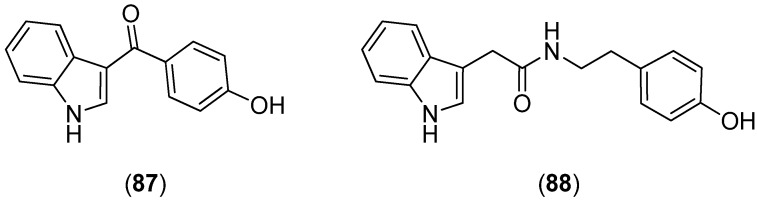
3-(*p*-Hydroxy)benzoyl indole and *N*-(4-hydroxyphenethyl)-2-(1*H*-indol-3-yl)acetamide.

Analysis of the marine bacterium *Pantoea agglomerans* P20-14 resulted in the isolation of 3-(*p*-hydroxy)benzoyl indole (**87**) and *N*-(4-hydroxyphenethyl)-2-(1*H*-indol-3-yl)acetamide (**88**) (Figure 21), together with bisindole 1,2-di(1*H*-indol-3-yl)ethane (**506**, see Figure 84) [73].

**Figure 22:**
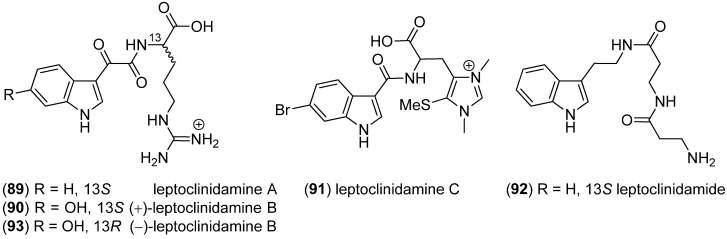
Leptoclinidamines A–C and leptoclimidamide.

Leptoclinidamines A–C (**89**–**91**) were isolated from the Australian ascidian *Leptoclinides durus* (Figure 22)*.* None of the compounds was active in antimalarial, antitrypanosomal and cytotoxic activity tests [74]. Leptoclinidamide (**92**) and (–)-leptoclinidamine B (**93**) were isolated from the Indonesian ascidian *Leptoclinides dubius*, together with C^2^-α-d-mannosylpyranosyl-l-tryptophan (**9**). They did not exhibit antifungal, antibacterial (Gram-positive and Gram-negative) or cytotoxic (HCT-15 and Jurkat cell lines) activity [75].

**Figure 23:**
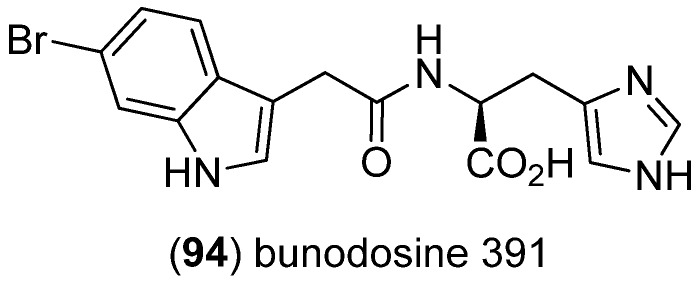
Bunodosine 391 (BDS 391).

Bunodosine 391 (BDS 391, **94**) was isolated from the sea anemone *Bunodosoma cangicum* and showed analgetic effects mediated via serotonin receptors (Figure 23) [76].

**Figure 24:**
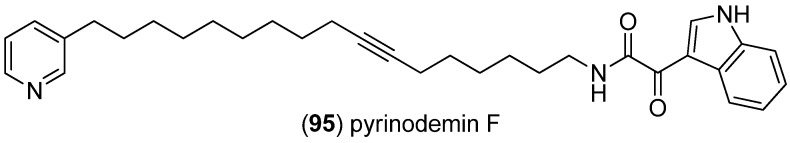
Pyrinodemin F.

Pyrinodemin F (**95**) was obtained from an Okinawan marine sponge *Amphimedon* sp. (Figure 24) [77].

**Figure 25:**
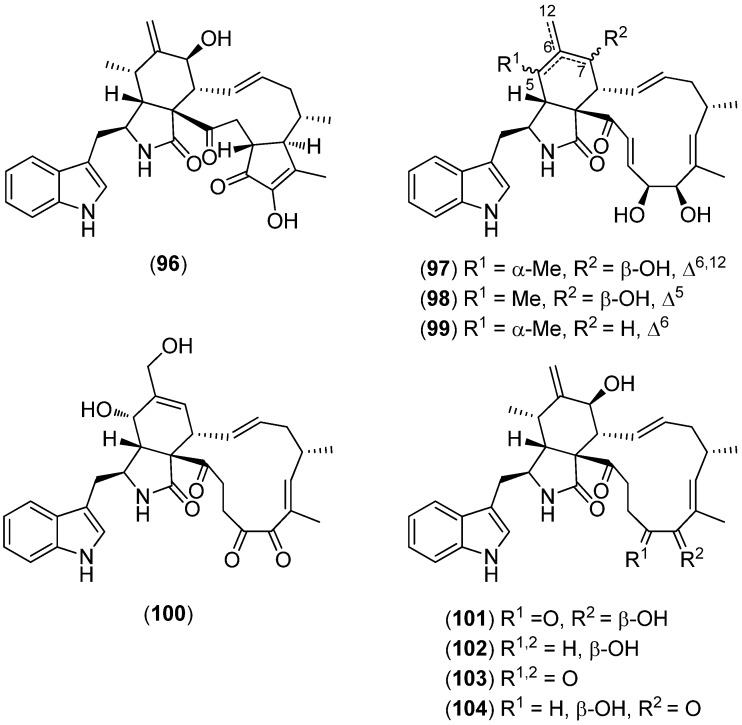
Cytoglobosins A–G, isochaetoglobosin D and chaetoglobosin F_ex_.

Cytoglobosins A–G (**96**–**102**), isochaetoglobosin D (**103**) and chaetoglobosin F_ex_ (**104**) were isolated from the marine green alga derived endophytic fungus *Chaetomium globosum* QEN-14 (Figure 25). Cytoglobosins C (**98**) and D (**99**) exhibited cytotoxic activity towards the cancer cell line A549 (IC_50_-values 2.26 and 2.55 μM), whereas the other cytoglobosins were inactive [78].

**Figure 26:**
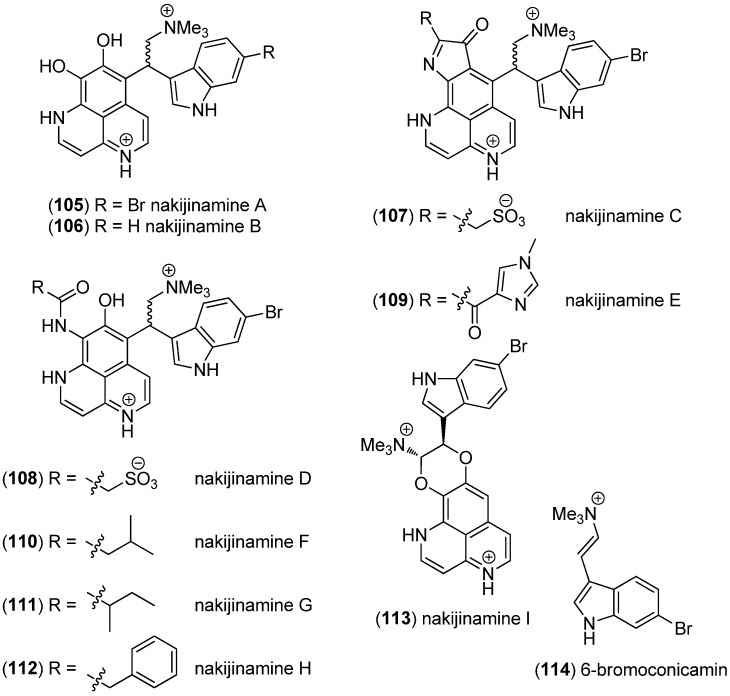
Nakijinamines A–I and 6-bromoconicamin.

Nakijinamines A–I (**105**–**113**) and 6-bromoconicamin (**114**) have been isolated from the Okinawan marine sponge *Suberites* sp. (Figure 26). Nakijinamine A (**105**) showed antimicrobial activity against *Candida albicans*, *Cryptococcus neoformans,* *Trichophyton mentagrophytes,* *Staphylococcus aureus,* *Bacillus subtilis* and *Micrococcus luteus*. Nakijinamines C (**107**) and E (**109**) exhibited antifungal activity against *Aspergillus niger*, while nakijinamines B (**106**) and F (**110**) showed activity against *C. albicans*. None of the compounds showed cytotoxicity against mammalian cells [79,80].

**Figure 27:**
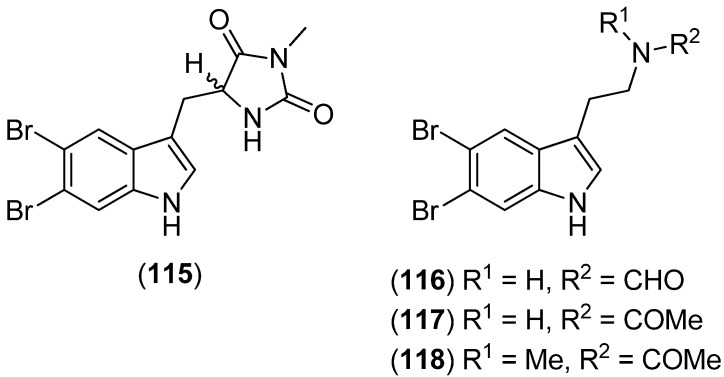
5,6-Dibromoindole derivatives.

5′-[(5,6-Dibromo-1*H*-indol-3-yl)methyl]-3′-methylimidazolidine-2′,4′-dione (**115**), 5,6-dibromo-1*H*-indole-3-ethyl-*N*-formylamine or 5,6-dibromo-*N*-formyltryptamine (**116**), 5,6-dibromo-1*H*-indole-3-ethyl-*N*-acetylamine or 5,6-dibromo-*N*-acetyltryptamine (**117**) and 5,6-dibromo-*N*-acetyl-*N*-methyltryptamine (**118**) were obtained from the Thai sponge *Smenospongia* sp. (Figure 27) [81].

Penilloid A (**119**) was isolated from the marine derived fungi *Penicillium* sp. and *A. sydowii* [82].

**Figure 28:**
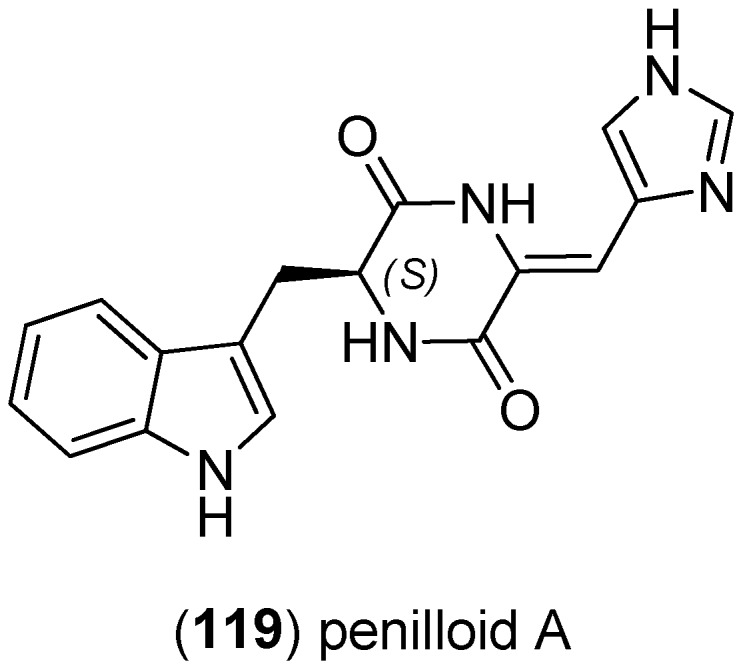
Penilloid A.

**Figure 29:**
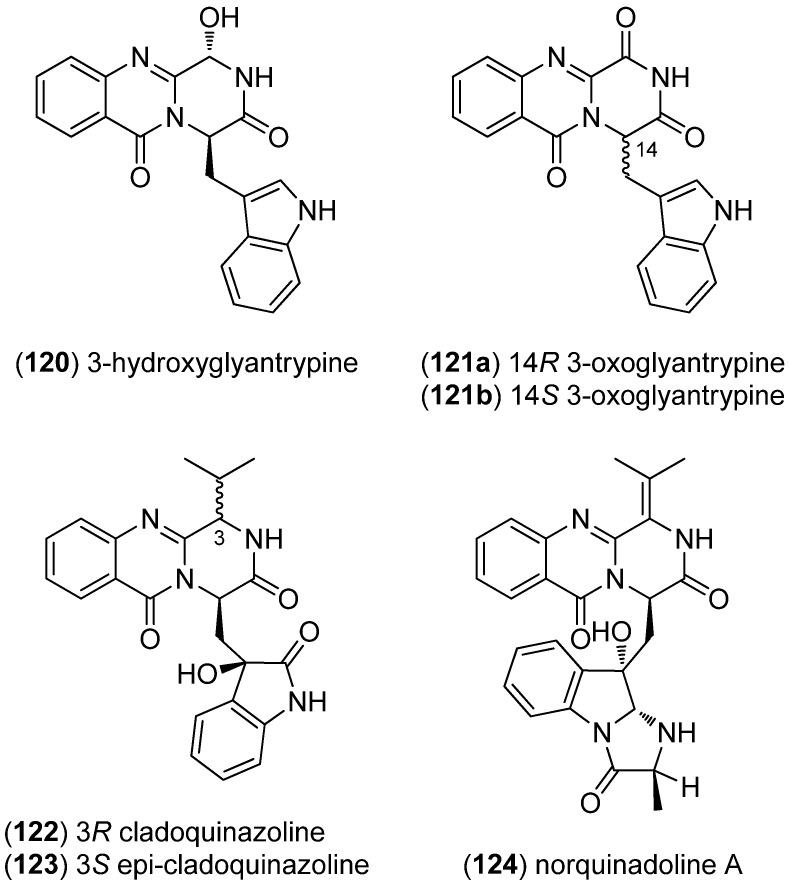
Glyantrypine derivatives, cladoquinazoline, *epi-*cladoquinazoline and norquinadoline A.

3-Hydroxyglyantrypine (**120**), oxoglyantrypine (**121**a, **121**b), cladoquinazoline (**122**), *epi*-cladoquinazoline (**123**) and norquinadoline A (**124**) were isolated from the mangrove-derived fungus *Cladosporium* sp. PJX-41 and exhibited antiviral activities against influenza A virus (H_1_N_1_) (Figure 29) [83].

**Figure 30:**
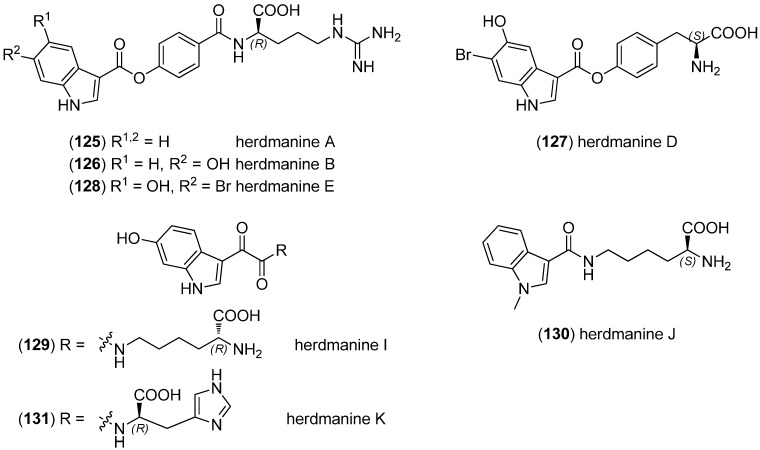
Herdmanines A–K.

Herdmanines A–D were isolated from the marine ascidian *Herdmania momus*, herdmanines A, B and D (**125**–**127**) being tryptophan-derived (Figure 30). Herdmanines C and D (**127**) showed anti-inflammatory activities, due to the inhibitory effect on mRNA expression of iNOS (inducible nitric oxide synthase) as well as COX-2 (cyclooxygenase 2) and IL-6 (interleukin 6) [84]. Herdmanines E–L were isolated from the same organism and displayed peroxisome proliferator-activated receptor (PPAR)-γ agonistic activity. Herdmanines E and I–K (**128**–**131**) are tryptophan-derived [85]. Herdmanines A, B, E, I and K are derived from d-amino acids.

**Figure 31:**
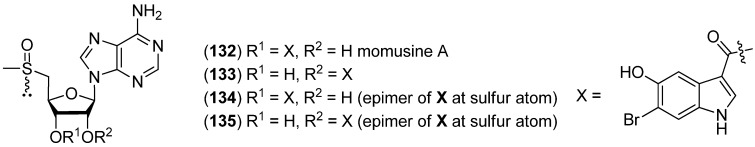
Momusines.

The ascidian *Herdmania momus* was also a source of nucleosides (**132**–**135**), compound **132** was given the trivial name momusine A (Figure 31). None of the compounds exhibited antiviral activity against a series of human pathogenic viruses [86].

**Figure 32:**
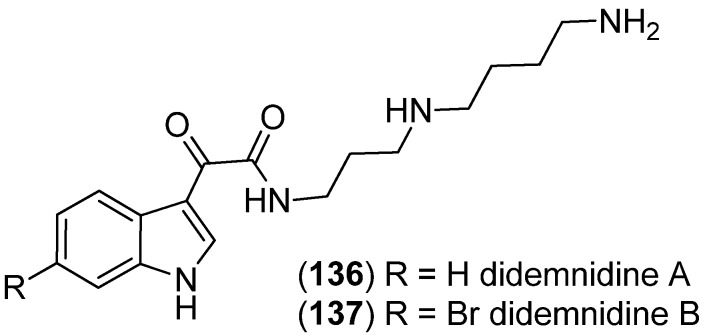
Didemnidines A and B.

Didemnidines A (**136**) and B (**137**), two indole spermidine alkaloids, were isolated from the New Zealand ascidian *Didemnum* sp. (Figure 32). Both were found to be inactive as phospholipase A_2_ and farnesyltransferase enzyme inhibitors and not cytotoxic, but didemnidine B (**137**) showed mild antiparasitic activity against the malaria parasite *Plasmodium falciparum* [87].

**Figure 33:**
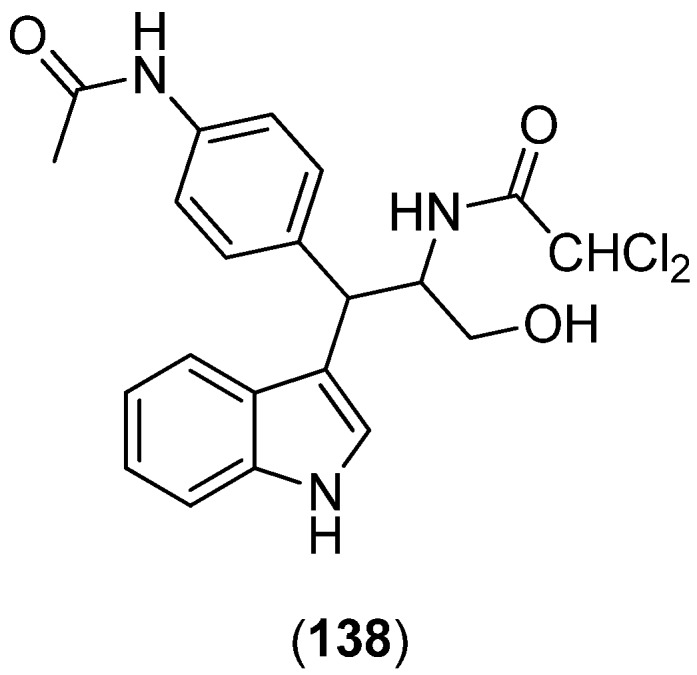
*N*-{1-[4-(Acetylamino)phenyl]-3-hydroxy-1-(1*H*-indol-3-yl)propan-2-yl}-2,2-dichloroacetamide.

*N*-{1-[4-(Acetylamino)phenyl]-3-hydroxy-1-(1*H*-indol-3-yl)propan-2-yl}-2,2-dichloroacetamide (**138**) was isolated from a deep-sea sediment metagenomic clone-derived *Escherichia coli* fermentation broth and found to have analgetic activity (Figure 33) [88]. It has a remarkable structural resemblance to the antibiotic chloramphenicol.

**Figure 34:**
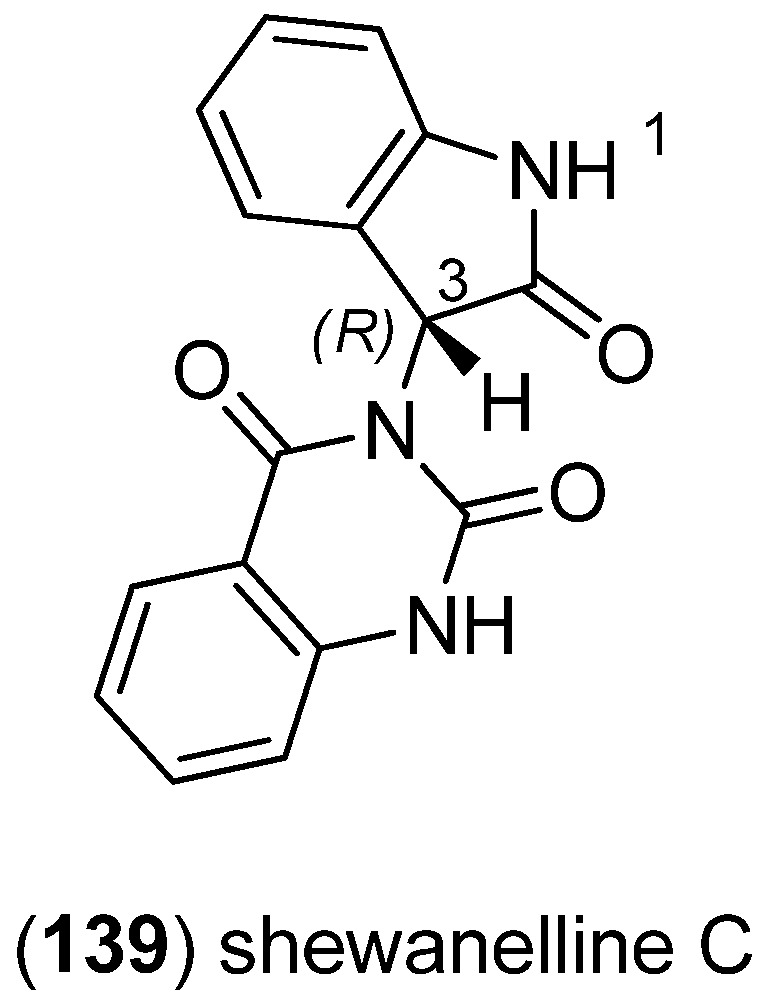
Shewanelline C.

Fermentation of deep-sea bacterium *Shewanella piezotolerans* WP3 yielded three new indole alkaloids namely shewanellines A–C. Shewanellines A (**507**) and B (**508**) belong to the bisindole alkaloids (see Figure 85). Shewanelline C (**139**) displayed cytotoxic activity against the tumor cell lines HL-60 and BEL-7402 (IC_50_ 5.91 and 10.03 μg/mL, respectively) (Figure 34) [89].

**Figure 35:**
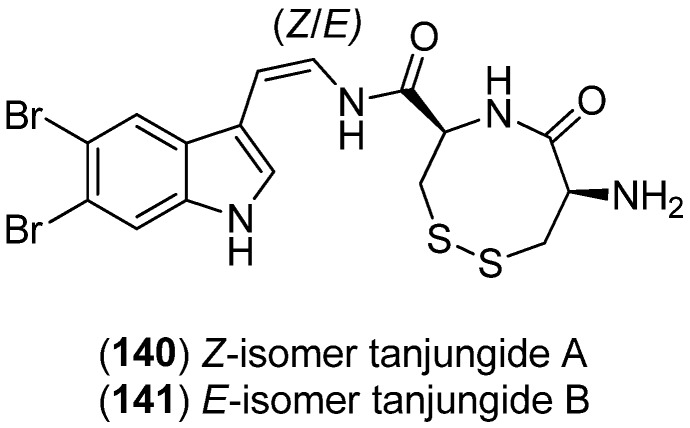
Tanjungides A and B.

Tanjungides A (**140**) (*Z* isomer) and B (**141**) (*E* isomer), two dibrominated indole enamides, have been isolated from the tunicate *Diazona* cf. *Formosa* and were found to have significant cytotoxicity against human tumor cell lines (Figure 35). In the same publication, the first total synthesis of these compounds is reported employing methyl 1*H*-indole-3-carboxylate as starting material [90].

**Figure 36:**
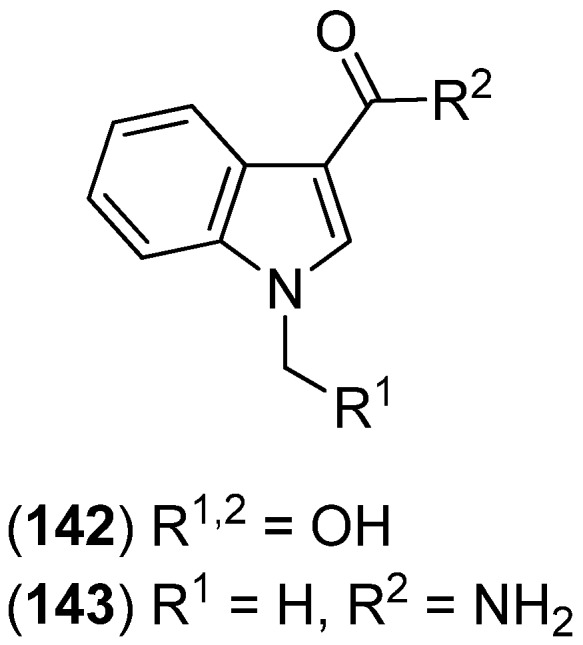
1-Hydroxymethylindole-3-carboxylic acid and 1-methyl indole-3-carboxamide.

Examination of actinomycete *Actinomadura* BCC 24717 led to the isolation of 1-hydroxymethylindole-3-carboxylic acid (**142**) and 1-methyl indole-3-carboxamide (**143**) with the latter compound displaying antifungal activity against *Candida albican*s (IC_50_ 41.97 μg/mL) (Figure 36) [91].

**Figure 37:**
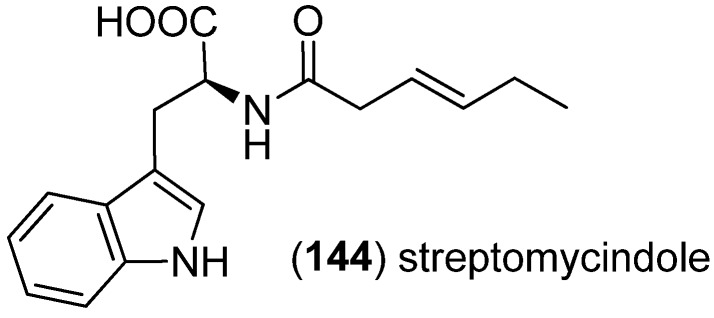
Streptomycindole.

Streptomycindole (**144**) was isolated from *Streptomyces* sp*.* DA22, associated with the South China Sea sponge *Craniella australiensis*, and was found to be inactive against the tumor cell lines HL-60, HCT-116, HO-8910 and HepG2 (Figure 37) [92].

**Figure 38:**
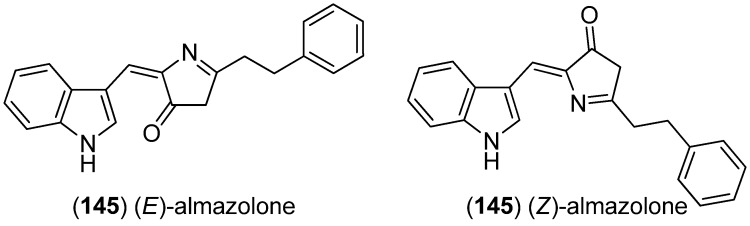
Almazolone.

Almazolone (**145**) was isolated from the red alga *Haraldiophyllum* sp., collected in Dakar (Senegal) as an 88:12 mixture of (*Z*)/(*E*) stereoisomers (Figure 38). Photoisomerization of the (*Z*) into the (*E*)-isomer, as well as slow thermal reisomerization of the (*E*)-isomer, was observed [93].

**Figure 39:**
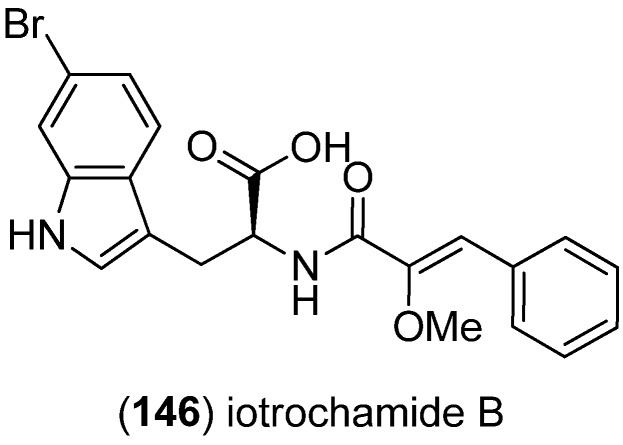
Iotrochamide B.

Iotrochamides A and B were isolated from the Australian sponge *Iotrochota* sp., iotrochamide B (**146**) bearing an indole moiety (Figure 39). Both compounds show inhibitory effects against the human pathogenic protozoon *Trypanosoma brucei brucei* (IC_50_ 3.4 and 4.7 μM, respectively) [94].

**Figure 40:**
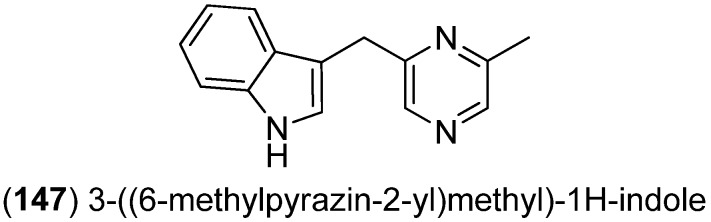
3-((6-Methylpyrazin-2-yl)methyl)-1*H*-indole.

3-((6-Methylpyrazin-2-yl)methyl)-1*H*-indole (**147**) was isolated from the deep-sea actinomycete *Serinicoccus profundi* sp. nov. and exhibited weak antimicrobial activity against *Staphylococcus aureus* ATCC 25923 but no cytotoxic effects against BEL7402 and HL-7702 cell lines (Figure 40) [95].

**Figure 41:**
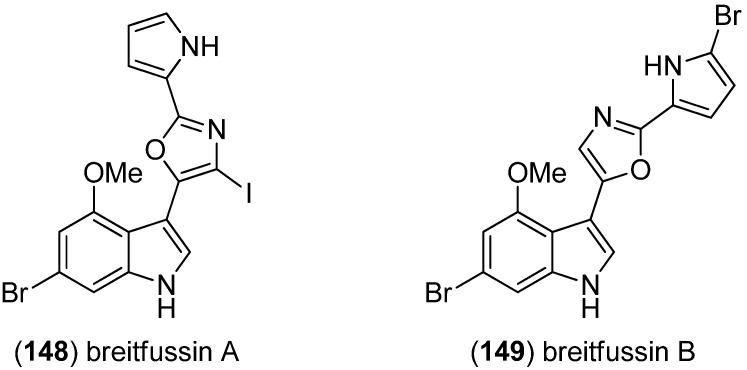
Breitfussins A and B.

Breitfussins A (**148**) and B (**149**) were isolated from the Arctic hydrozoan *Thuiaria breitfussi* (Figure 41) [96].

**Figure 42:**
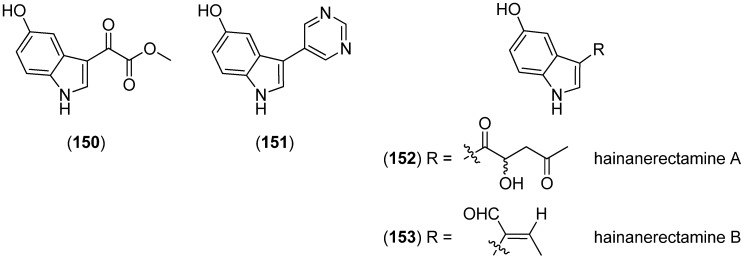
5-Hydroxyindole-3-glyoxylate methyl ester, hyrtinadine B and Hainanerectamines.

5-Hydroxyindole alkaloids 5-hydroxyindole-3-glyoxylate methyl ester (**150**) and (**151**), together with the bisindole scalaridine A (**505**, see Figure 83), were isolated from the marine sponge *Scalarispongia* sp. collected near Dokdo island (Figure 42). Since **151** was the monoindole analog of hyrtinadine A (**496**, see Figure 80), it was named hyrtinadine B [97]. Hainanerectamines A–C (**152**, **153** and **X**) have been isolated from the Hainan marine sponge *Hyrtios erectus*, hainanerectamine C (**800**) belonging to the group of β-carboline alkaloids (see Figure 147). Hainanerectamines B (**153**) and C (**800**) display moderate inhibitory effects on the serine/threonine kinase Aurora A (IC_50_ 24.5 and 18.6 μg/mL), which is involved in cell division regulation, but none of the compounds had cytotoxic effects on the tumor cell lines A549 and HT-29 [98].

### 2.2. Prenylated Indoles

**Figure 43:**
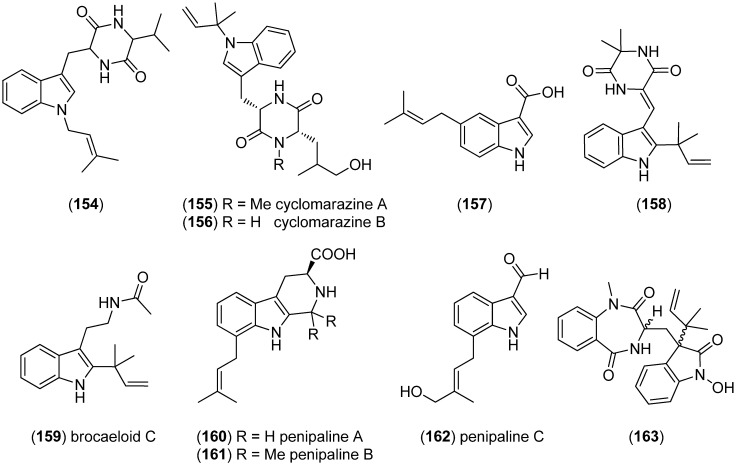
Simple prenylated indole alkaloids.

Prenylated indole alkaloids represent a large subgroup of the indole alkaloids and provide various potent biological activities. Their wide distribution in terrestrial and marine organisms nicely reflects the high nucleophilicity of the indole core which is an adequate match for the electrophilic reactivity of prenyl-type electrophiles generated from the corresponding pyrophosphates [99]. Biosynthetically, tryptophan is the indole source in most cases [100].

(Indole-*N*-isoprenyl)-tryptophan-valine diketopiperazine (**154**) was isolated from the M-3 strain belonging to the *Ascomycota* phylum and was shown to have a strong and selective antifungal activity against *Pyricularia oryzae* (Figure 43) [101]. Cyclomarazines A (**155**) and B (**156**) were isolated from the marine bacterium *Salinispora arenicola* CNS-205 and exhibited antimicrobial activities [102]. 5-Dimethylallylindole-3-carboxylic acid (**157**) was obtained from the marine-derived *Streptomyces* sp. MS239 and did not show antibacterial activity [103]. Dipodazine derivative (**158**) was isolated from the mangrove-derived endophytic fungus *Penicillium chrysogenum* MTCC 5108 and showed antibacterial activity [104]. Brocaeloids A–C were obtained from cultures of *Penicillium brocae* MA-192, an endophytic fungus isolated from the fresh leaves of the marine mangrove plant *Avicennia marina*. They contain a C-2 reversed prenylation, but only brocaeloid C (**159**) is tryptophan-derived. It showed weak or no antibacterial or DPPH radical scavenging activity and no lethality against brine shrimp (*Artemia salina*) [105]. Penipalines A–C (**160**–**162**) were isolated from the deep-sea-sediment derived fungus *Penicillium paneum* SD-44. Penipalines B (**161**) and C (**162**) showed cytotoxic effects against the A549 (IC_50_ 20.44 and 21.54 μM) and HCT-116 (IC_50_ 14.88 and 18.54 μM) tumor cell lines [106]. 3-((1-Hydroxy-3-(2-methylbut-3-en-2-yl)-2-oxoindolin-3-yl)methyl)-1-methyl-3,4-dihydro-benzo[*e*][1,4]diazepine-2,5-dione (**163**) was obtained from the Mediterranean sponge-derived fungus *Aspergillus* sp. [107].

**Figure 44:**
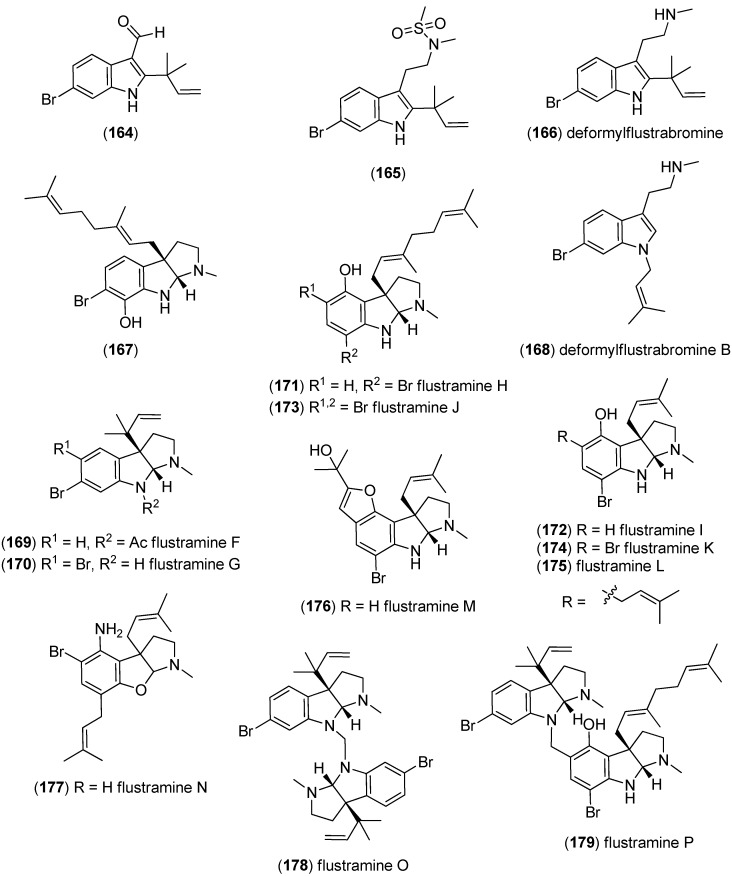
Prenylated indole alkaloids from *Flustra foliacea*.

Beginning in 1979, a group of brominated and prenylated indole alkaloids, named flustramines, have been isolated from the marine bryozoan *Flustra foliacea* [108,109,110], some of them having cytotoxic and antimicrobial [111], muscle relaxant [112], as well as butyrylcholinesterase (BChE) inhibitory effects [113]. From the same organism, several new alkaloids have been isolated, namely 6-bromo-2-(1,1-dimethyl-2-propenyl)-1*H*-indole-3-carbaldehyde (**164**), *N*-(2-[6-bromo-2-(1,1-dimethyl-2-propenyl)-1*H*-indol-3-yl]ethyl)-*N*-methyl-methanesulfonamide (**165**), deformylflustrabromine (**166**), (3a*R*′,8a*R*′)-6-bromo-3a-[(2*E*)-3,7-dimethyl-2,6-octadienyl]-1,2,3,3a,8,8a-hexahydropyrrolo[2,3-*b*]-indol-7-ol (**167**) and deformylflustrabromine B (**168**) (Figure 44) [114,115]. Deformylflustrabromine (**166**) and several synthetic derivatives were found to have inhibitory effects on bacterial indole signaling and, therefore, inhibit biofilm formation in *Escherichia coli* and *Staphylococcus aureus* [116] Deformylflustrabromine (**166**) and deformylflustrabromine B (**168**) show high and subtype selective affinity to nicotinic acetylcholine (nACh) receptors [114,117]. Flustramines F–P (**169**–**179**) have recently been isolated from *F. foliacea*, flustramines O (**178**) and P (**179**) being dimers, which may possibly be isolation artifacts [118].

**Figure 45:**
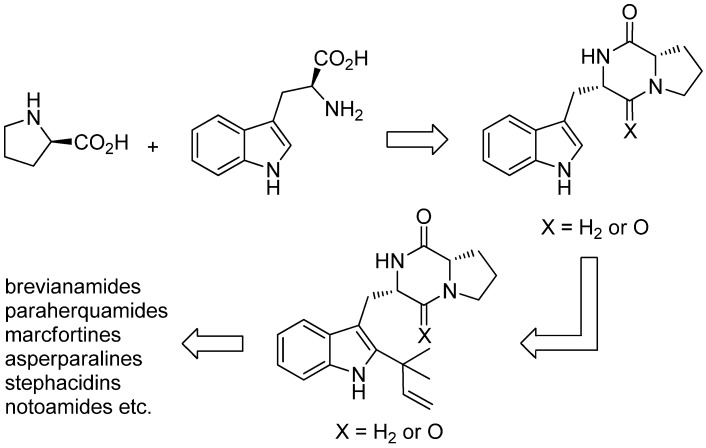
Biosynthetic pathway to brevianamides, paraherquamides, marcfortines, asperparalines, stephacidins and notoamides.

**Figure 46:**
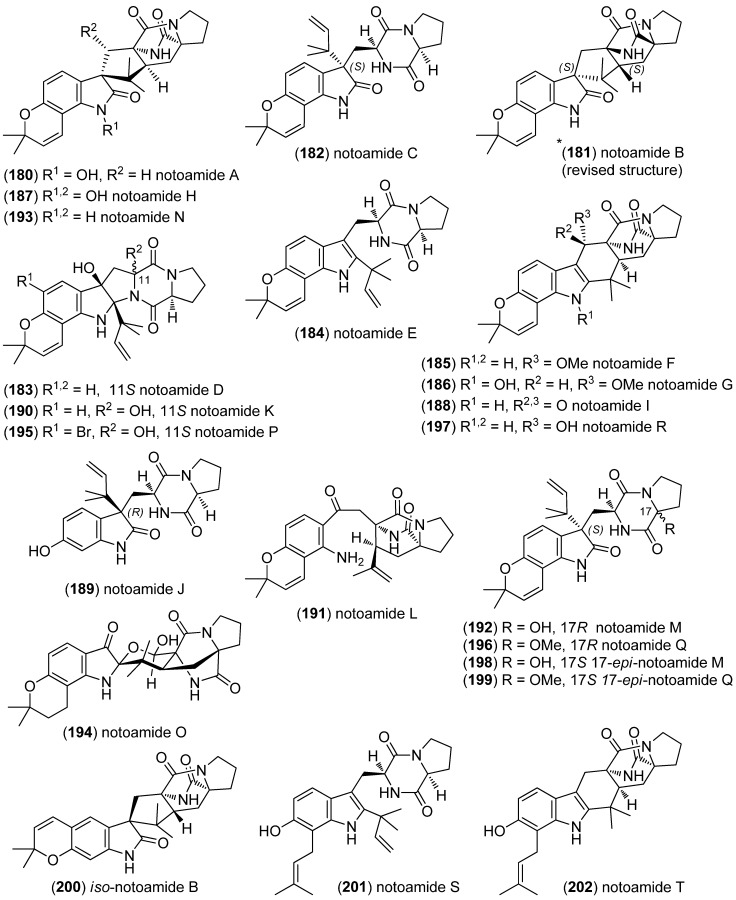
Notoamides A–T and *iso*-notoamide B.

**Figure 47:**
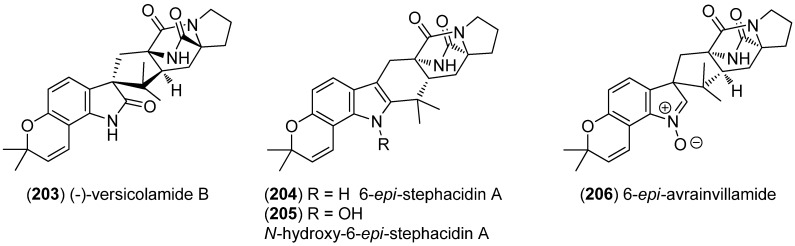
(–)-Versicolamide B, 6-*epi*-stephacidin A, *N*-hydroxy-6-*epi*-stephacidin A and 6-*epi*-avrainvillamide.

Notoamides are a class of prenylated indole alkaloids derived from proline, tryptophan and one or two isoprene units. They are closely related to brevianamides, paraherquamides, marcfortines, asperparalines and stephacidins (Figure 45) [119,120], which show cytotoxic [121], insecticidal [122], antibiotic and antiparasitic [123,124] activities. Notoamides A–D (**180**–**183**) were isolated from a mussel-derived *Aspergillus* species (Figure 46). Notoamides A–C showed moderate cytotoxicity against HeLa and L1210 cell lines (IC_50_ 22–52 μg/mL) and notoamide C induces G2/M-cell cycle arrest at 6.3 μg/mL (cell line not specified) [125]. Notoamide E (**184**) was identified to be a short-lived precursor in the biosynthesis of prenylated indole alkaloids in *Aspergillus* sp. and a feeding experiment of ^13^C-labeled notoamide E afforded structurally novel metabolites [126]. Notoamides F–R (**185**–**197**) [127,128,129], 17-*epi*-notoamides M (**198**) and Q (**199**) [130] and (–)-versicolamide B (**203**, Figure 47) [128] have been isolated from *Aspergillus* sp. Noteworthy are notoamide O (**194**) with its unprecedented hemiacetal/hemiaminal ether structure and notoamide P (**195**) as the first brominated member of the notoamides family. The absolute configuration of notoamide C (**182**) was first reported as 3*R*, but later revised to as 3*S* on the basis of X-ray crystallography and CD spectroscopy [130,131]. Thereafter, the configuration of notoamides Q (**196**) and M (**192**) was also corrected to 3*S* [132] as was the configuration of notoamide B (**181**) [130]. The absolute configuration of notoamide J was revised to be 3*R* [133]. *Iso*-notoamide B (**200**) was isolated from the marine-derived endophytic fungus *Paecilomyces variotii* EN-291 [134]. Notoamide C (**182**) displayed significant anti-fouling activity and strong antilarval settlement activity against *Bugula neritina* [135]. Notoamide I (**188**) was found to show weak cytotoxicity against HeLa cells (IC_50_ 21 µg/mL) [127]. Recently, notoamide S (**201**), which has been assumed to be a key intermediate in the biosynthesis of stephacidin and notoamide families, has been isolated from *Aspergillus amoenus* [120,136]. Notoamide T (**202**) was furthermore identified to be a biosynthetic precursor of stephacidin A and notoamide B, as proven by ^13^C feeding experiments [20,137]. Biosynthetic pathways of the notoamide, paraherquamide and malbrancheamide family are discussed by Li *et al.* [138].

**Figure 48:**
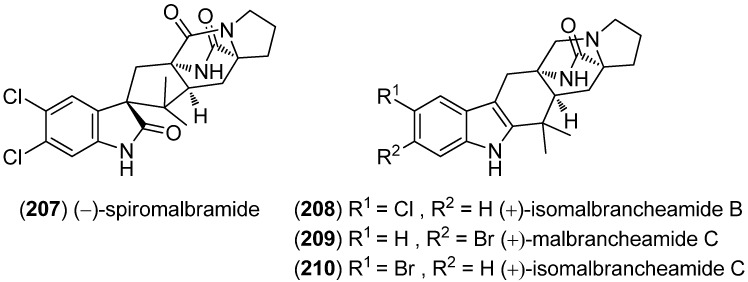
(−)-Spiromalbramide and malbrancheamides.

6-*epi*-Stephacidin A (**204**), *N*-hydroxy-6-*epi*-stephacidin A (**205**) and 6-*epi*-avrainvillamide (**206**) were isolated from *Aspergillus taichungensis* (Figure 47). *N*-Hydroxy-6-*epi*-stephacidin A (**205**) and 6-*epi*-avrainvillamide (**206**) showed significant cytotoxic activities against HL-60 (IC_50_ 4.45 and 1.88 μM) and A549 (IC_50_ 3.02 and 1.92 μM) cell lines [139].

(−)-Spiromalbramide (**207**) and (+)-isomalbrancheamide B (**208**) were identified from a marine invertebrate-derived *Malbranchea graminicola* strain, using direct analysis in real time (DART) mass spectrometry (Figure 48). Furthermore, (+)-malbrancheamide C (**209**) and (+)-isomalbrancheamide C (**210**) were isolated after enriching the growth medium with bromide salts [140]. Their structures resemble those of the versciolamides, the sclerotiamides and certain notoamides but the benzoid part of the indole unit is not prenylated.

**Figure 49:**
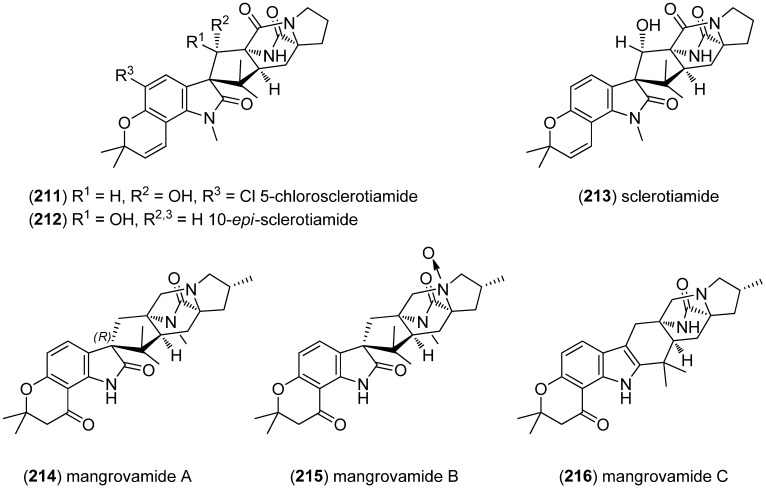
Sclerotiamides and mangrovamides A–C.

5-Chlorosclerotiamide (**211**) and 10-*epi*-sclerotiamide (**212**), together with sclerotiamide (**213**) and notoamide C (**182**) and I (**188**), were isolated from the deep-sea-derived fungus *Aspergillus westerdijkiae* DFFSCS013 (Figure 49). None of them showed cytotoxic effects against A549, HL-60, K562, and MCF-7 cell lines [141]. 5-Chlorosclerotiamide (**211**), together with brevianamide F, circumdatin F and L and notoamide C (**182**) were found to have antifouling potential and displayed potent activity against larval settlement of *Bugula neritina* [135]. Mangrovamides A–C (**214**–**216**) have been isolated from *Penicillium* sp. isolated from a mangrove sediment sample of the South China Sea. They bear a γ-methyl proline derived skeleton, unprecedented among the paraherquamide family. Mangrovamides did not show cytotoxicity against the human tumor cell lines H1975, U937, K562, BGC823, MOLT-4, MCF-7, A549, HeLa, HL-60 and Huh-7, while mangrovamide C (**216**) displayed moderate inhibitory effect on AChE (IC_50_ 58.0 μM) [142].

**Figure 50:**
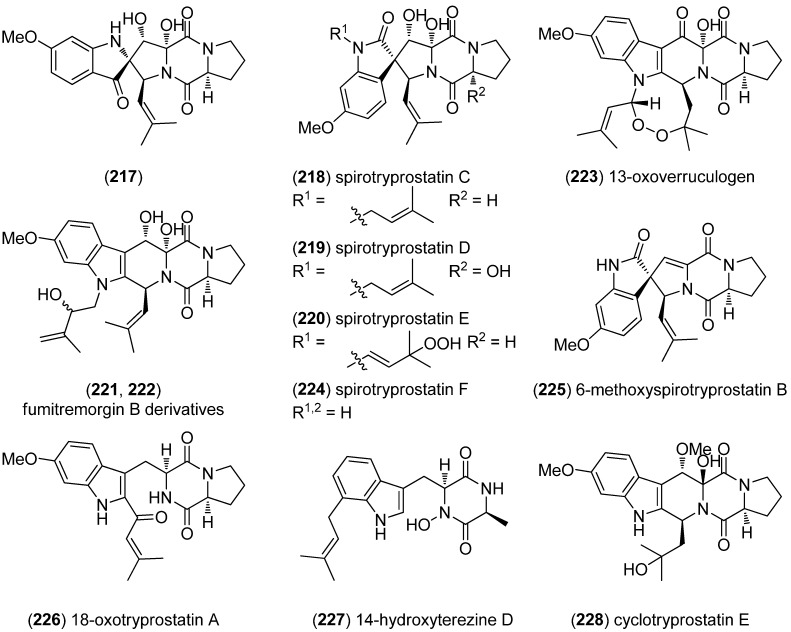
Prenylated indole diketopiperazine alkaloids.

With compound **217**, spirotryprostatins C–E (**218**–**220**), two fumitremorgin B derivatives (**221**, **222**) and 13-oxoverruculogen (**223**), new prenylated indole diketopiperazine alkaloids have been isolated from the holothurian-derived fungus *Aspergillus fumigatus* (Figure 50)*.* All substances were tested for cytotoxic activity against MOLT-4, A549, HL-60, and BEL-7420 cell lines and showed variable activity with spirotryprostatin E and fumitremorgin B derivatives being most active [143]. Spirotryprostatin F (**224**) was also isolated from *A. fumigatus* [144]. *Aspergillus sydowi* PFW1-13 was the source of 6-methoxyspirotryprostatin B (**225**), 18-oxotryprostatin A (**226**) and 14-hydroxyterezine D (**227**). They show weak cytotoxic activity towards A549 (IC_50_-values 8.29, 1.28 and 7.31 µM, respectively) and 14-hydroxyterezine D (**227**) was also active against HL-60 cells (IC_50_ 9.71 µM) [145]. Cyclotryprostatin E (**228**) was obtained from the marine fungal strain *Aspergillus sydowii* SCSIO 00305, isolated from a healthy tissue of *Verrucella umbraculum* [146].

**Figure 51:**
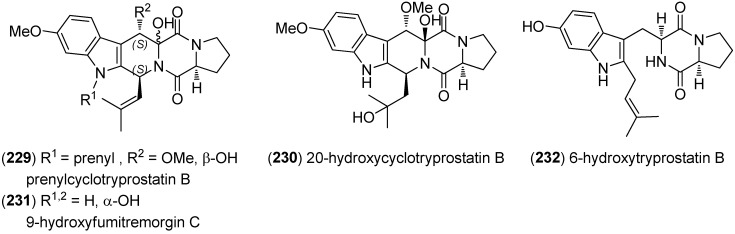
Prenylated indole diketopiperazine alkaloids.

Prenylcyclotryprostatin B (**229**), 20-hydroxycyclotryprostatin B (**230**), 9-hydroxyfumitremorgin C (**231**) and 6-hydroxytryprostatin B (**232**) were obtained from *A. fumigatus* YK-7 (Figure 51). Prenylcyclotryprostatin B (**229**) and 9-hydroxyfumitremorgin C (**231**) displayed cytotoxic activities towards U937 cell lines [147].

**Figure 52:**
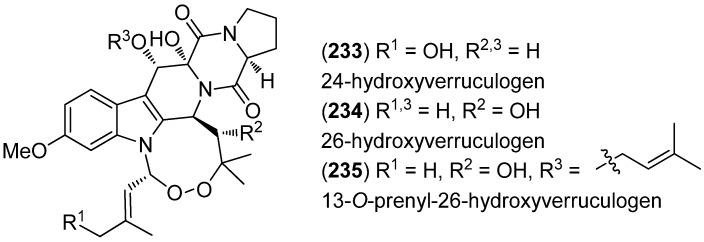
Prenylated indole diketopiperazine alkaloids.

24-Hydroxyverruculogen (**233**), 26-hydroxyverruculogen (**234**) and 13-*O*-prenyl-26-hydroxyverruculogen (**235**) have been isolated from the marine sediment-derived fungus *Penicillium brefeldianum* SD-273 (Figure 52). None of them exhibited pronounced antibacterial or cytotoxic effects, but 13-*O*-prenyl-26-hydroxyverruculogen showed a potent insecticidal activity against brine shrimp (*Artemia salina*, LC_50_—concentration which is lethal to 50% of the test organisms—9.44 μΜ) [148].

**Figure 53:**
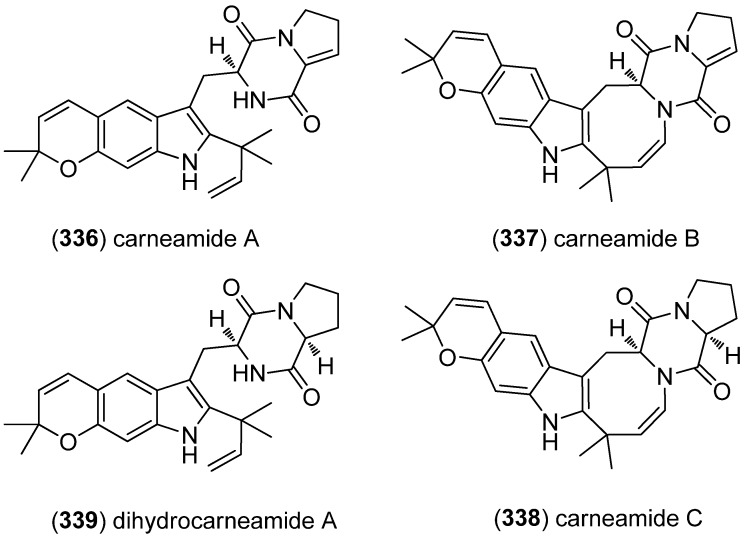
Carneamides A–C and dihydrocarneamide A.

The diketopiperazine alkaloids carneamides A–C (**336**–**338**) were obtained from the marine-derived fungus *Aspergillus carneus* (*Trichocomaceae*) KMM 4638 (Figure 53). Carneamides B (**337**) and C (**338**) bear the rare indoloazocine subunit [149]. Dihydrocarneamide A (**339**), together with *iso*-notoamide B (**200**), was isolated from the marine-derived endophytic fungus *Paecilomyces variotii* EN-291. Both substances showed weak cytotoxic activity against NCI-H460 tumor cells (IC_50_ 69.3 and 55.9 μmol/L, respectively) [134].

**Figure 54:**
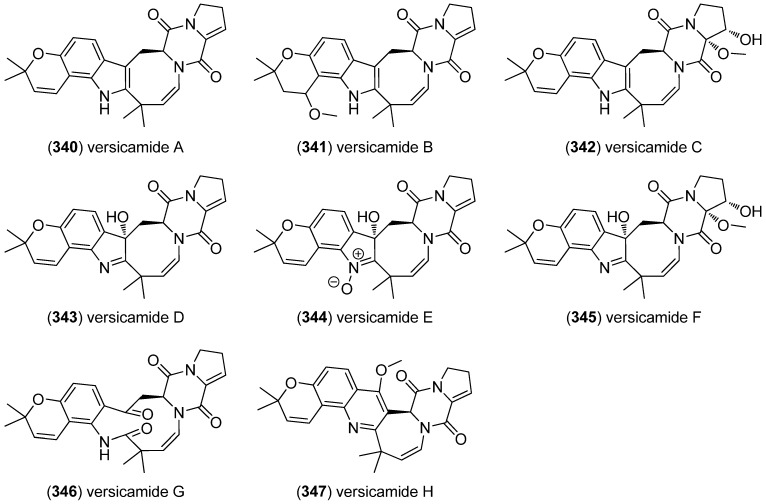
Versicamides A–H.

From *A. versicolor* HDN08-60, eight prenylated indole diketopiperazines, versicamides A–H (**340**–**347**), were obtained, all being structurally related to a singly prenylated Trp-Pro diketopiperazine (Figure 54). The cytotoxicities of versicamides A–H were tested against the HeLa, HCT-116, HL-60, and K562 cell lines, but only versicamide H (**347**) showed moderate cytotoxic activity (IC_50_ 19.4, 17.7, 8.7 and 22.4 μM, respectively) and PTK (protein tyrosine kinase) inhibitory activities [150].

**Figure 55:**
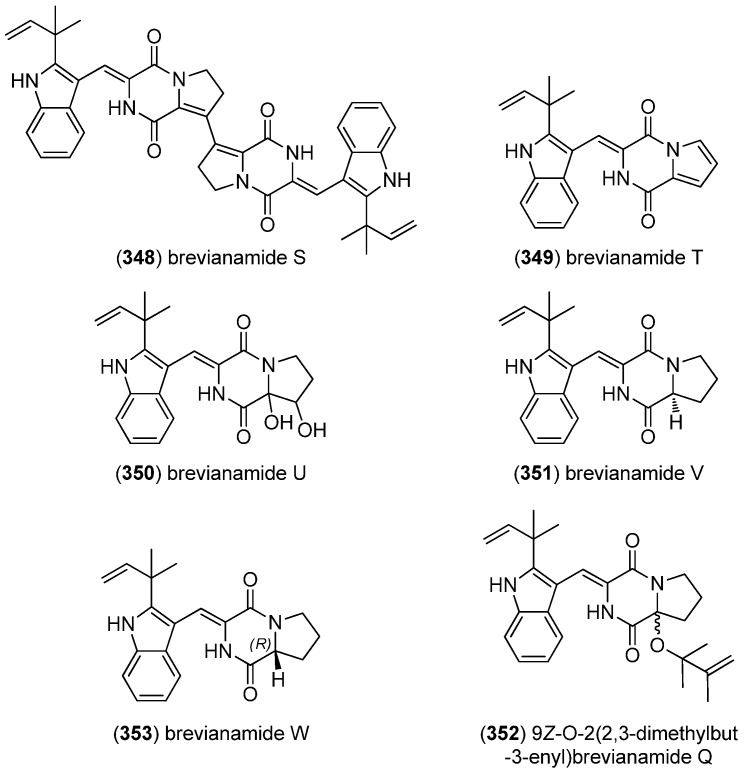
Brevianamides.

Brevianamides S–V (**348**–**351**) and 9*Z*-*O*-2(2,3-dimethylbut-3-enyl)brevianamide Q (**352**) were obtained from strains of *Aspergillus versicolor*, isolated from sediment collected from the Bohai Sea and brown alga *Sargassum thunbergii*, respectively (Figure 55). Brevianamide S (**348**) displayed selective antibacterial activity against Bacille Calmette-Guérin (BCG) which was used as a live attenuated vaccine against tuberculosis [151,152]. Furthermore, brevianamide W (**353**) was isolated from *A. versicolor* CXCTD-06-6a. It showed moderate radical scavenging activity towards DPPH, but no cytotoxic effects [153].

**Figure 56:**
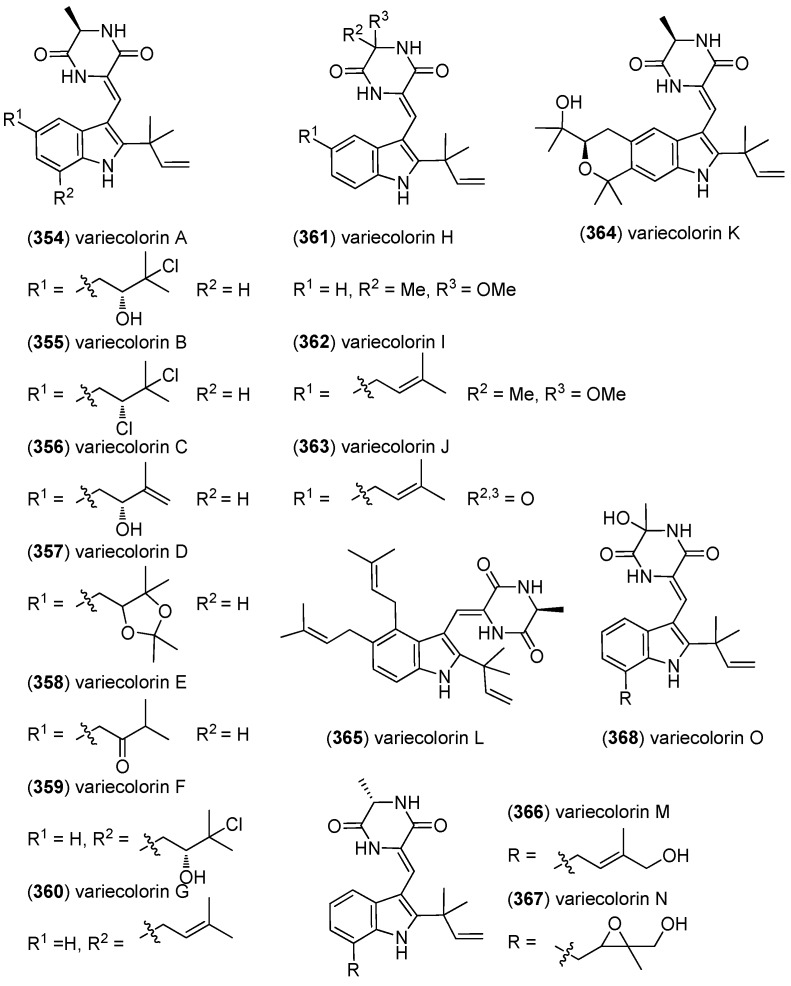
Variecolorins M–O.

The halotolerant fungus *Aspergillus variecolor* was the source of variecolorins A–L (**354**–**365**) (Figure 56) [154]. In contrast, the structurally related variecolorins M–O (**366**–**368**) were isolated from a deep-ocean sediment-derived fungus, *Penicillium griseofulvum* [155]. Variecolorin G (**360**) exhibited moderate lethal activity against brine shrimp (LC_50_ 42.6 μg/mL) [156]. Variecolorins A–K (**354**–**364**) and M–O (**366**–**368**) displayed weak DPPH radical scavenging activity, variecolorins A–O (**354**–**368**) did not show cytotoxic effects against the P388, HL-60, BEL-7402 and A549 cell lines (IC_50_ > 50 μM) [154,155].

**Figure 57:**
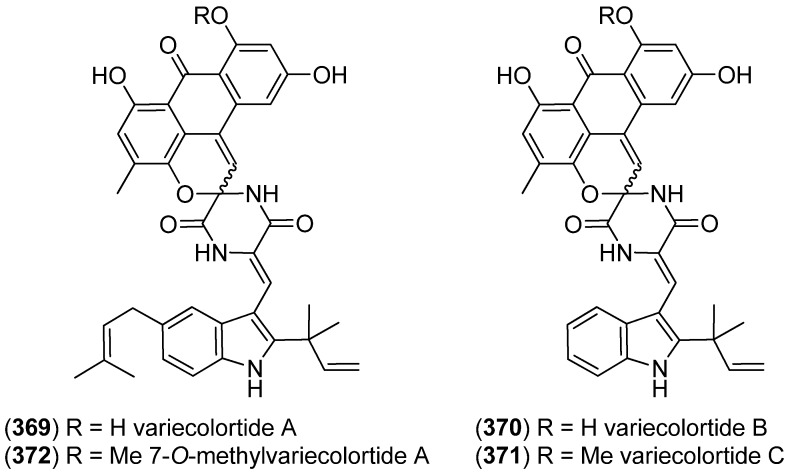
Variecolortides.

Variecolortides A–C (**369**–**371**) were isolated from *Aspergillus variecolor* B-17, obtained from sediments of the Mongolian Jilantai Salt Field, China (Figure 57). They displayed weak cytotoxicity towards the K562 human leukemia cell line (IC_50_ 61, 69 and 71 μM) and exhibited weak DPPH-radical scavenging activities [157]. 7-*O*-Methylvariecolortide A (**372**) was isolated from the mangrove-derived *Eurotium rubrum* [158]. Variecolortides also showed caspase-3 inhibitory activity [159].

**Figure 58:**
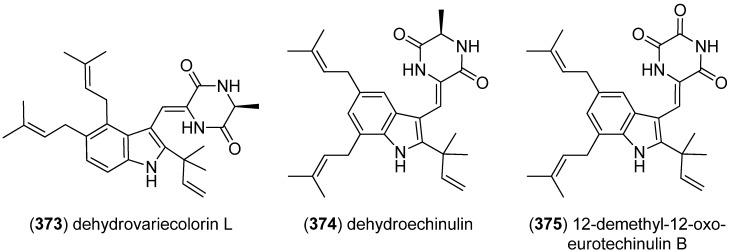
Dehydrovariecolorin L, dehydroechinulin and 12-demethyl-12-oxo-eurotechinulin B.

Examination of the fungal strain *Eurotium rubrum*, an endophytic fungus isolated from the mangrove plant *Hibiscus tiliaceus*, resulted in the isolation of two dioxopiperazine derivatives, dehydrovariecolorin L (**373**) and dehydroechinulin (**374**) (Figure 58). They neither showed radical scavenging nor cytotoxic activity towards the P388, HL-60 and A549 cell lines [160]. 12-Demethyl-12-oxo-eurotechinulin B (**375**) was isolated from the same organism and showed cytotoxic activity against the SMMC-7721 tumor cell line (IC_50_ 30 μg/mL) [161].

**Figure 59:**
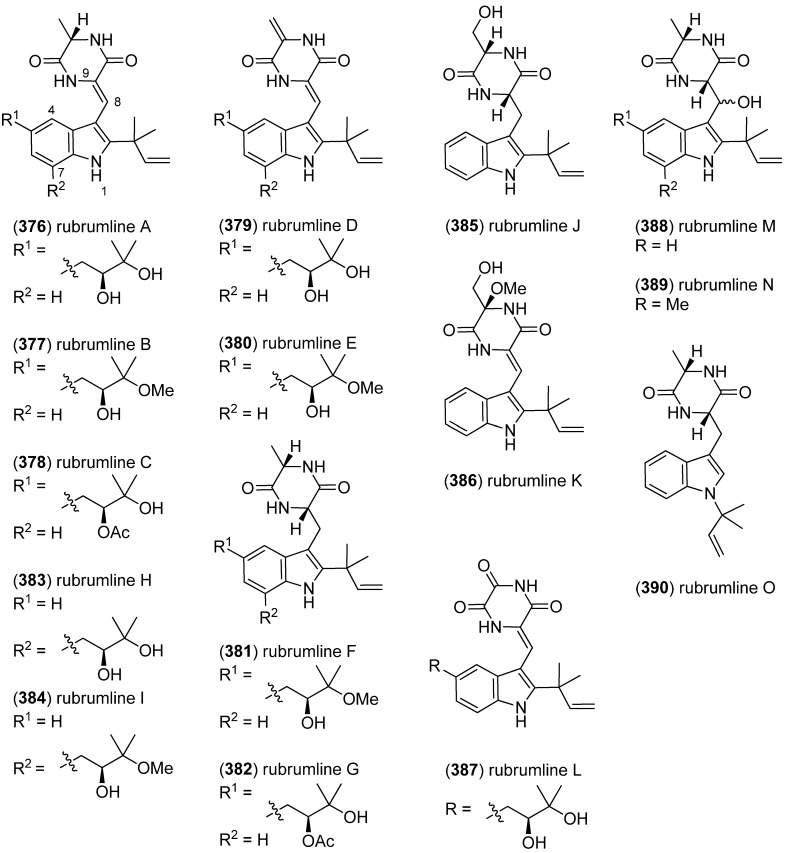
Rubrumlines A–O.

Further investigation of *Eurotium rubrum* revealed fifteen new prenylated indole diketopiperazine alkaloids, named rubrumlines A–O (**376**–**390**) (Figure 59). They were tested against the influenza A/WSN/33 virus and showed variable antiviral activities, governed by their substitution pattern and the presence of the Δ^8,9^ double bond [162].

**Figure 60:**
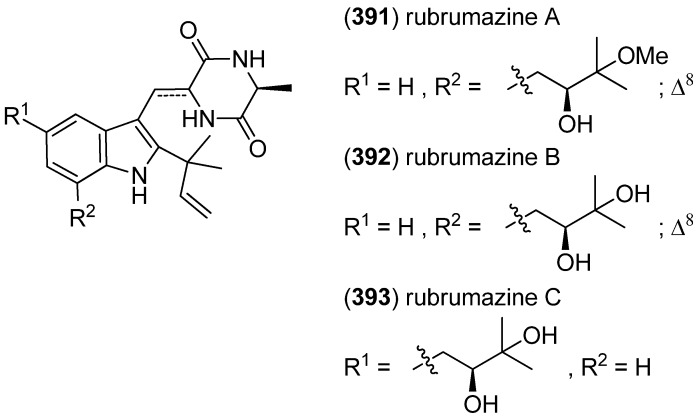
Rubrumazines A–C.

Further isoechinulin type alkaloids, rubrumazines A–C (**391**–**393**), have been obtained from *Eurotium rubrum* MA-150 (Figure 60). Rubrumazine B (**392**) displayed potent activity (LC_50_ 2.43 μM) against brine shrimp (*Artemia salina*), while rubrumazine A (**391**) and C (**393**) showed only modest activity (LC_50_ 29.8 and 16.5 μM, respectively) in the same assay. Rubrumazines did not show activity in antibacterial screening [163].

**Figure 61:**
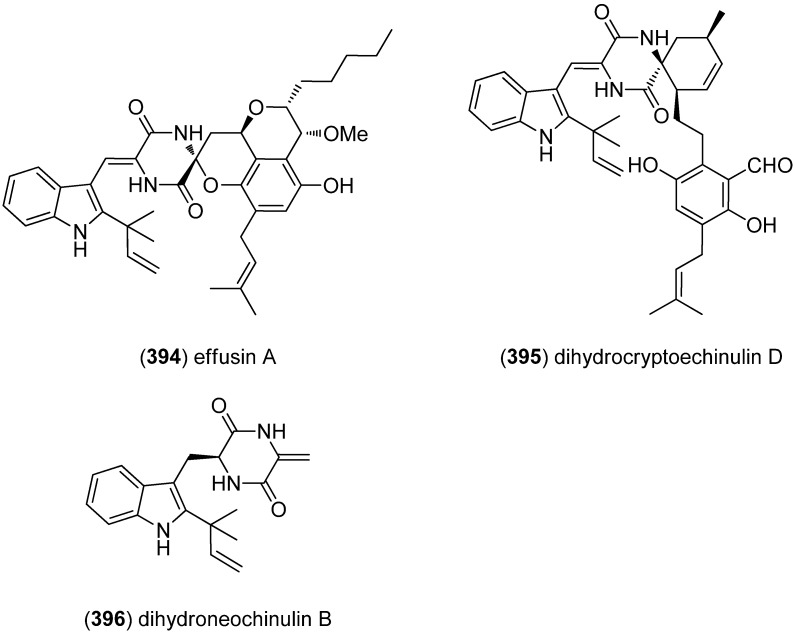
Effusin A, dihydrocryptoechinulin D and dihydroneochinulin B.

Effusin A (**394**) and dihydrocryptoechinulin D (**395**) were isolated from a mangrove-derived fungus, *Aspergillus effuses* H1-1 (Figure 61). Dihydrocryptoechinulin D (**395**) showed cytotoxicity towards P388 and HL-60 cells lines and inhibitory activity on topoisomerase I [164]. Dihydroneochinulin B (**396**) was also isolated from *A. effuses* H1-1 and showed weak cytotoxicity against BEL-7402 and A549 tumor cell lines (IC_50_ 55.1 and 30.5 μM) [165].

**Figure 62:**
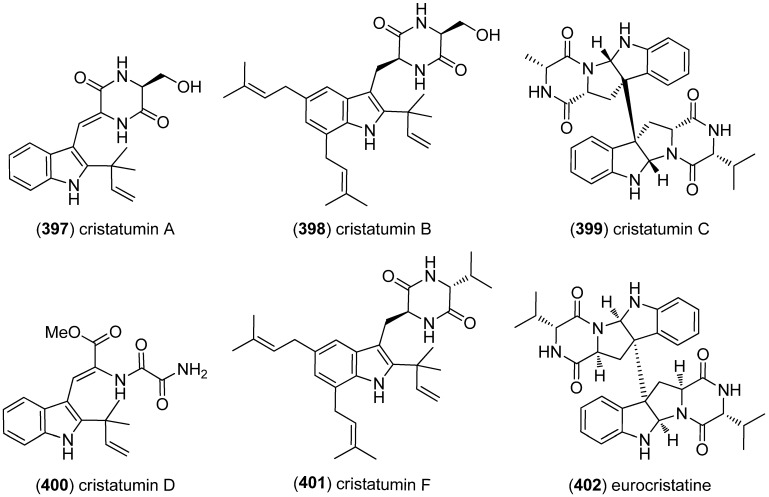
Cristatumins A–F and eurocristatine.

Cristatumins A–D (**397**–**400**) [156] and F (**401**) [166] were identified from the culture extract of *Eurotium cristatum* EN-220, an endophytic fungus isolated from the marine alga *Sargassum thunbergii* (Figure 62). Cristatumin A (**397**) exhibited antibacterial activity against *Escherichia coli* (MIC—minimum inhibitory concentration—64.8 μg/mL), while cristatumin B (**398**) showed moderate activity against brine shrimp (LC_50_ 74.4 μg/mL) [156]. Cristatumin F (**401**) exhibits a valine unit in its diketopiperazine structure, which is unprecedented. It also shows modest radical scavenging activity towards DPPH and a marginal cell proliferation inhibition [166]. Eurocristatine (**402**) was also isolated from *E. cristatum* and did not show any appreciable cytotoxic, antibacterial or antifungal activity [167].

**Figure 63:**
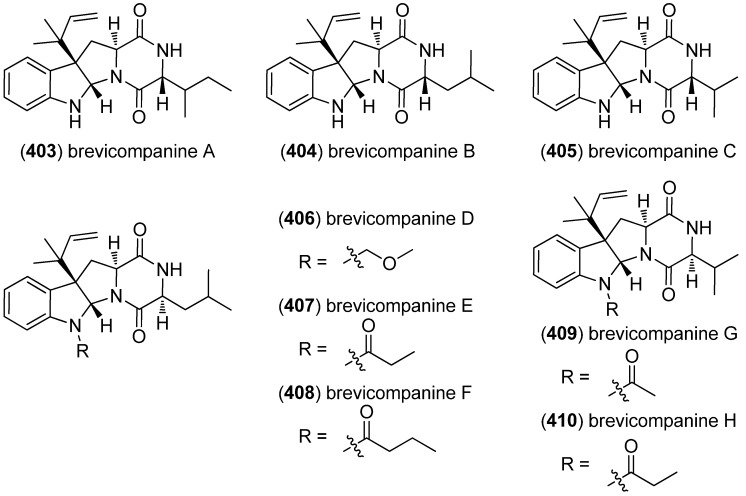
Brevicompanines A–H.

The diketopiperazine alkaloids brevicompanines A (**403**), B (**404**)[168,169], C (**405**) [170] and D–H (**406**–**410**) [171] have been isolated from the fungus *Penicillium brevicompactum*, as well as *Aspergillus janus*, *Penicillium brevi-compactum* Dierckx and *Penicillium* sp. F1, respectively (Figure 63). Brevicompanine B (**404**) showed antiplasmodial activity against the malaria parasite *Plasmodium falciparum* 3D7 (IC_50_ 35 mg/mL, compound precipitated in the test media), but no antifungal or antibacterial effects [168]. Brevicompanines A–C (**403**–**404**) were found to be plant growth regulators [169,170], whereas brevicompanines D–H (**406**–**410**) showed inhibitory effects on lipopolysaccharide-induced inflammation in BV2 microglial cell lines [171].

**Figure 64:**
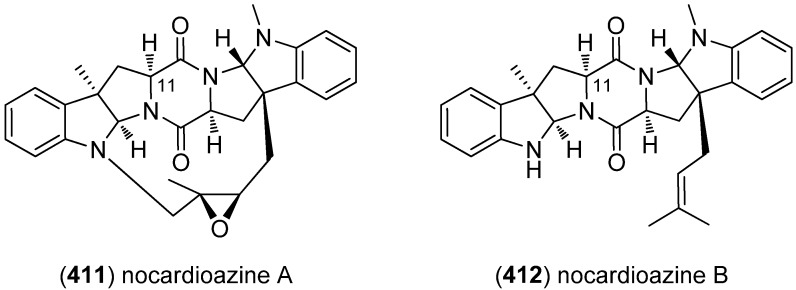
Nocardioazines A and B.

Nocardioazines A (**411**) and B (**412**) have been isolated from an Australian marine sediment-derived bacterium, *Nocardiopsis* sp. (CMB-M0232) (Figure 64). Nocardioazine A (**411**) was found to be an effective and noncytotoxic inhibitor of the multidrug resistance factor P-glycoprotein and is able to reverse doxorubicin resistance in MDR (multi-drug resistant) SW620 Ad300 cells [172].

**Figure 65:**
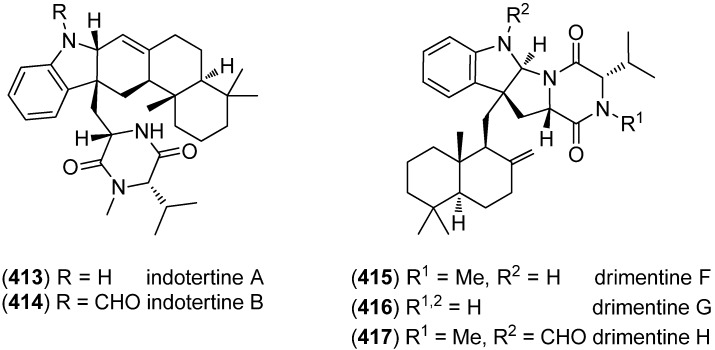
Indotertines A and B and drimentines F–H.

The drimane-substituted tryptophan-derived diketopiperazines indotertines A (**413**) and B (**414**) and drimentines F–H (**415**–**417**) were isolated from the marine sediment-derived actinomycete *Streptomyces* sp. CHQ-64 (Figure 65). Drimentine G (**416**) exhibited cytotoxic activities against HCT-8, Bel-7402, A549, and A2780 cell lines (IC_50_ 2.81, 1.38, 1.01, and 2.54 μM, respectively) together with a weak topoisomerase I inhibitory activity. Indotertine B (**414**) showed cytotoxic activities against the two human tumor cell lines HCT-8 and A549 (IC_50_ 6.96 and 4.88 μM, respectively) [173,174].

**Figure 66:**
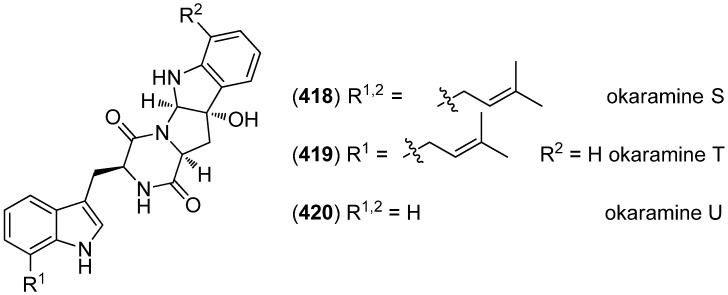
Okaramines S–U.

Okaramines S–U (**418**–**420**) were produced by *Aspergillus taichungensis* ZHN-7-07, isolated from the rhizosphere soil of the mangrove plant *Acrostichum aureum* (Figure 66). Okaramine S (**418**) showed cytotoxic activity against HL-60 and K562 cell lines (IC_50_ 0.78 and 22.4 μM, respectively), but none of them exhibited antibiotic activities [175].

**Figure 67:**
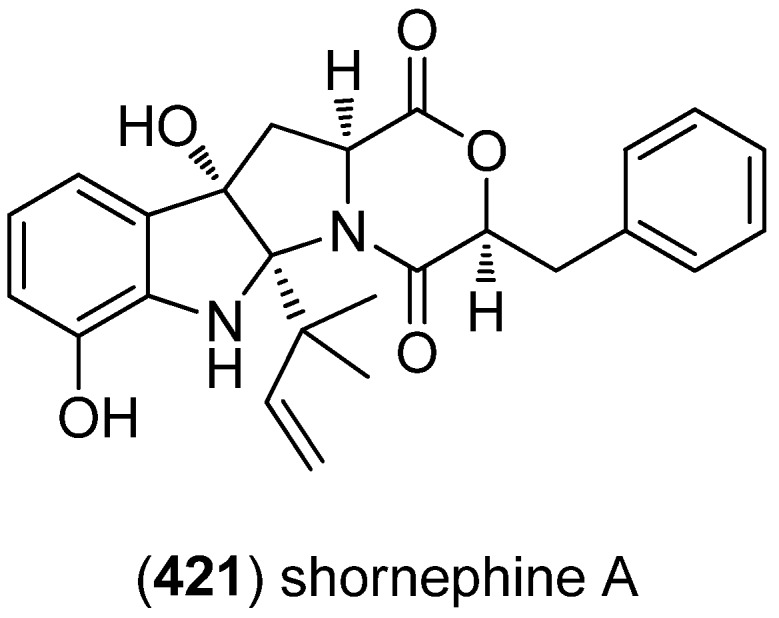
Shornephine A.

The diketomorpholine shornephine A (**421**) was isolated from an Australian marine sediment-derived *Aspergillus* sp. (CMB-M081F) and identified as a noncytotoxic inhibitor of P-glycoprotein-mediated drug efflux in human MDR cancer cells (Figure 67) [176].

**Figure 68:**
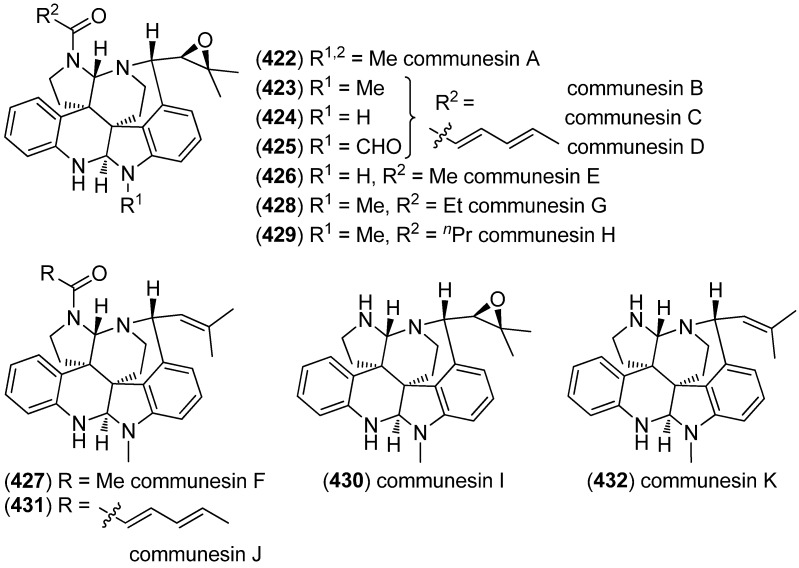
Communesins A–K.

The communesins are a class of cytotoxic and insecticidal marine indole alkaloids [177,178,179]. The first representatives, communesin A (**422**) and B (**423**), have been isolated in 1993 from the mycelium of a strain of *Penicillium* sp. found on the marine alga *Enteromorpha intestinalis* and displayed cytotoxic activity against leukemia cell line P388 (Figure 68) [177]. The extract of *Penicillium* sp*.*, derived from the Mediterranean sponge *Axinella verrucosa*, yielded new derivatives communesins C (**424**) and D (**425**), which exhibit moderate antiproliferative activity against leukemia cell lines U-937, THP-1, NAMALWA, L-428, MOLT-3, and SUP-B15 [179]. Communesins D (**425**), E (**426**) and F (**427**) (published as communesins C, D and E, communesin C turned out to be identical to communesin D published by Jadulco *et al.* [179], communesins E and F being new congeners) were isolated from the Japanese *Penicillium expansum* Link MK-57, together with communesins A and B. All of them showed insecticidal activity against silkworm larvae [178]. Communesins G (**428**) and H (**429**) have been isolated from the psychrotolerant fungus *Penicillium rivulum* Frisvad [180]. Studies of the biosynthetic pathway of the communesin alkaloids led to the identification of communesins I–K (**430**–**432**) and confirmed aurantioclavine as a biosynthetic precursor [181].

**Figure 69:**
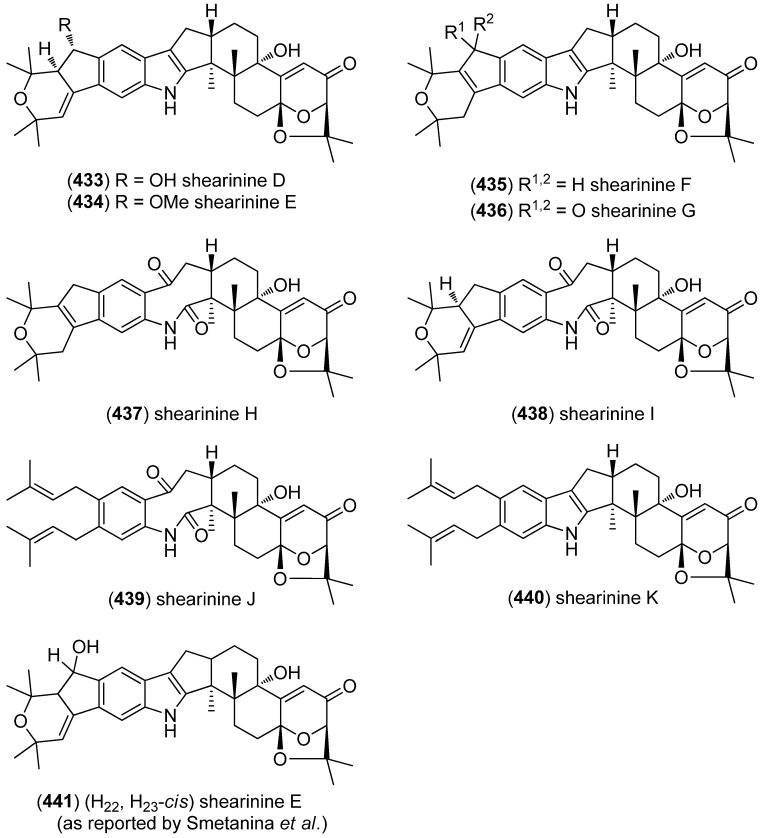
Shearinines D–K (nomenclature according to Xu *et al.*).

Since the shearinines A–C, the first representatives of their class, have been isolated from the sclerotioid ascostromata of *Eupenicillium shearii* [182]. examination of the endophytic fungus *Penicillium* sp., isolated from the mangrove plant *Aegiceras corniculatum*, led to the discovery of marine-derived shearinines D–K (**433**–**440**) (Figure 69) [183]. Simultaneously, three shearinines, named D, E (**441**), and F were isolated by another research group from the marine-derived strain of the fungus *Penicillium janthinellum* Biourge [184]. Shearinine D as published by Smetanina *et al.* was identical to shearinine D reported by Xu *et al.* [183], whereas shearinine F published by Smetanina *et al.* turned out to be identical to shearinine K (**440**) published by Xu *et al.* [183,185]. Shearinines A, D, and E were found to induce apoptosis in the human leukemia cell line HL-60, shearinine E also inhibits EGF-induced malignant transformation of JB6 P^+^ Cl 41 cells [184]. Shearinines A–C show insecticidal activity against *Helicoverpa zea*, *Carpophilus hemipterus* and *Spodoptera frugiperda* [182,186], whereas shearinines D, E and G exhibit inhibitory activity on large-conductance calcium-activated potassium channels [183]. Moreover, shearinines D and E were found to inhibit *Candida albicans* biofilm formation [187]. The biosynthesis of the shearinines H–J involves the cleavage of the 2,3-double bond of the indole ring, a phenomenon well known from tryptophan catabolism.

**Figure 70:**
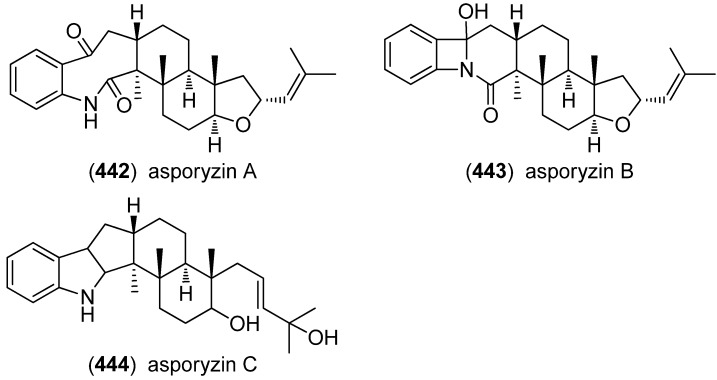
Asporyzins A–C.

Asporyzins A–C (**442**–**444**) were obtained from an endophytic fungus *Aspergillus oryzae*, isolated from the marine red alga *Heterosiphonia japonica* and showed low acetylcholineesterase (AChE) modulating activity (Figure 70) [188].

**Figure 71:**
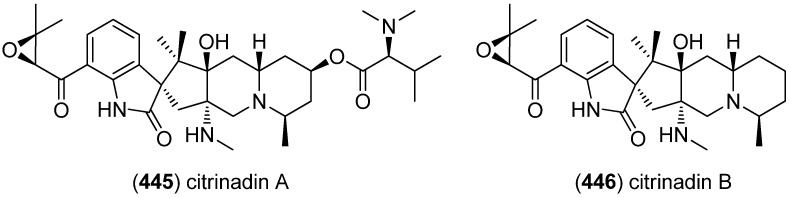
Citrinadins A and B.

The spirocyclic citrinadins A (**445**) [189] and B (**446**) were isolated from *Penicillium citrinum*, obtained from a marine red alga and their absolute configuration was elucidated via ROESY correlations, electronic circular dichroism (ECD), and vibrational circular dichroism (VCD) (Figure 71). Citrinadin B (**446**) showed cytotoxic acitivity against murine leukemia L1210 cells (IC_50_ 10 μg/mL) [190].

**Figure 72:**
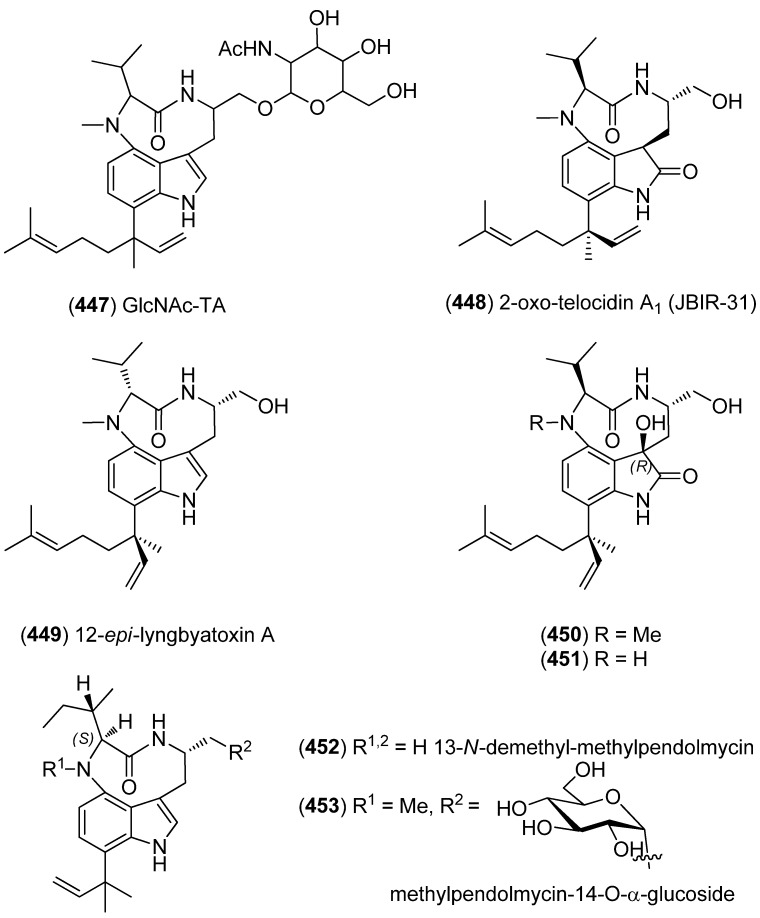
Teleocidin and pendolmycin analogs.

The teleocidin analog 14-*O*-(*N*-acetylglucosaminyl) teleocidin A (GlcNAc-TA) (**447**) was isolated from *Streptomyces* sp. MM216-87F4 and was shown to affect the release of the neurotransmitter substance P from Dorsal Root Ganglia (DRG) neurons via protein kinase C (PKC) pathway (Figure 72) [191]. JBIR-31 (**448**) was isolated from *Streptomyces* sp. NBRC 105896, obtained from the marine sponge *Haliclona* sp. (Tateyama, Japan) and exhibited cytotoxicity towards HeLa and ACC-MESO-1 cell lines (IC_50_ 49 and 88 μM, respectively) [192]. Extraction of *Moorea producens*, collected from Hawaii, afforded lyngbyatoxin derivatives, 12-*epi*-lyngbyatoxin A (**449**), 2-oxo-3(*R*)-hydroxy-lyngbyatoxin A (**450**) and 2-oxo-3(*R*)-hydroxy-13-*N*-demethyl-lyngbyatoxin A (**451**) [193,194].

Indolactam alkaloids, 13-*N*-demethyl-methylpendolmycin (**452**) and methylpendolmycin-14-*O*-α-glucoside (**453**) were isolated from the actinomycete *Marinactinospora thermotolerans* SCSIO 00652. They did not exhibit cytotoxic activities (IC_50_ > 50 μM), but showed antiplasmodial activities against *Plasmodium falciparum* strains 3D7 and Dd2 (IC_50_ 1.92–36.03 μM) [195].

**Figure 73:**
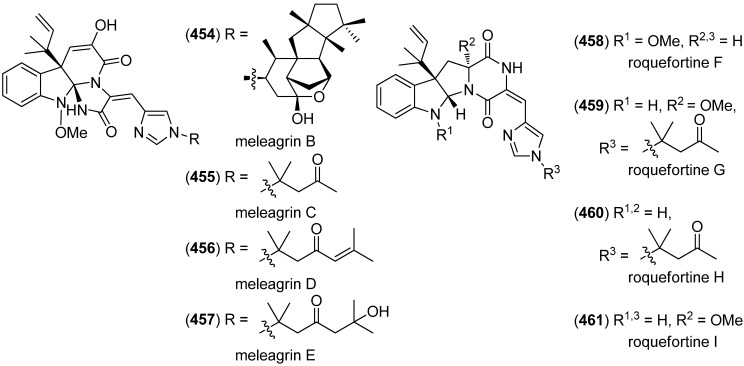
Maleagrins B–E and roquefortines F–I.

Meleagrins B–E (**454**–**457**) and diketopiperazines, roquefortines F–I (**458**–**461**), have been isolated from the deep-sea derived fungus *Penicillium* sp. (Figure 73). Meleagrin B (**454**) showed cytotoxicity against HL-60, MOLT-4, A549 and BEL-7402 cell lines. Likewise, the structurally less complex Maleagrins D (**456**) and E (**457**) were moderately cytotoxic against A549 cell line (IC_50_ 32.2 and 55.9 μM) [196,197].

**Figure 74:**
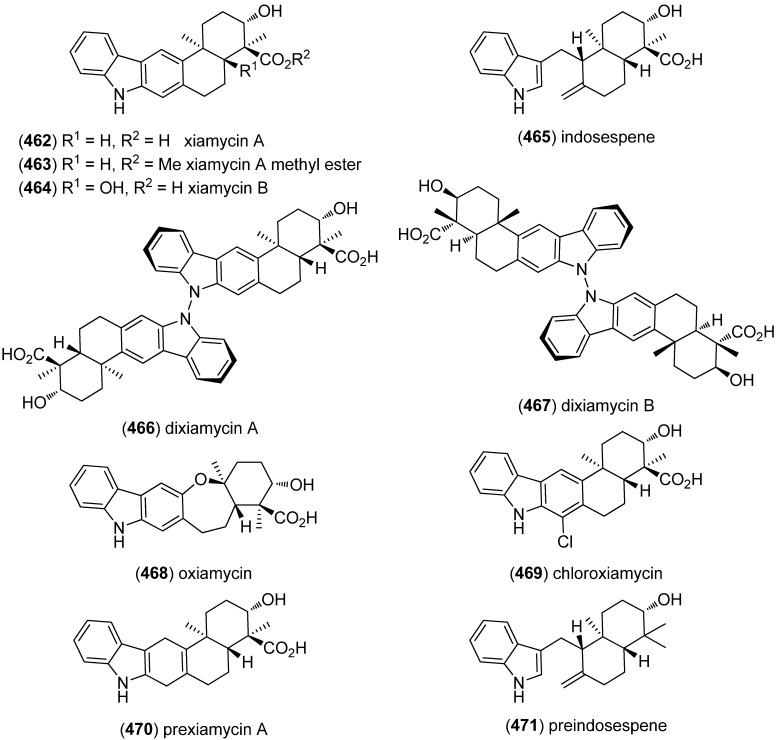
Xiamycin A and its derivatives.

Xiamycin A (**462**) and xiamycin A methyl ester (**463**), pentacyclic carbazole derivatives, were isolated from the endophytic *Streptomyces* sp. GT2002/1503 (from *B. gymnorrhiza*) (Figure 74). Xiamycin A (**462**) was found to be a selective anti-HIV agent by blocking R5-tropic viruses, without having effects on X4-tropic HIV-1 infections [198]. Xiamycin B (**464**) and indosespene (**465**) were isolated from the bacterial mangrove-derived endophyte *Streptomyces* sp. HKI0595 and exhibited strong antibacterial, but no cytotoxic activities against human tumor cell lines [199]. The atropisomeric N-N-linked dimers dixiamycins A (**466**) and B (**467**), oxiamycin (**468**), and chloroxiamycin (**469**) were isolated from a marine-derived actinomycete *Streptomyces* sp. SCSIO 02999. They exhibit antibacterial activity, the two dimeric metabolites being most active [200]. Prexiamycin (**470**) and preindosespene (**471**) were identified as intermediates in the xiamycin biosynthesis [15].

**Figure 75:**
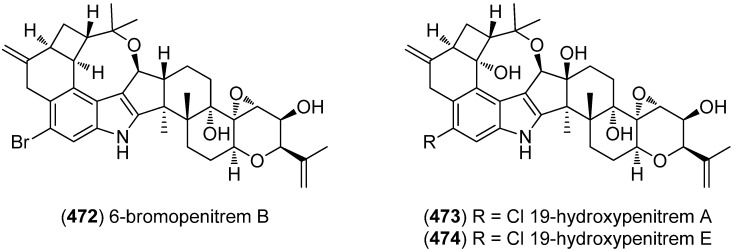
Penitrem derivatives.

**Figure 76:**
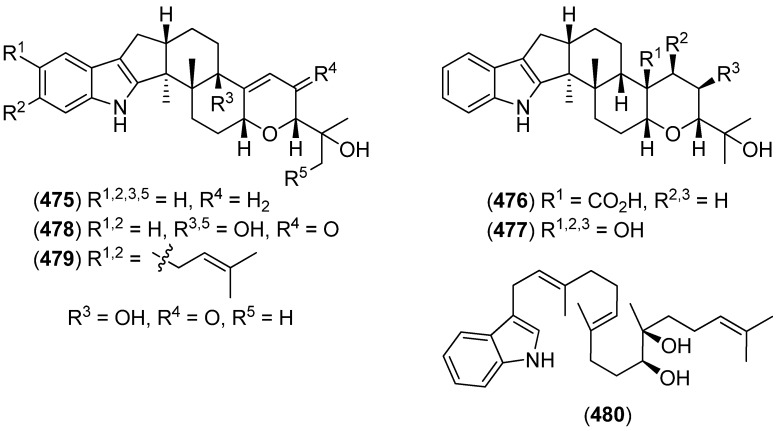
Metabolites from *Penicillium camemberti.*

With 6-bromopenitrem B (**472**), a new penitrem derivative was isolated from the marine-derived fungus *Penicillium commune* isolate GS20 (supplemented with potassium bromide) (Figure 75). It showed significant anti-invasive effects, as well as antiproliferative activity against MCF-7 and MDA-MB-231 tumor cell lines [201]. 19-Hydroxypenitrem A (**473**) and 19-hydroxypenitrem E (**474**) were isolated from the endophytic marine red alga derived fungus *Aspergillus nidulans* EN-330 . Both compounds showed brine shrimp cytotoxicity (LD_50_ 3.2 and 4.6 μM) and 19-hydroxypenitrem A (**473**) exhibited antimicrobial activity towards *Edwardsiella tarda*, *Vibrio anguillarum*, *Escherichia coli* and *Staphylococcus aureus* (MIC 16, 32, 16, and 16 μg/mL, respectively) [202].

(2*S*,4b*R*,6a*S*,12b*S*,12c*S*,14a*S*)-3-Deoxo-4b-deoxypaxilline (**475**), (2*S*,4a*R*,4b*R*,6a*S*,12b*S*,12c*S*,14a*S*)-4a-demethylpaspaline-4a-carboxylic acid (**476**), (2*S*,3*R*,4*R*,4a*S*,4b*R*,6a*S*,12b*S*,12c*S*,14a*S*)-4a-demethyl-paspaline-3,4,4a-triol (**477**), (2*R*,4b*S*,6a*S*,12b*S*,12c*R*,14a*S*)-2′-hydroxypaxilline (**478**), (2*R*,4b*S*,6a*S*,12b*S*,12c*R*,14a*S*)-9,10-diisopentenylpaxilline (**479**) and (6*S*,7*R*,10*E*,14*E*)-16-(1*H*-indol-3-yl)-2,6,10,14-tetramethylhexadeca-2,10,14-triene-6,7-diol (**480**) were isolated from the marine-derived *Penicillium camemberti* OUCMDZ-1492. Some compounds exhibited activity against H_1_N_1_ influenza A virus (Figure 76) [203].

**Figure 77:**
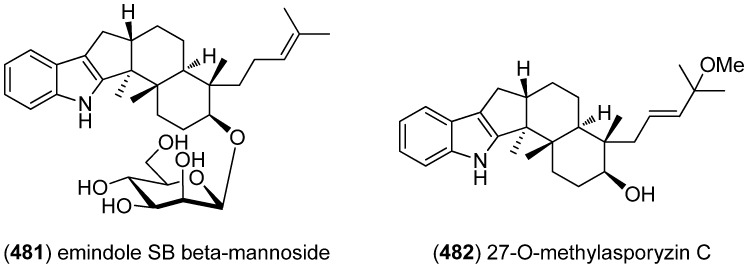
Emindole SB beta-mannoside and 27-*O*-methylasporyzin C.

Emindole SB beta-mannoside (**481**) and 27-*O*-methylasporyzin C (**482**) were isolated from a marine-derived strain of *Dichotomomyces cejpii* (Figure 77)*.* Emindole SB beta-mannoside (**481**) was identified as a CB_2_ antagonist (*K*_i_ 10.6 μM), while 27-*O*-methylasporyzin C (**482**) was found to be a GPR18 (G-protein coupled receptor 18, *N*-arachidonyl glycine receptor) antagonist (IC_50_ 13.4 μM) [204].

#### Ergoline Alkaloids

**Figure 78:**
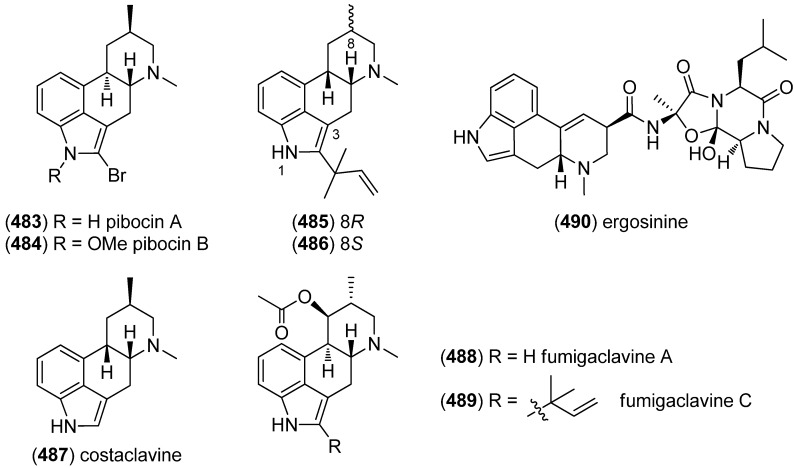
Ergoline alkaloids.

Among the prenylated indole alkaloids, ergot alkaloids are a well-established group of natural products, known for their potent and manifold biological activities. Ergot alkaloids have been isolated from terrestrial sources exclusively until Pibocin A (**483**), the first representative of marine ergoline alkaloids, was obtained from extracts of the Far-Eastern ascidian *Eudistoma* sp. (Figure 78) [205]. Pibocins A (**483**) and B (**484**) [206] were found to show antimicrobial and cytotoxic effects against mouse Ehrlich carcinoma cells [205,206]. 2-(3,3-Dimethylprop-1-ene)-costaclavine (**485**) and 2-(3,3-dimethylprop-1-ene)-*epi*-costaclavine (**486**) were isolated from the marine-derived fungus *Aspergillus fumigatus*, together with known clavine-type alkaloids costaclavine (**487**) and fumigaclavines A (**488**) [207] and C (**489**) [208]. Except of fumigaclavine A, all of them were found to show weak cytotoxicity against the mouse leukemia cell line P388 [209]. Additionally, fumigaclavine C (**489**) was found to induce apoptosis in MCF-7 breast cancer cells [210]. Ergosinine (**490**), which was isolated from the marine mollusc *Pleurobranchus forskalii*, is the first ergot peptide alkaloid (ergopeptine) found in marine life. The authors propose that ergot alkaloids may play a defensive or protective role in mollusks and other marine organisms [211].

### 2.3. Bis- and Trisindoles

Bis- and trisindole alkaloids are biosynthetically derived from two or three indole building blocks. They show diverse biological activities, as antiviral, antitumor, antibacterial and anti-inflammatory activities and are therefore promising chemical leads for drug development [212,213].

**Figure 79:**
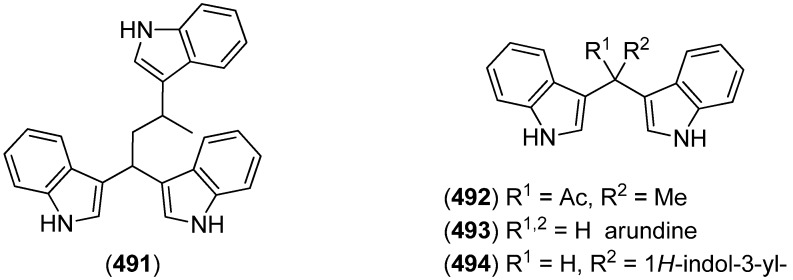
Bis- and trisindoles.

Trisindole alkaloid 1,1,3-tris(3-indolyl)butane (**491**) was isolated from a North Sea bacterium *Vibrio parahaemolyticus* Bio249, together with 3,3-bis(3-indolyl)butane-2-one (**492**), arundine (**493**), and 1,1,1-tris(3-indolyl)methane (**494**), which were isolated from a microorganism for the first time (Figure 79). Antibiotic or antifungal activities could not be evidenced [214].

**Figure 80:**
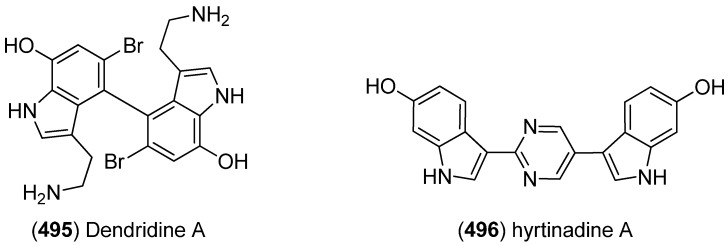
Dendridine A and hyrtinadine A.

Dendridine A (**495**) was isolated from the marine sponge *Dictyodendrilla* sp. and showed antibacterial activity against *Bacillus subtilis* and *Micrococcus* luteus (MIC 8.3 and 4.2 μg/mL) as well as antifungal activity against *Cryptococcus neoformans* (MIC 8.3 μg/mL) and weak cytotoxic effects against L1210 tumor cells (IC_50_ 32.5 μg/mL) (Figure 80) [215]. Hyrtinadine A (**496**) was obtained from an Okinawan marine sponge *Hyrtios* sp. (SS-1127). It displayed cytotoxic activities against murine leukemia L1210 cells and human epidermoid carcinoma KB cells (IC_50_ 1 and 3 μg/mL, respectively) [216].

**Figure 81:**
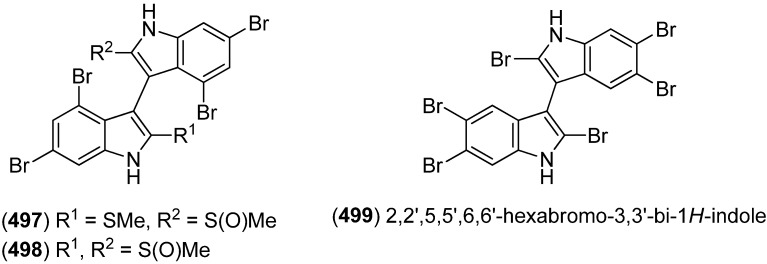
Polybrominated bisindoles.

Polybrominated bisindoles 3,3′-bis(2′-methylsulfinyl-2-methylthio-4,6,4′,6′-tetrabromo)indole (**497**) and 3,3′-bis(4,6-dibromo-2-methylsulfinyl)indole (**498**) were isolated from the Formosan red alga *Laurencia brongniartii* (Figure 81). Bisindoles **497** and **498** displayed cytotoxicity against HT-29 and P388 cell lines, respectively [40]. 2,2′,5,5′,6,6′-Hexabromo-3,3′-bis-1*H*-indole (**499**) was isolated from the marine red alga *Laurencia similis* [44].

**Figure 82:**
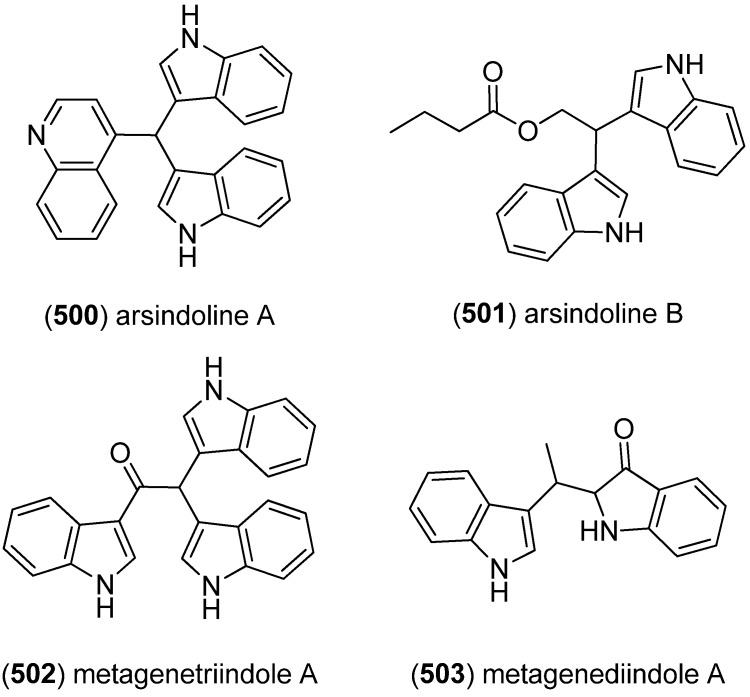
Arsindolines A, B, metagenetriindole A and metagenediindole A.

Arsindolines A (**500**) and B (**501**) were isolated from a marine-derived bacterium strain *Aeromonas* sp. CB101 (Figure 82). Arsindoline B (**501**) showed antitumor activity against cell line A549 (IC_50_ 22.6 μM) [217]. Metagenetriindole A (**502**) and metagenediindole A (**503**) were obtained from a deep-sea sediment derived *Escherichia coli* strain and exhibited moderate cytotoxic activity against CNE2, Bel7402 and HT1080 cancer cell lines (IC_50_ 34.25–50.55 μg/mL) [218].

**Figure 83:**
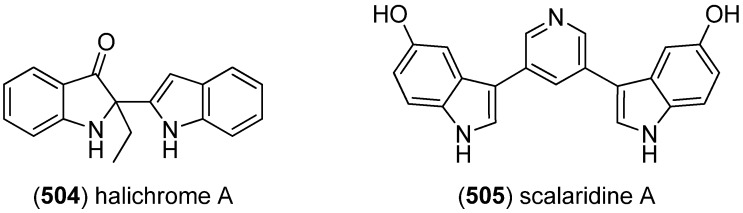
Halichrome A and scalaridine A.

The yellow pigment halichrome A (**504**) was isolated from a metagenomic library derived from the marine sponge *Halichondria okadai* and showed cytotoxicity against B16 melanoma cells (Figure 83) [219]. Scalaridine A (**505**), together with 5-hydroxyindole alkaloids **150** and **151**, was isolated from the marine sponge *Scalarispongia* sp. collected near Dokdo Island. It is the first bisindole alkaloid with a pyridine linker and exhibits cytotoxic activity against human leukemia cells K562 (IC_50_ 39.5 µg/mL) [97].

**Figure 84:**
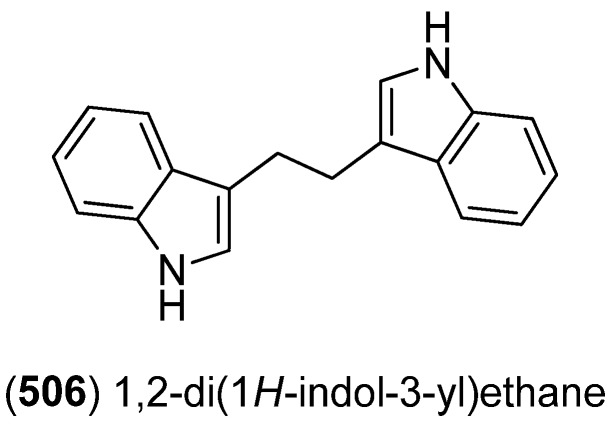
1,2-Di(1*H*-indol-3-yl)ethane.

Bisindolic 1,2-di(1*H*-indol-3-yl)ethane (**506**, Figure 84) was isolated from the marine bacterium *Pantoea agglomerans* P20-14, together with monoindoles **87** and **88** (see Figure 21) [73].

**Figure 85:**
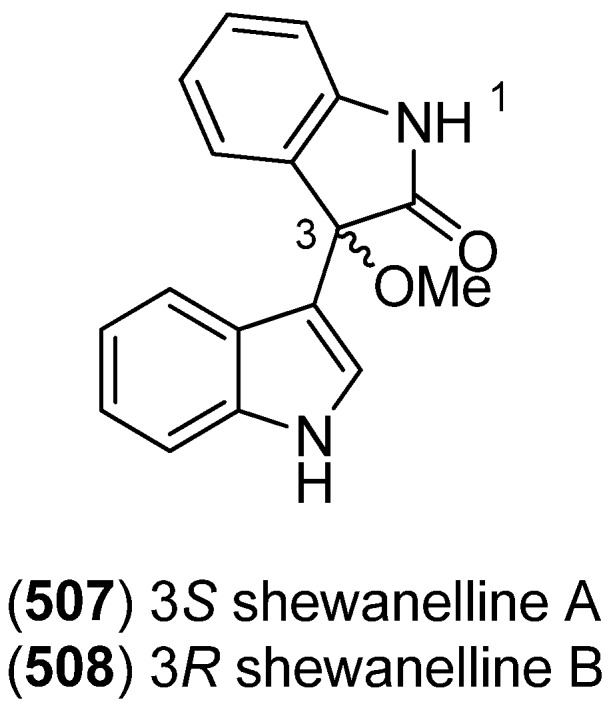
Shewanellines A and B.

Shewanellines A–C (**507**, **508** and **139**) were isolated from the deep-sea bacterium *Shewanella piezotolerans* WP3 (Figure 85). Since shewanelline C does not belong to the bisindoles, it is discussed with the simple indole alkaloids (see Figure 34) [89].

**Figure 86:**
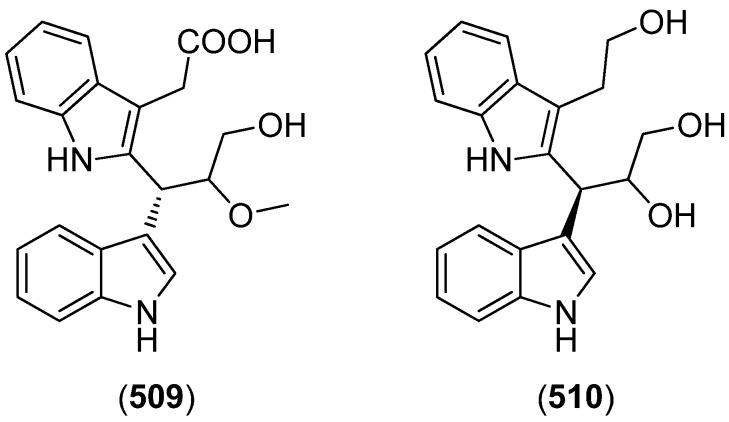
2-(2-(3-Hydroxy-1-(1*H*-indol-3-yl)-2-methoxypropyl)-1*H*-indol-3-yl) acetic acid and 3-(3-(2-hydroxyethyl)-1*H*-indol-2-yl)-3-(1*H*-indol-3-yl)propane-1,2-diol).

2-(2-(3-Hydroxy-1-(1*H*-indol-3-yl)-2-methoxypropyl)-1*H*-indol-3-yl) acetic acid (**509**) and 3-(3-(2-hydroxyethyl)-1*H*-indol-2-yl)-3-(1*H*-indol-3-yl)propane-1,2-diol) (**510**) were isolated from the marine actinomycete *Rubrobacter radiotolerans* (Figure 86). Both showed AchE inhibitory activity but no significant cytotoxic effects [220].

**Figure 87:**
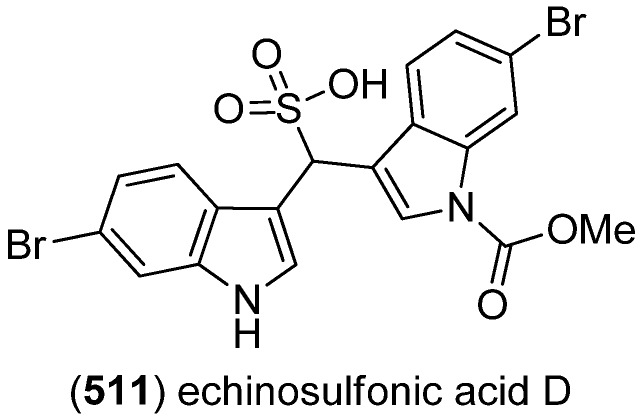
Echinosulfonic acid D.

Echinosulfonic acid D (**511**) was isolated from the New-Caledonian sponge *Psammoclemma* sp. and showed cytotoxic effects towards KB cells (IC_50_ 2 µg/mL) (Figure 87) [221].

**Figure 88:**
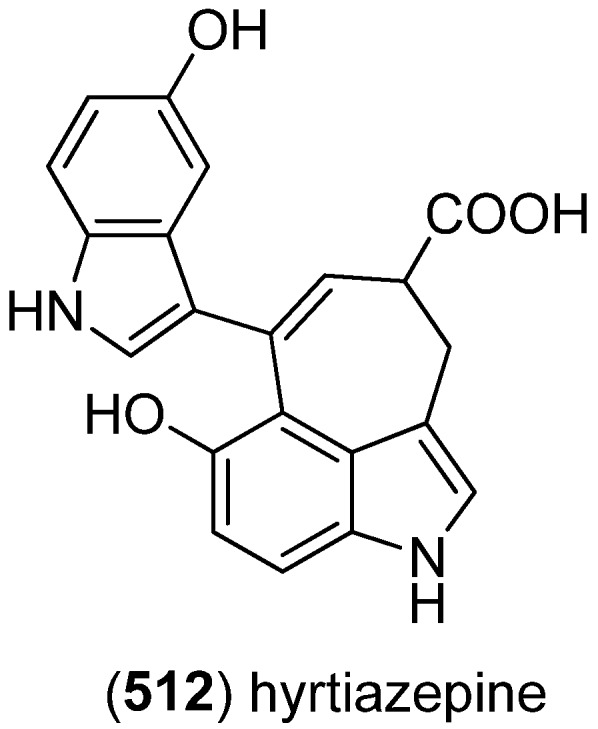
Hyrtiazepine.

Hyrtiazepine (**512**) was isolated from *Hyrtios erectus* (Figure 88) [35].

**Figure 89:**
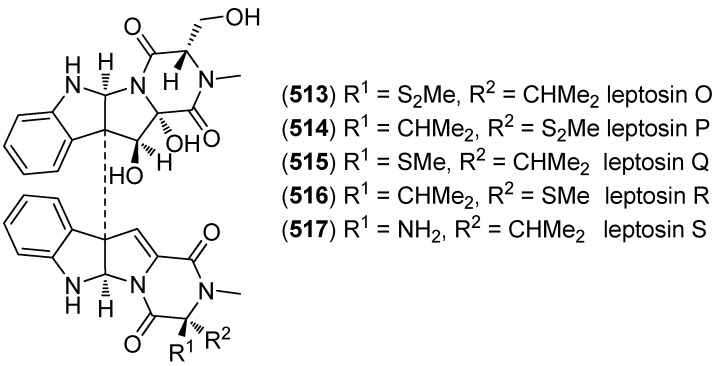
Leptosins O–S.

Leptosins O–S (**513**–**517**) were isolated from a strain of *Leptosphaeria* sp., originated from the marine alga *Sargassum tortile* (Figure 89). Leptosins O (**513**) and P (**514**) exhibited cytotoxic activity against P388 cells [222].

**Figure 90:**
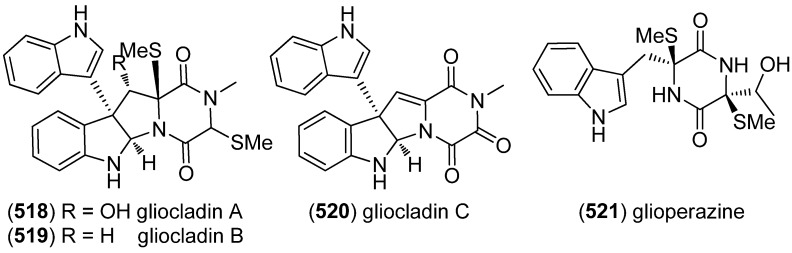
Gliocladins A–C and glioperazine.

Gliocladins A–C (**518**–**520**) and glioperazine (**521**) were isolated from a sea hare-derived strain of *Gliocladium* sp. (Figure 90). Gliocladin C (**520**) displayed cytotoxic activity against P388 cells [223].

**Figure 91:**
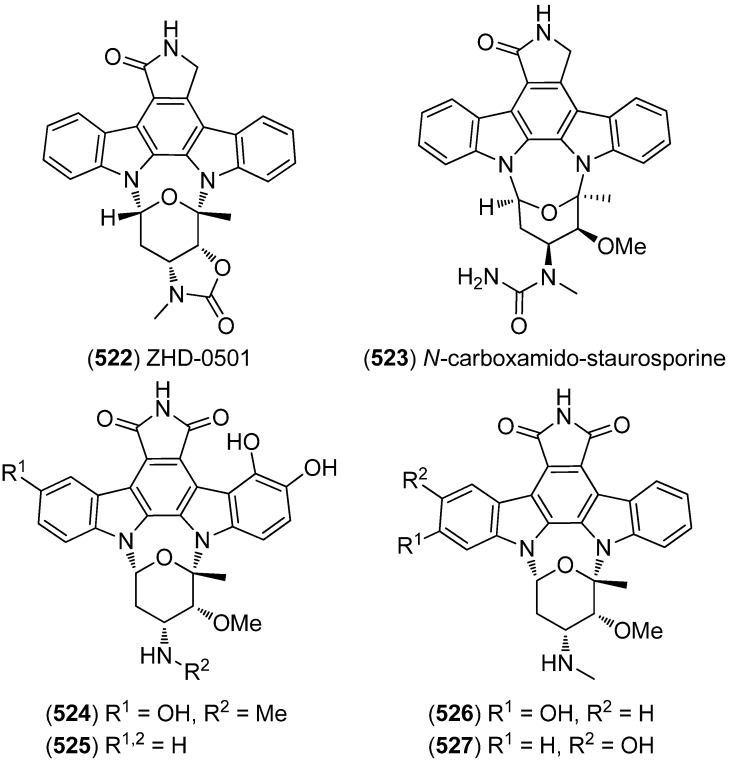
Staurosporine derivatives.

ZHD-0501 (**522**), a staurosporine analog, was isolated from the marine-derived *Actinomadura* sp. 007 and displayed cytotoxic activity towards the cancer cell lines A549, BEL-7402, HL-60, P388 and tsFT210 (Figure 91) [224]. *N*-Carboxamido-staurosporine (**523**) was isolated from the marine-derived *Streptomyces* sp. QD518. It was found to have antibacterial, as well as potent and selective cytotoxic, activity [225]. 7-Oxo-3,8,9-trihydroxystaurosporine (**524**) and 7-oxo-8,9-dihydroxy-4′-*N*-demethylstaurosporine (**525**) were isolated from the marine ascidian *Cystodytes solitus*. As is common for this compound class, both compounds exhibited cytotoxicity towards A549, HT-29 and MDA-MB-231 cell lines (GI_50_ 17.5–90 nM) [226]. 2-Hydroxy-7-oxostaurosporine (**526**) and 3-hydroxy-7-oxostaurosporine (**527**) were isolated from the Brazilian tunicate *Eudistoma vannamei* and showed cytotoxicity against the human cancer cell lines HL-60, MOLT-4, Jurkat, K562, HCT-8, SF-295 and MDA-MB-435 (IC_50_ 10.33–144.47 nM) [227].

**Figure 92:**
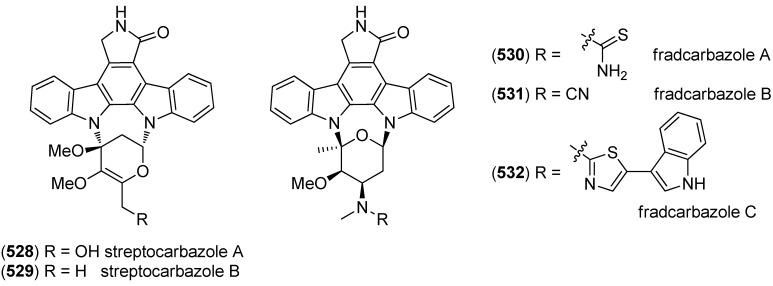
Streptocarbazoles A and B and fradcarbazoles A–C.

Analysis of the marine-derived actinomycetes strain *Streptomyces* sp. FMA led to the discovery of streptocarbazoles A (**528**) and B (**529**) (Figure 92). Streptocarbazole A (**528**) has cytotoxic activity towards L-60, A549, P388 and HeLa cell lines (IC_50_ 1.4, 5.0, 18.9, and 34.5 μM, respectively) and arrests the cell cycle of HeLa cells in the G_2_/M phase at a concentration of 10 μM [228]. Fradcarbazoles A–C (**530**–**532**) were obtained from a mutant strain of the marine-derived actinomycete *Streptomyces fradiae* 007M135. They exhibited strong cytotoxic effects towards HL-60, K562, A549 and BEL-7402 cell lines (IC_50_ 0.001–4.58 μM) and were found to be potent kinase PKC-α inhibitors (IC_50_ 0.16–4.27 μM) [229].

**Figure 93:**
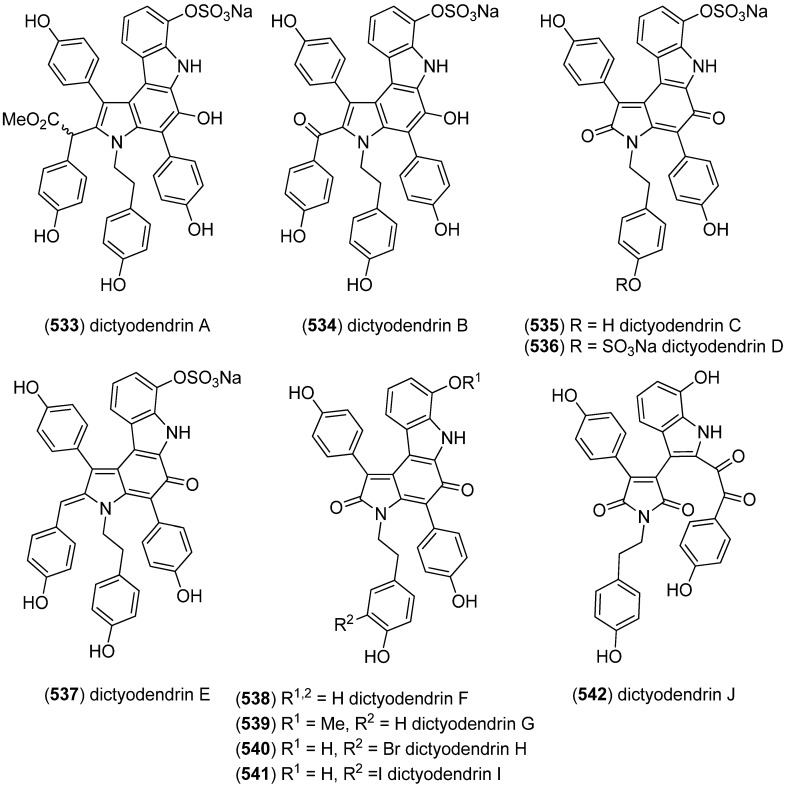
Dictyodendrins A–J.

Dictyodendrins A–E (**533**–**537**) were obtained from the Japanese marine sponge *Dictyodendrilla verongiformis* and exhibited telomerase inhibitory activity (Figure 93) [230]. Dictyodendrins F–J (**538**–**542**) were isolated from a southern Australian marine sponge *Ianthella* sp. (CMB-01245). Dictyodendrins F (**538**) and H–J (**540**–**542**) exhibited protease β-secretase (BACE) inhibitory activity and dictyodendrins F–I (**538**–**541**) have cytotoxic activity against human colon cancer cell line SW620 and the P-glycoprotein over-expressing SW620 Ad300 [231].

**Figure 94:**
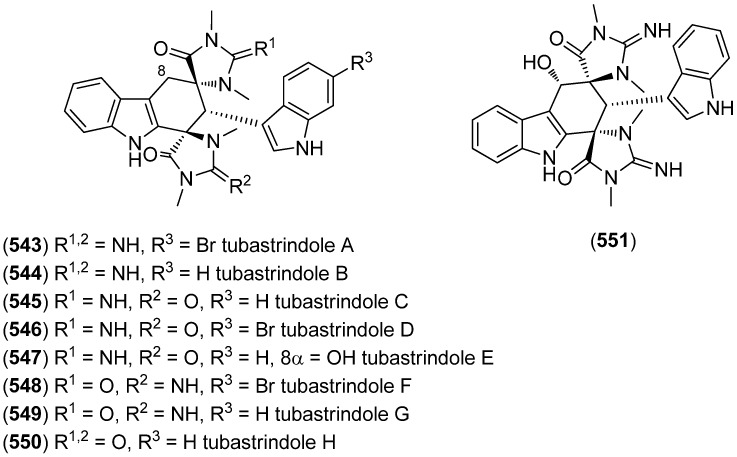
Tubastrindoles A–H.

Tubastrindoles A–C (**543**–**545**) were isolated from a stony coral, *Tubastraea* sp. (Figure 94) [232]. Tubastrindole B (**544**) turned out to be a potent and selective α1 GlyR (glycine-gated chloride channel receptor) antagonist (IC_50_ 25.9 μM) [233]. Tubastrindoles D–H (**546**–**550**) were obtained from *Tubastraea aurea* (Odomari, Kagoshima, Japan) [234]. Bisindole alkaloid **551** was also isolated from *Tubastraea* sp. and exhibited antiplasmodial activity [235].

**Figure 95:**
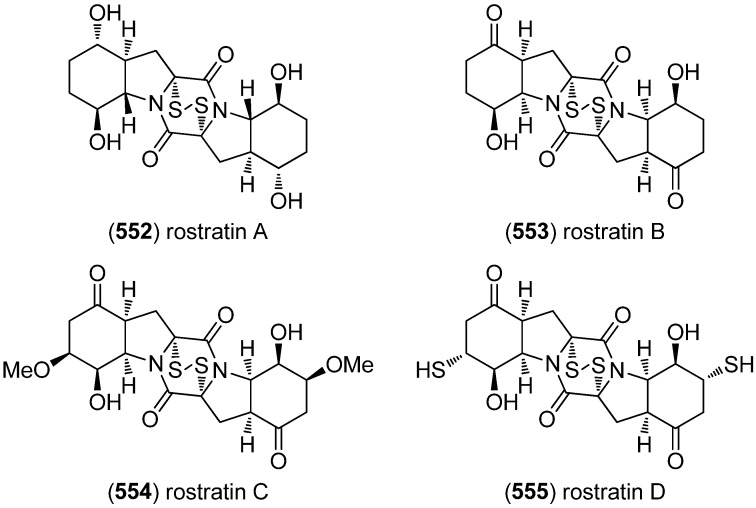
Rostratins A–D.

Rostratins A–D (**552**–**555**) were obtained from a marine-derived strain of the fungus *Exserohilum rostratum* (Drechsler) and exhibited cytotoxic activity against human colon carcinoma HCT-116 (IC_50_ 8.5, 1.9, 0.76 and 16.5 μg/mL, respectively) (Figure 95) [236].

**Figure 96:**
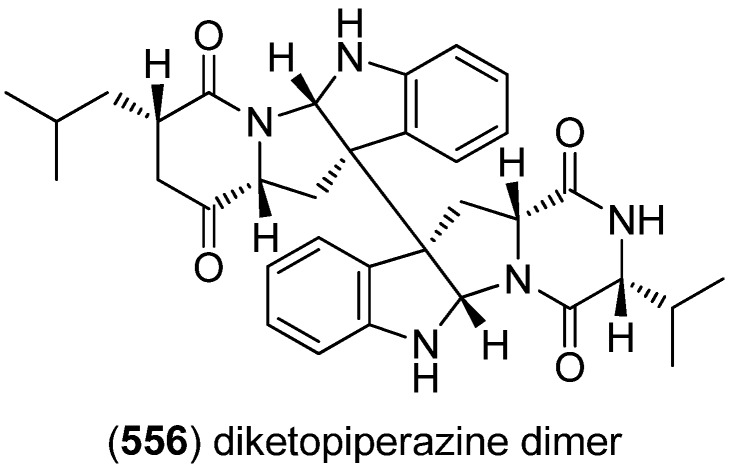
Diketopiperazine dimer.

**Figure 97:**
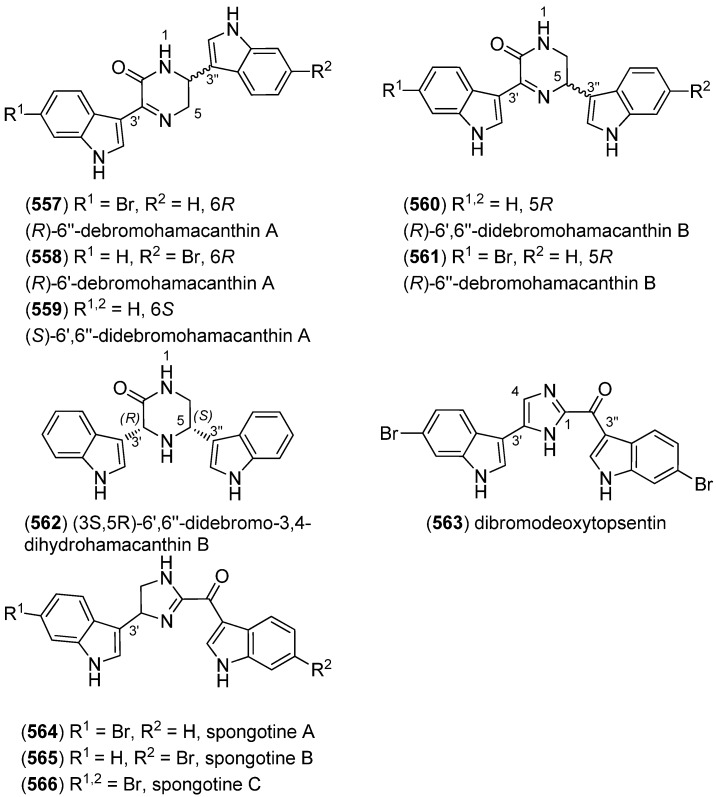
Hamacanthin and topsentin derivatives.

Diketopiperazine dimer **556** was isolated from a marine-derived isolate of *Aspergillus niger* (Figure 96) [237].

(*R*)-6″-Debromohamacanthin B (**556**) was isolated from the marine sponge *Spongosorites* sp. (**556**) exhibited low inhibitory activity against *S. aureus* sortase A (SrtA) (Figure 97) [238]. Nine new bisindole alkaloids were isolated from the same organism, five of them belonging to the hamacanthin (**557**–**560**, **562**) and four to the topsentin class (**563**–**566**). The structure of spongotine B (**565**) was erroneously reported as (*S*)-6″-debromohamacanthin B in the earlier report of Bao *et al.* [239,240]. They partially exhibited cytotoxic activity against the human tumor cell lines A549, SK-OV-3, SK-MEL-2, XF498 and HCT-15, as well as weak antibacterial activity against several methicillin-resistant strains [239,240].

**Figure 98:**
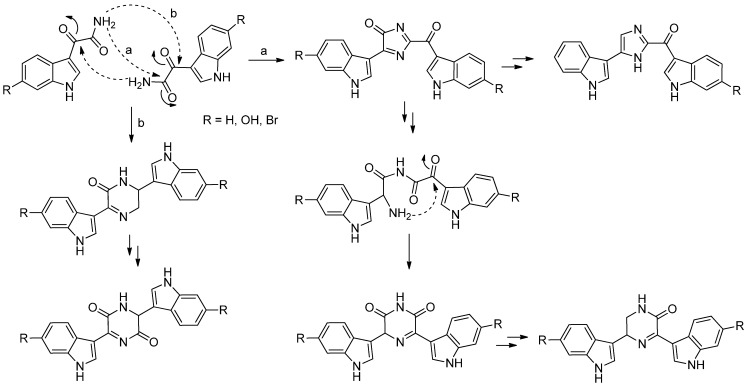
Hypothetical biosynthesis of topsentins and hamacanthins [56].

6″-Debromohamacanthin A (**557**) revealed significant antibacterial activity against Gram-positive and Gram-negative bacteria including MRSA (Methicillin-resistant *Staphylococcus aureus*), as well as antifungal activity [241]. It was also found out to target the VEGFR2 (vascular endothelial growth factor receptor 2)-mediated PI3K/AKT/mTOR signaling pathway and, thus, effectively inhibit angiogenesis [242]. 6″-Debromohamacanthin A (**557**), 6″-debromohamacanthin B (**561**) and spongotine A (**564**) showed antitumor activity against AGS and L1210 cancer cell lines [241]. Figure 98 shows a hypothetical biosynthesis of topsentins and hamacanthins from monomeric indole pyruvic acid derivatives [56].

**Figure 99:**
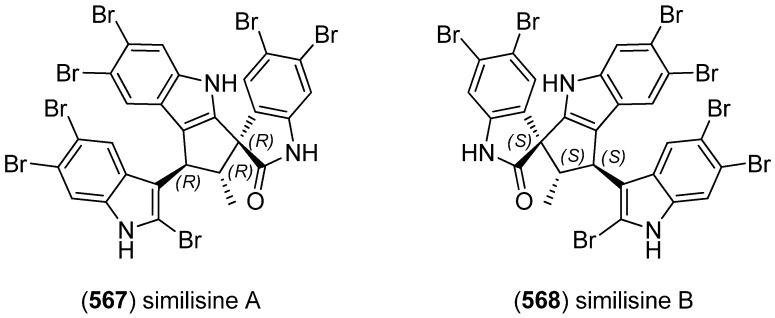
Similisines A and B.

Two unprecedented brominated spiro-trisindole alkaloids, similisines A (**567**) and B (**568**), were isolated from *L. similis* (Figure 99) [243].

**Figure 100:**
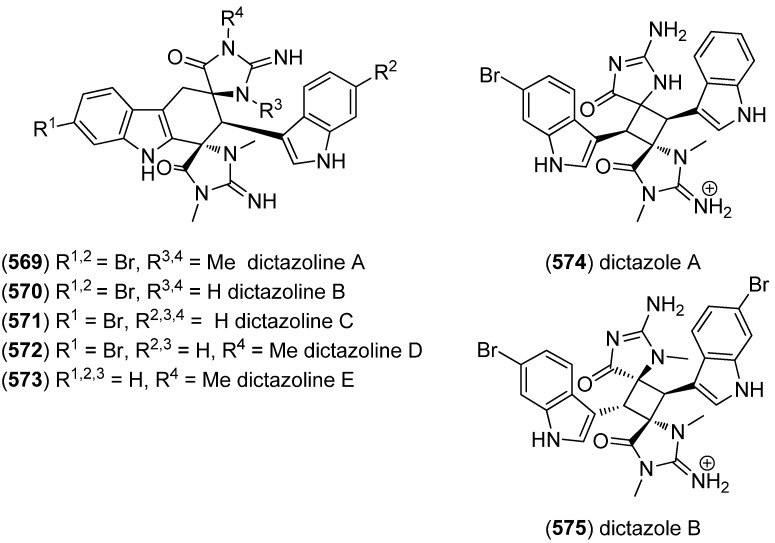
Dictazolines A–E and dictazoles A and B.

Dictazolines A–E (**569**–**573**) and dictazoles A (**574**) and B (**575**) were obtained from the marine sponge *Smenospongia cerebriformis* (Figure 100). Dictazoles are possible biosynthetic precursors of the dictazolines, and may be transformed to the latter via vinyl cyclobutane rearrangement [244,245].

**Figure 101:**
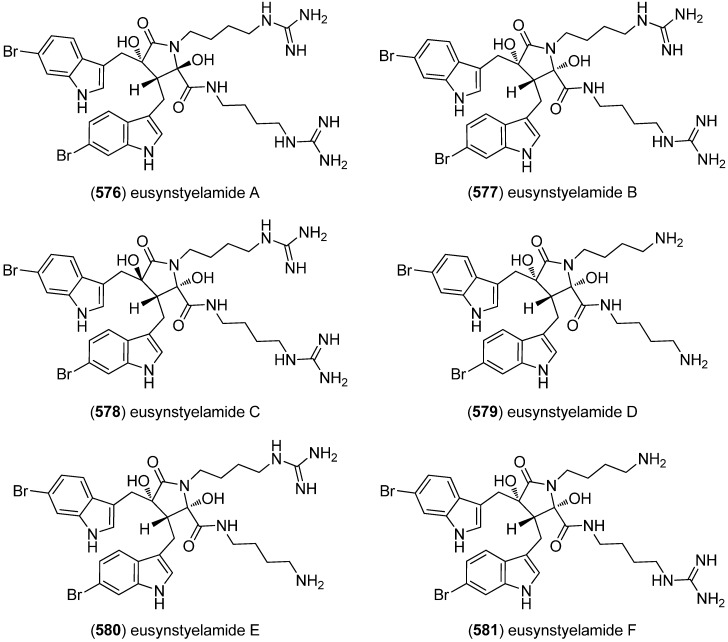
Eusynstyelamides A–F.

Eusynstyelamides A–C (**576**–**578**) were isolated from the Great Barrier Reef ascidian *Eusynstyela latericius* [246] whereas eusynstyelamides D–F (**579**–**581**) [247] were isolated from the extract of the Arctic bryozoan *Tegella cf*. *spitzbergensi*s (Figure 101). Eusynstyelamide B (**577**) displays cytotoxic activity against MDA-MB-231 cells and was identified as a potent cell cycle inhibitor [248]. It also inhibits proliferation of LNCaP cells in G2 phase and is indicated to be a topoisomerase II inhibitor in LNCaP cells [249]. Eusynstyelamides A–C (**576**–**578**) exhibit inhibitory activity against neuronal nitric oxide synthase (*n*NOS, IC_50_ 41.7, 4.3 and 5.8 μM, respectively), furthermore antimicrobial activity (*Staphylococcus aureus*, *Escherichia coli*, *Pseudomonas aeruginosa*, *Corynebacterium glutamicum*, and MRSA have been tested, IC_50_ between 6.25 and >50 µM) was reported for eusynstyelamides B (**577**) and D–F (**579**–**581**). Eusynstyelamides A (**576**) and B (**577**) show weak inhibitory activity towards pyruvate phosphate dikinase (PPDK, IC_50_ 19 and 20 mM, respectively) [246,247].

**Figure 102:**
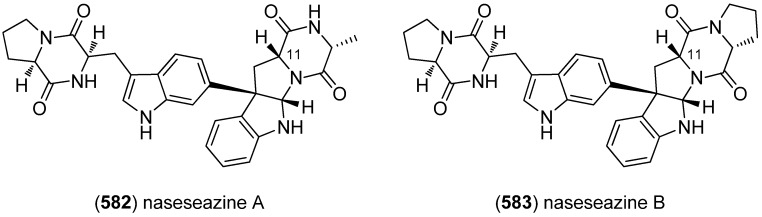
Naseseazines A and B.

Naseseazines A (**582**) and B (**583**) were obtained from *Streptomyces* sp. (CMB-MQ030) isolated from a Fijian marine sediment (Figure 102). They displayed neither antibacterial, nor antifungal or cytotoxic activities [250].

**Figure 103:**
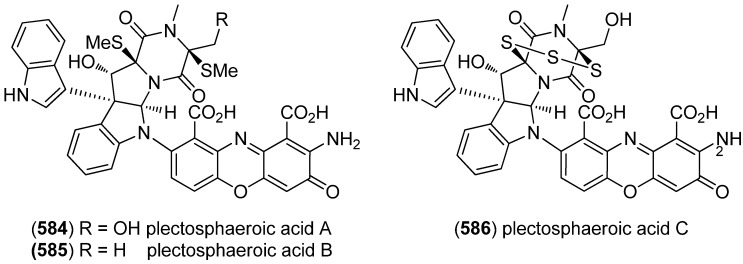
Plectosphaeroic acids A–C.

Plectosphaeroic acids A–C (**584**–**586**) were obtained from the marine fungus *Plectosphaerella cucumerina* and identified to have inhibitory activity on indoleamine 2,3-dioxygenase (IDO, IC_50_ about 2 μM) (Figure 103) [251].

**Figure 104:**
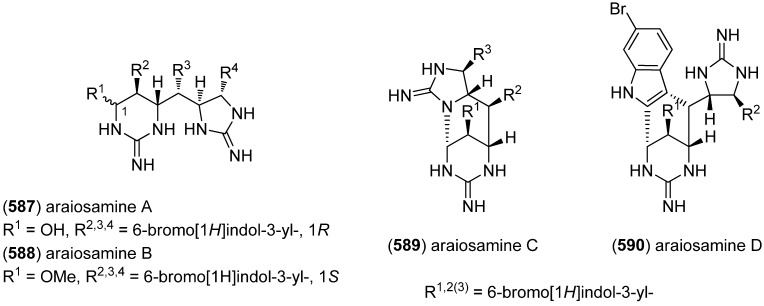
Araiosamines A–D.

Extraction of the marine sponge *Clathria (Thalysias) araiosa*, collected from Vanuatu, led to the identification of the nitrogen-rich and highly polar tris-bromoindole cyclic guanidine alkaloids araiosamines A–D (**587**–**590**) (Figure 104) [252].

**Figure 105:**
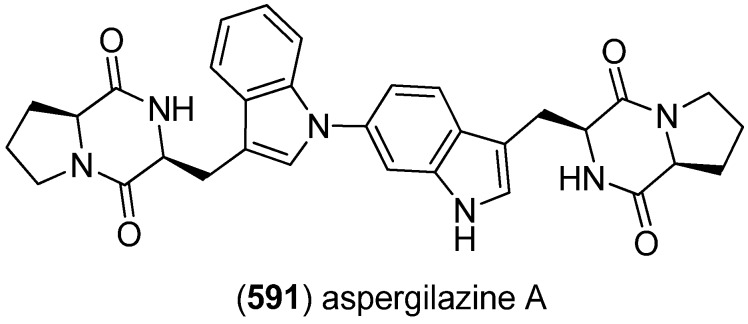
Aspergilazine A.

Aspergilazine A (**591**), a dimeric DKP with and interesting connectivity, was isolated from the marine mangrove-derived fungus *Aspergillus taichungensis* ZHN-7-07 and had a weak antiviral activity towards influenza A (H_1_N_1_) virus (34.1% inhibition at 50 μg/mL) (Figure 105) [253].

**Figure 106:**
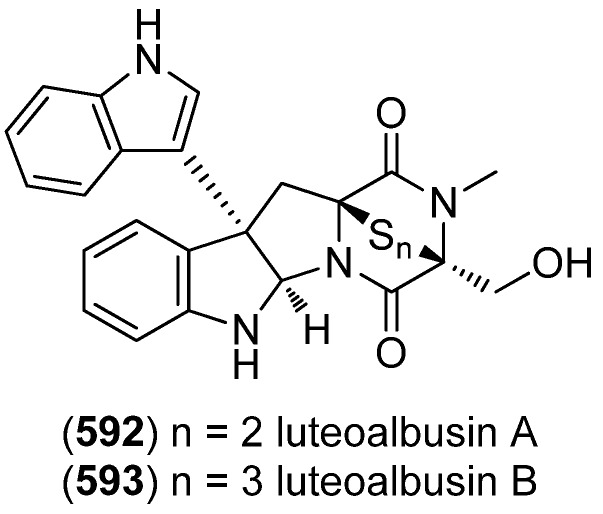
Luteoalbusins A and B.

The polythiodioxopiperazines luteoalbusins A (**592**) and B (**593**) were obtained from *Acrostalagmus luteoalbus* SCSIO F457, isolated from deep-sea sediment, and showed potent cytotoxic activities against SF-268, MCF-7, NCI-H460 and HepG-2 cell lines (IC_50_ 0.23–1.31 µM) (Figure 106) [254].

**Figure 107:**
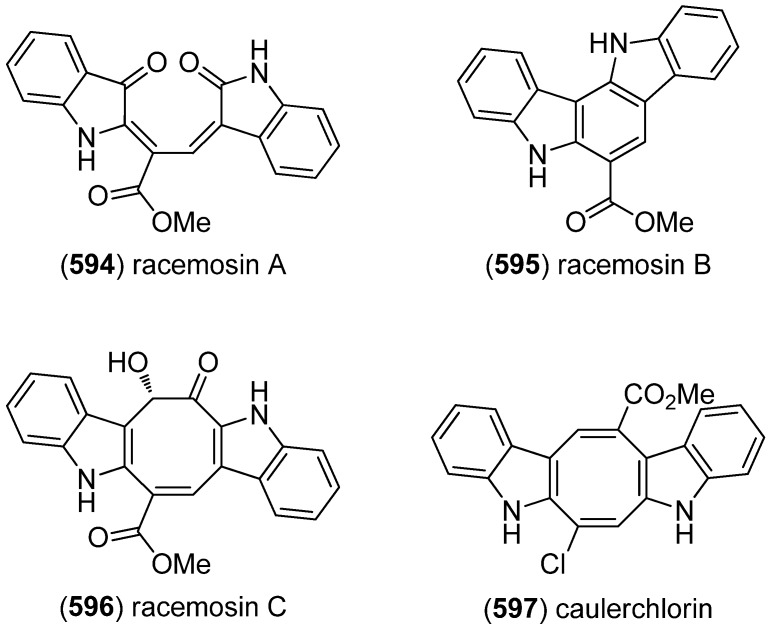
Racemosins and caulerchlorin.

Racemosins A (**594**) and B (**595**), which may be biosynthetically derived from bisindole alkaloid caulerpin, were isolated from the green alga *Caulerpa racemosa* and showed neuro-protective activity against Aβ_25–35_-induced SH-SY5Y cell damage (Figure 107) [255]. Racemosin C (**596**) [256] was isolated from the same organism and showed hPTP1B (human protein tyrosine phosphatase-1B) inhibitory activity (IC_50_ 5.86 μM), as well as caulerchlorin (**597**) [257] which exhibited weak antifungal activity against *Cryptococcus neoformans* strain 32609 (MIC_80_ 16 μg/mL).

**Figure 108:**
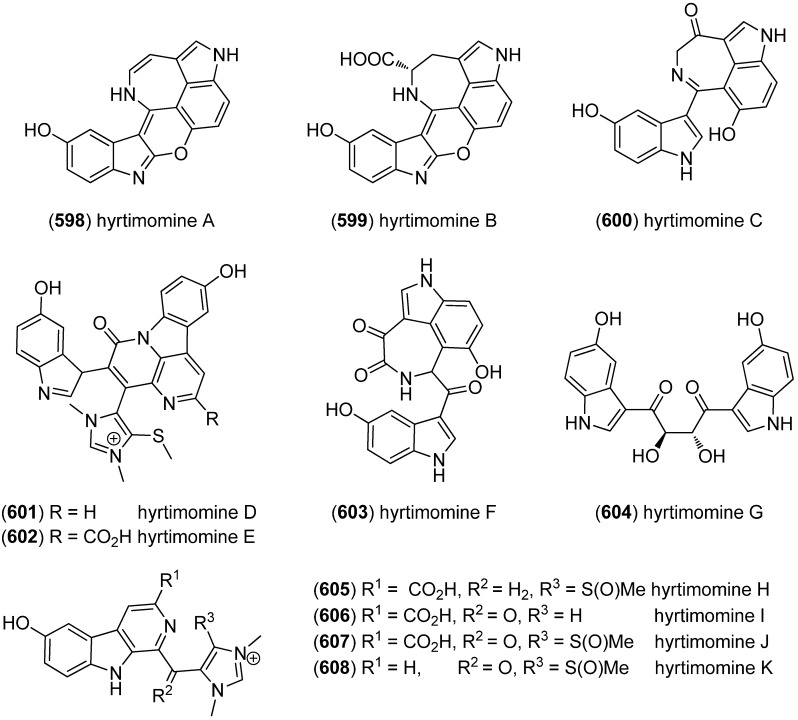
Hyrtimomines A–K.

Hyrtimomines A–K (**598**–**608**) were isolated from the Okinawan marine sponge *Hyrtios* sp. (Figure 108) [258,259,260]. Hyrtimomine A (**598**) displayed cytotoxic activity towards KB and L1210 cells [258]. Hyrtimomines D (**601**) and E (**602**) showed antimicrobial effects against *Candida albicans*, *Cryptococcus neoformans,* *Staphylococcus aureus* and *Trichophyton mentagrophytes* (MIC 4–16 μg/mL) [259]. Several antimicrobial activities were also reported for hyrtimomines A, B, F, G and I [260].

**Figure 109:**
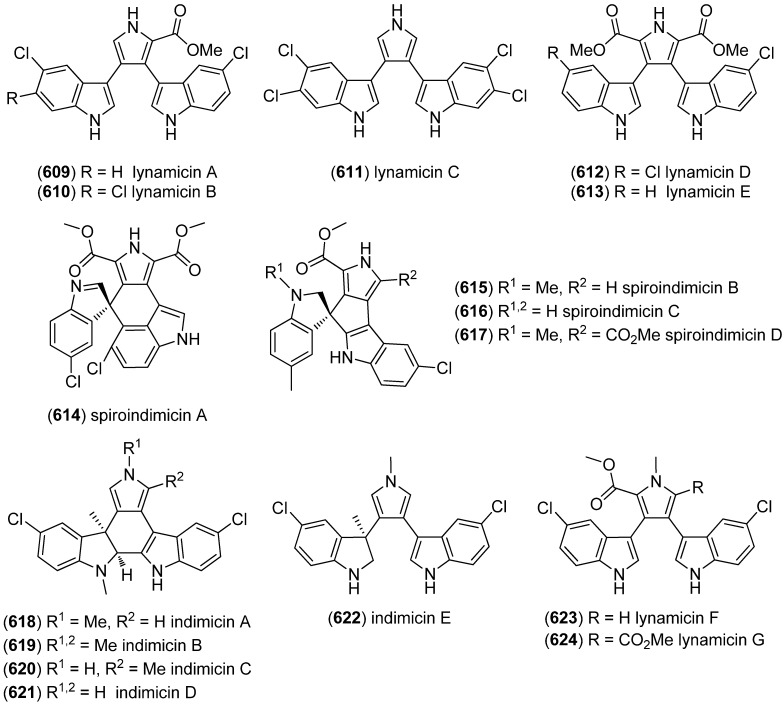
Lynamicins A–G, spiroindimicins A–D and indimicins A–E.

The marine actinomycete NPS12745, isolated from a marine sediment (San Diego, CA, USA), provided chlorinated bisindole pyrroles lynamicins A–E (**609**–**613**) (Figure 109). They exhibited a broad spectrum of antimicrobial activities, also against methicillin-resistant *Staphylococcus aureus* and vancomycin-resistant *Enterococcus faecium* [261]. The structurally reamarkable pyrrole-fused spirocyclic bisindole alkaloids spiroindimicins A–D (**614**–**617**) were isolated from the deep-sea-derived *Streptomyces sp.* SCSIO 03032. Spiroindimicins B–D (**615**–**617**) exhibited moderate cytotoxicity against the tumor cell lines CCRF-CEM, B16, HepG2 and H460 [262]. The same producer was also the source of indimicins A–E (**618**–**622**) as well as of lynamicins F (**623**) and G (**624**). None of these compounds exhibited antimicrobial activities, but indimicin B (**619**) showed cytotoxic effects against the MCF-7 breast cancer cell line [263]. The indimicins A–D are structurally similar to the staurosporin core but a planar arrangement is prevented by an angular methyl group. Spiroindimicins C (**616**), D (**617**) and lynamicin D (**612**) were proposed to be potent topoisomerase II, cathepsin K, cytochrome P450 3A4, aromatase P450, protein kinase and histone deacetylase inhibitors based on an *in silico* molecular docking approach [264].

**Figure 110:**
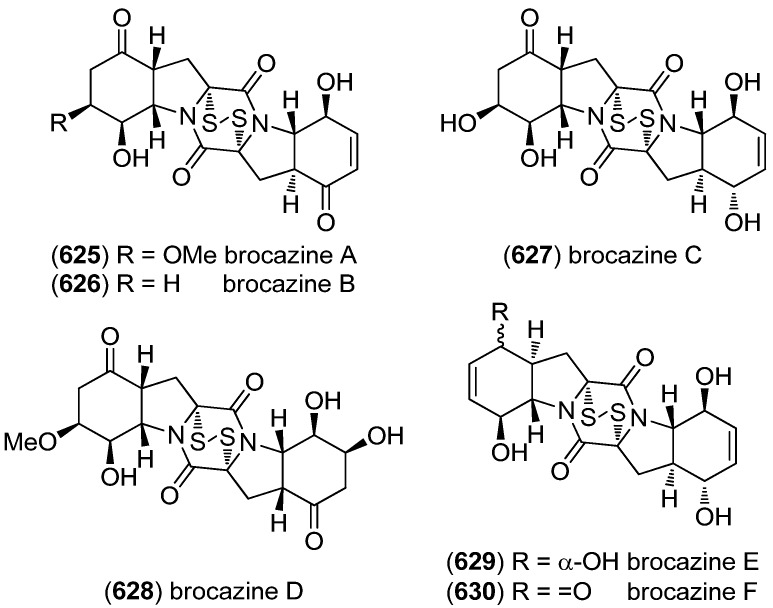
Brocazines A–F.

Brocazines A–F (**625**–**630**), epidithiodiketopiperazines with a probable bigenetic relation to tryptophan, were isolated from the extract of the mangrove-derived *Penicillium brocae* MA-231 and exhibited cytotoxicity against several tumor cell lines (Figure 110) [265].

### 2.4. Anellated Indoles

**Figure 111:**
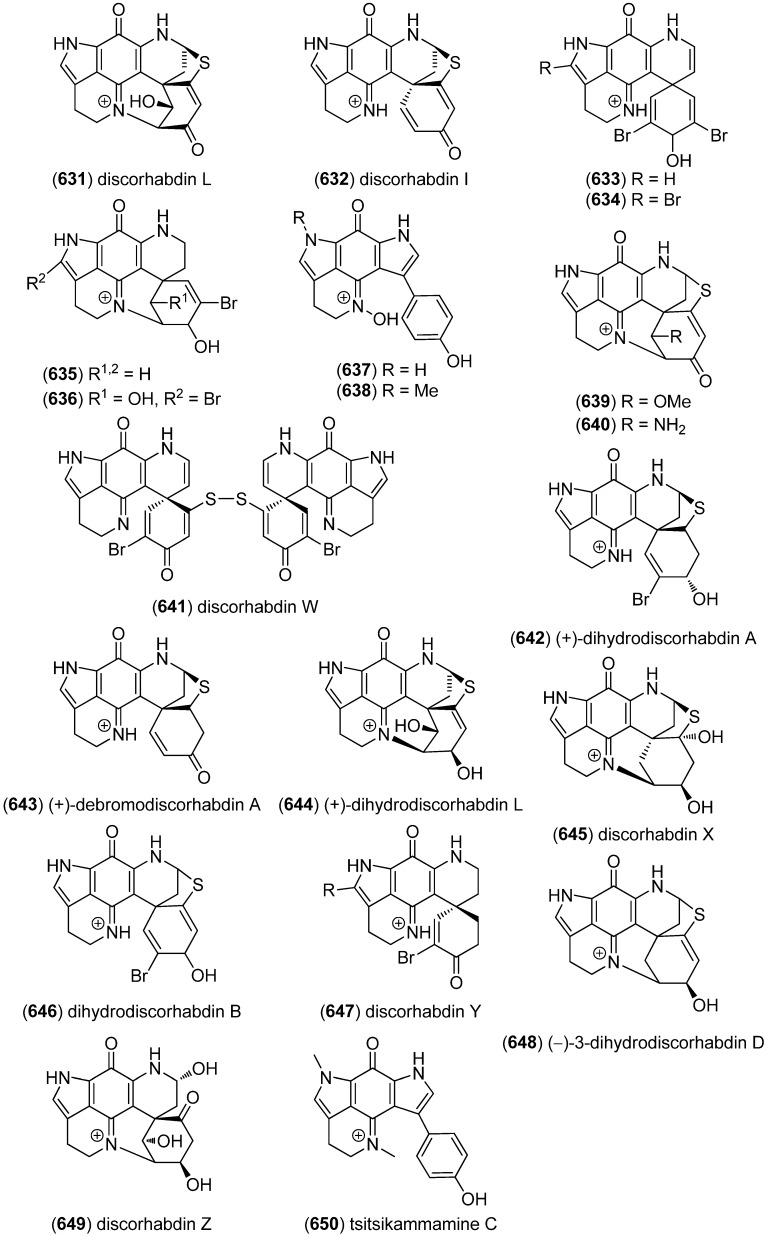
Discorhabdin and tsitsikammamine derivatives.

In the anellated indole alkaloids, a single indole core is fused to other (hetero)cyclic ring systems that are not prenyl derived. For example, the discorhabdins L (**631**) and I (**632**) were isolated from the marine sponge *Latrunculia brevis* and exhibited potent cytotoxic activity towards 14 tumor cell lines (e.g., HT-29 colon cell line: GI_50_ 0.12 and 0.35 μM, respectively) (Figure 111) [266]. The discorhabdin derivatives 3-dihydro-7,8-dehydrodiscorhabdin C (**633**), 14-bromo-3-dihydro-7,8-dehydrodiscorhabdin C (**634**), discorhabdin V (**635**), 14-bromo-1-hydroxydiscorhabdin V (**636**), tsitsikammamine A *N*-18 oxime (**637**), tsitsikammamine B *N*-18 oxime (**638**), 1-methoxydiscorhabdin D (**639**), and 1-aminodiscorhabdin D (**640**) were isolated from extracts of four South African latrunculid sponges, *Tsitsikamma pedunculata*, *T. favus, Latrunculia bellae*, and *Strongylodesma algoaensis*. (**633**–**635**), (**639**) and (**640**) showed cytotoxicity against human colon tumor HCT-116 cells. [267] Discorhabdin W (**641**), the first dimeric congener, was obtained from a New Zealand sponge *Latrunculia* sp. and showed strong cytotoxic effects towards P388 leukemia cells [268]. (+)-Dihydrodiscorhabdin A (**642**), (+)-debromodiscorhabdin A (**643**), (+)-dihydrodiscorhabdin L (**644**) and (+)-discorhabdin X (**645**) were obtained from southern Australian marine sponges of the genera *Higginsia* and *Spongosorites* [269]. Dihydrodiscorhabdin B (**646**) and discorhabdin Y (**647**) were isolated from a deep-water Alaskan sponge (*Latrunculia* sp.) [270]. (−)-3-Dihydrodiscorhabdin D (**648**) and (−)-discorhabdin Z (**649**) were isolated from the Korean marine sponge *Sceptrella* sp. [271]. Tsitsikammamine C (**650**) was isolated from the Australian marine sponge *Zyzzya* sp. and exhibited strong cytotoxic and antiparasitic activity [272].

**Figure 112:**
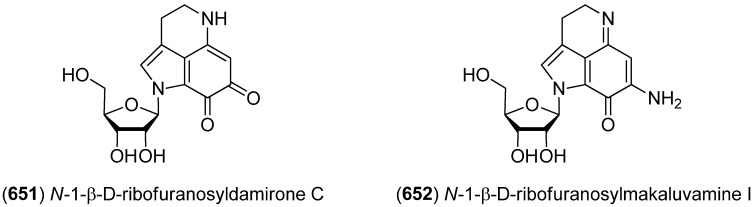
*N*-1-β-d-Ribofuranosyldamirone C and *N*-1-β-d-ribofuranosylmakaluvamine I.

*N*-1-β-d-Ribofuranosyldamirone C (**651**) and *N*-1-β-d-ribofuranosylmakaluvamine I (**652**) were obtained from the South African latrunculid sponge *Strongylodesma aliwaliensis* and showed activity against esophageal cancer cell lines WHCO1, WHCO6, and KYSE30 (IC_50_ 1.6–85.5 µM) (Figure 112) [273,274].

**Figure 113:**
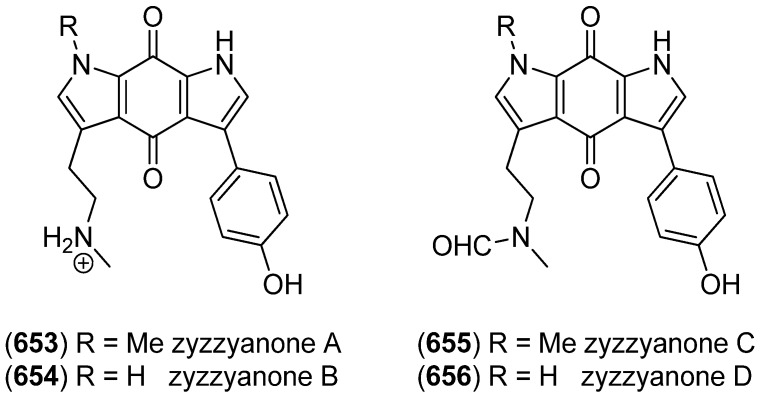
Zyzzyanones A–D.

Zyzzyanones A (**653**) [275] and B–D (**654**–**656**) [276], bearing a pyrrolo[3,2-*f*]indole-4,8(1*H*,7*H*)-dione skeleton, were isolated from the Australian marine sponge *Zyzzya fuliginosa* (Figure 113). Zyzzyanones A–D (**653**–**656**) exhibit moderate cytotoxic activity against mouse Ehrlich carcinoma cells, as well as UV-protective activity [275,276,277].

**Figure 114:**
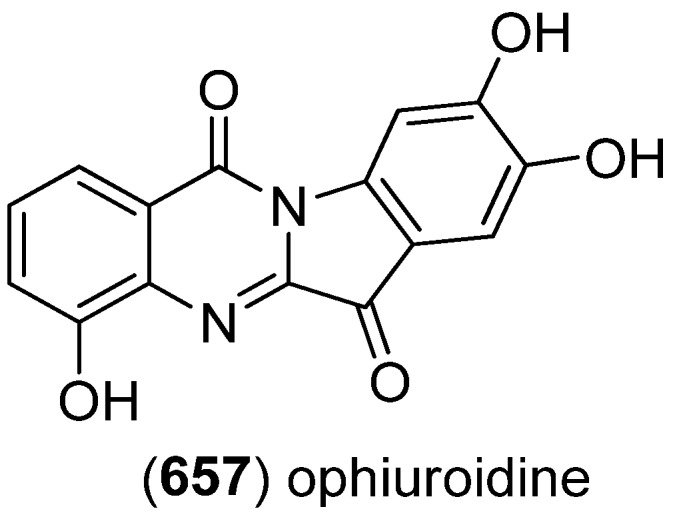
Ophiuroidine.

Ophiuroidine (**657**) has an indolo[2,1-*b*]quinazoline-6,12-dione skeleton and was obtained from the Caribbean ophiuroid *Ophiocoma riisei* (Figure 114) [278].

**Figure 115:**
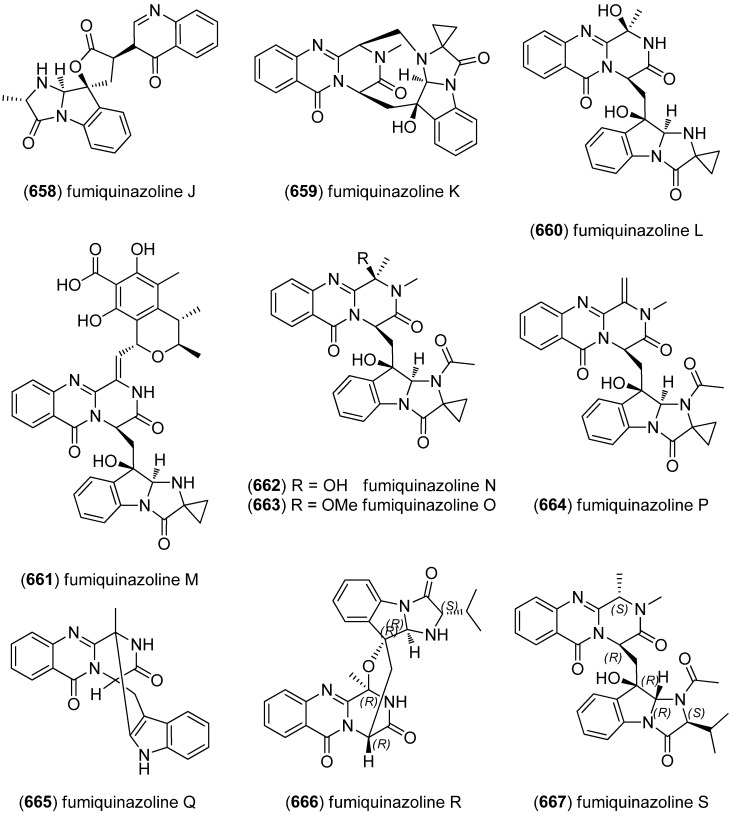
Fumiquinazolines K–S.

**Figure 116:**
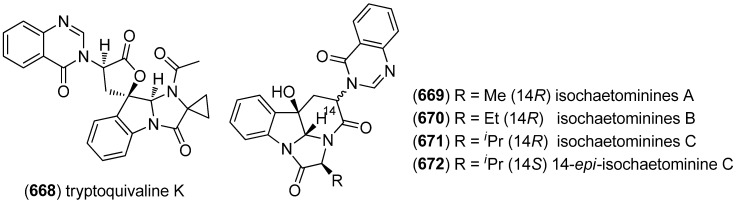
Tryptoquivaline K, isochaetominines A–C and 14-*epi*-isochaetominine C.

Fumiquinazoline J (**658**) was isolated from the marine-derived fungal strain *Aspergillus fumigatus* H1-04 and exhibited cytotoxic activities against the cell lines tsFT210, P388, HL-60, A549 and BEL-7402 (Figure 115) [279]. Fumiquinazolines K–P (**659**–**664**), together with tryptoquivaline K (**668**, Figure 116), were isolated from the Mediterranean sponge-derived fungi *Aspergillus* sp. [280] Compounds **665** and **666** were simultaneously isolated from the soft coral-derived fungus *A. fumigatus* KMM 4631 and a gorgonian-derived fungus (*Scopulariopsis* sp.), respectively, and were initially doubly designated as fumiquinazolines K and L [281,282]. These names have been revised to fumiquinazolines Q (**665**) and R (**666**). Fumiquinazoline S (**667**) was isolated from marine-derived *Aspergillus* sp., together with isochaetominines A–C (**669**–**671**) and 14-*epi*-isochaetominine C (**672**) (Figure 116). Fumiquinazolines F, L (**660**), S (**667**) and isochaetominines (**669**–**672**) showed weak inhibition against Na^+^/K^+^-ATPase (IC_50_ 17–78 μM) [283].

**Figure 117:**
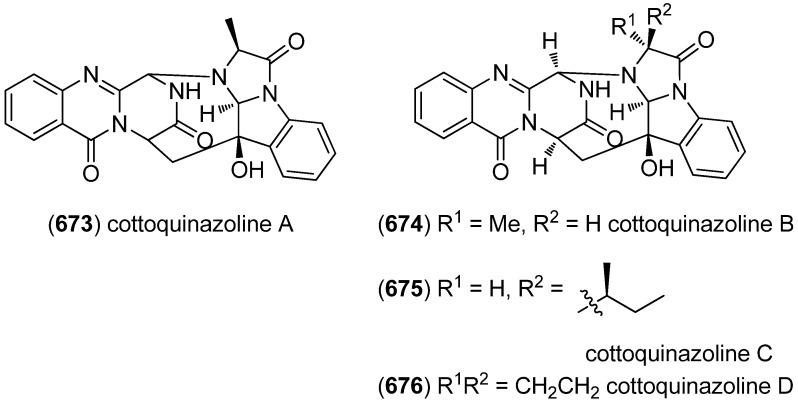
Cottoquinazolines A–D.

Cottoquinazolines A–D (**673**–**676**) were obtained from the marine-derived fungus *Aspergillus versicolor* strains MST-MF495 and LCJ-5-4 (Figure 117). Cottoquinazoline D (**676**) has been reported to show antifungal activity against *Candida albicans* (MIC 22.6 μM) [284,285].

**Figure 118:**
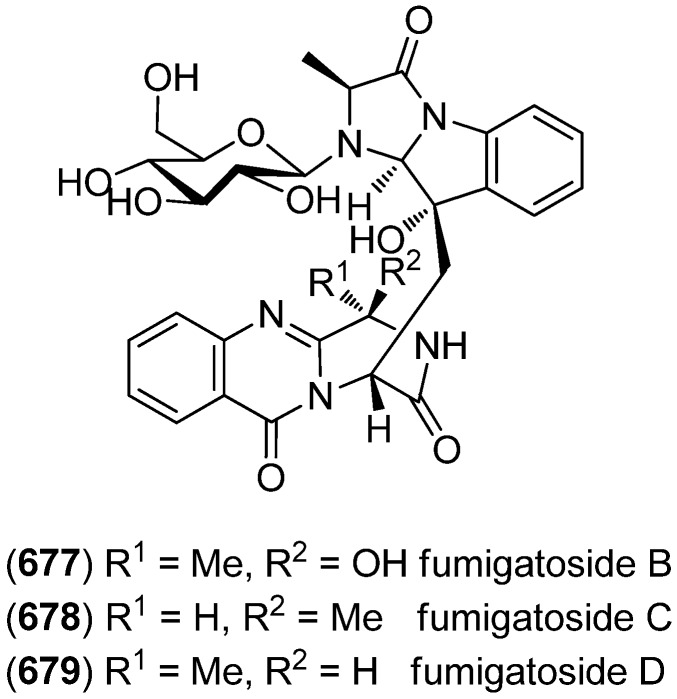
Fumigatosides B–D.

Fumiquinazolines fumigatosides B–D (**677**–**679**) were obtained from the fungus *Aspergillus fumigatus* derived from the giant Japanese jellyfish *Nemopilema nomurai* (Figure 118). They are biosynthetically derived from anthranilic acid, tryptophan, l-alanine and d-glucose. Neither antibacterial nor cytotoxic effects were observed [286].

**Figure 119:**
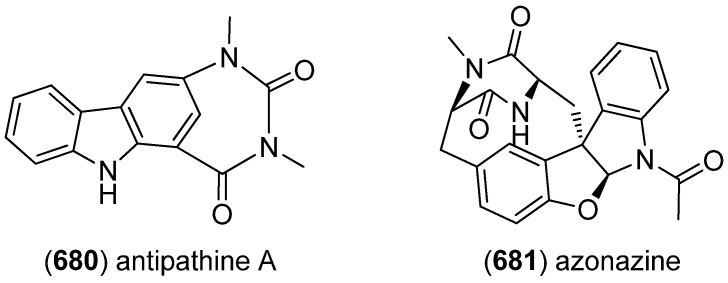
Antipathine A and azonazine.

Antipathine A (**680**) was isolated from the South China Sea black coral *Antipathes dichotoma* and exhibited cytotoxic effects towards the human carcinoma cell lines SGC-7901 and Hep-G2 (Figure 119) [287]. Azonazine (**681**), which contains a unique hexacyclic bridged diketopiperazine structure, was obtained from the Hawaiian marine sediment-derived fungus *Aspergillus insulicola* and exhibited anti-inflammatory activity via inhibition of NF-κB (nuclear factor kappa-light-chain-enhancer of activated B cells) luciferase and nitrite production [288].

**Figure 120:**
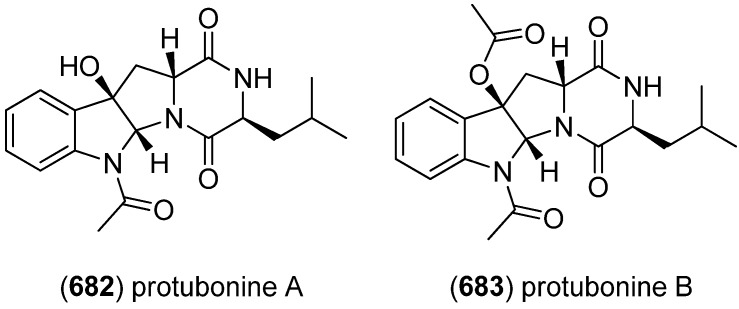
Protubonines A and B.

Protubonines A (**682**) and B (**683**) were isolated from the marine-derived fungus *Aspergillus* sp. SF-5044 and showed no cytotoxic effects against the tumor cell lines HL-60, MDA-MB-231, Hep3B, 3Y1 and K562 (IC_50_ > 250 μM) (Figure 120) [289].

**Figure 121:**
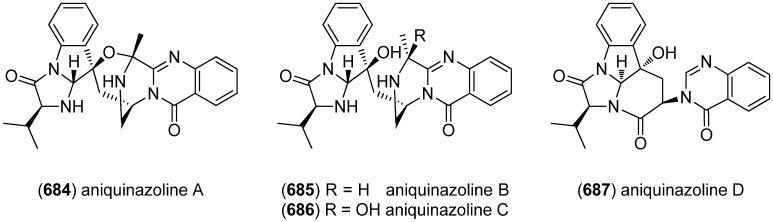
Aniquinazolines A–D.

Aniquinazolines A–D (**684**–**687**) were obtained from the marine-derived *Aspergillus nidulans* MA-143 and displayed potent toxicity against brine shrimp (*Artemia salina*, LC_50_ 1.27, 2.11, 4.95 and 3.42 μΜ, respectively), but did not show antibacterial and cytotoxic effects (Figure 121) [290].

**Figure 122:**
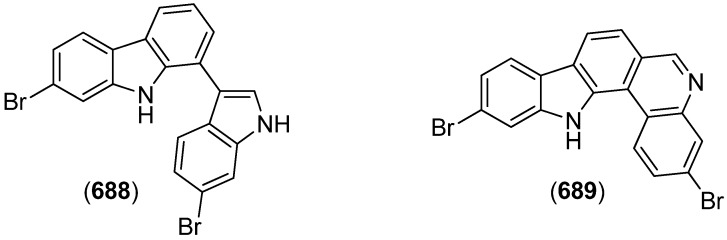
7-Bromo-1-(6-bromo-1*H*-indol-3-yl)-9*H*-carbazole and 3,11-dibromo-13*H*-indolo[3,2-*k*]phenanthridine.

7-Bromo-1-(6-bromo-1*H*-indol-3-yl)-9*H*-carbazole (**688**) and 3,11-dibromo-13*H*-indolo[3,2-*k*]phenanthridine (**689**) were isolated from the marine sponge *Penares* sp. (South China Sea) (Figure 122). 7-Bromo-1-(6-bromo-1*H*-indol-3-yl)-9*H*-carbazole (**688**) exhibited cytotoxicity towards HL-60 and HeLa human tumor cells (IC_50_ 16.1 and 33.2 μM, respectively) [291].

**Figure 123:**
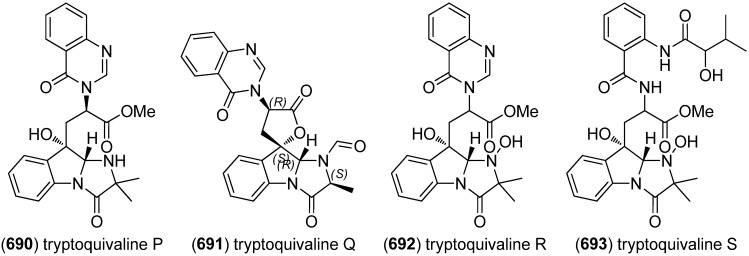
Tryptoquivalines P–S.

Tryptoquivalines P–S (**690**–**693**) have been isolated from a marine-derived fungus *Neosartorya* sp. HN-M-3 (Figure 123) [292,293].

**Figure 124:**
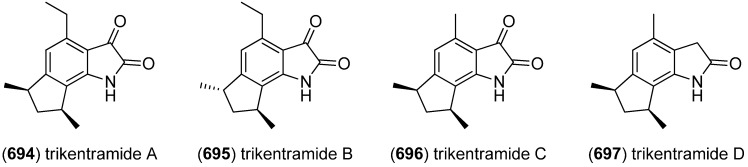
Trikentramides A–D.

Analysis of the Australian spider sponge *Trikentrion flabelliforme* led to the isolation of trikentramides A–D (**694**–**697**) (Figure 124). Until now, no biological evaluation of these compounds has been reported [294].

**Figure 125:**
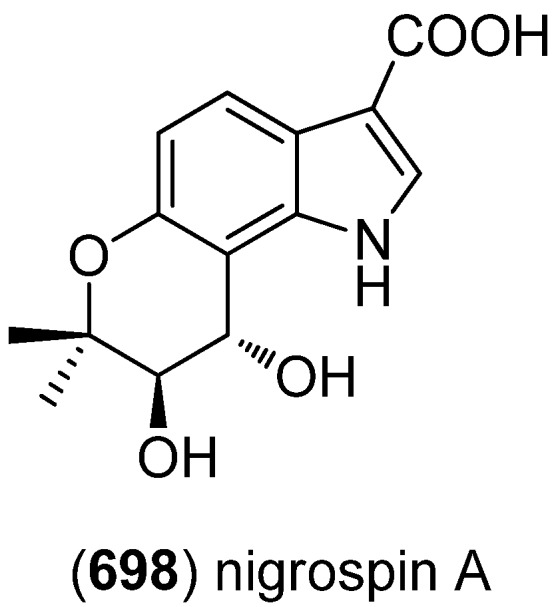
Nigrospin A.

Nigrospin A (**698**) was recently isolated from the marine-derived fungus *Nigrospora oryzae* SCSGAF 0111, the terrestrial forms of which are known plant pathogens (Figure 125) [295].

**Figure 126:**
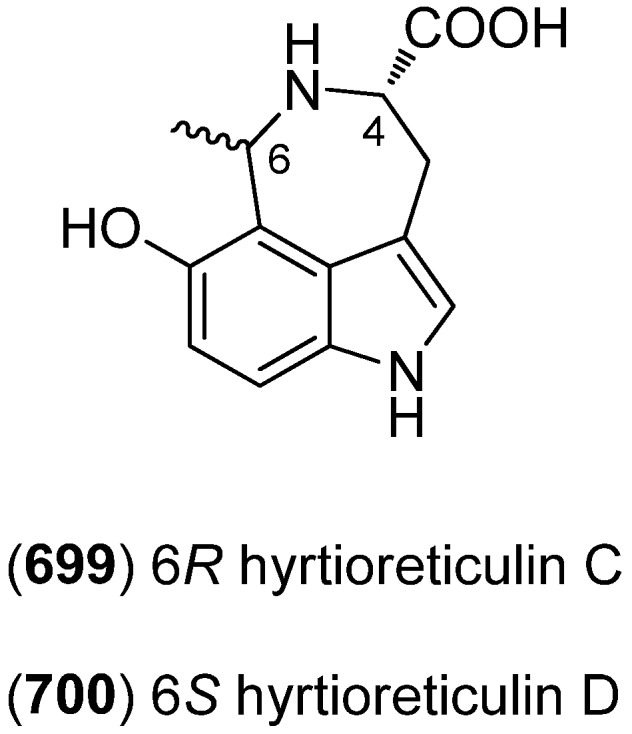
Hyrtioreticulins A–F.

Hyrtioreticulins A–F were isolated from *Hyrtios reticulatus*. Hyrtioreticulins A, B, E and F (**779**–**782**) possess a β-carboline framework (see Figure 143), hyrtioreticulins C (**699**) and D (**700**) belong to the group of azepino-indole-type alkaloids (Figure 126) [296,297].

**Figure 127:**
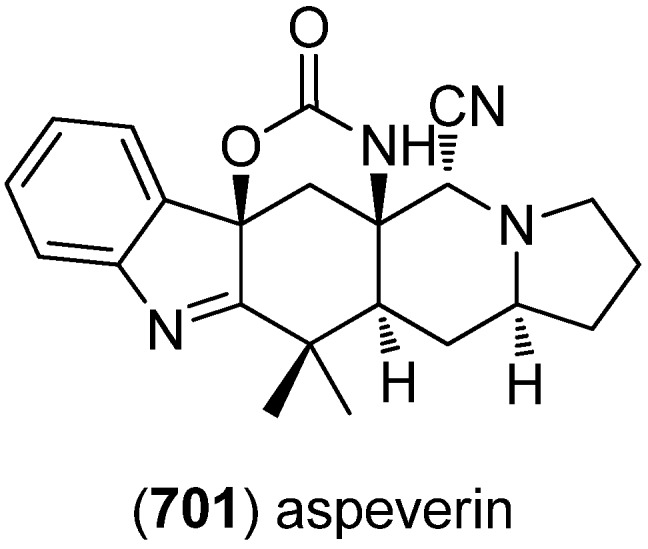
Aspeverin.

*Aspergillus versicolor* dl-29, isolated from the marine green alga *Codium fragile*, was the source of aspeverin (**701**), which inhibits the growth of the phytoplankton microalga *Heterosigma akashiwo* as well as brine shrimp *Artemia salina* and the bacterial species *Vibrio ichthyoenteri*, *Proteus mirabilis*, *Enterobacter cloacae* and *Bacillus cereus* (Figure 127) [298].

**Figure 128:**
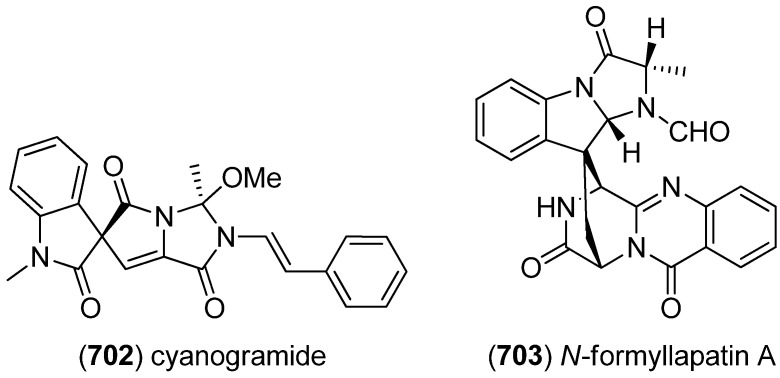
Cyanogramide and *N*-formyllapatin A.

Cyanogramide (**702**), a spirocyclic pyrrolo[1,2-*c*]imidazole, was obtained from *Actinoalloteichus cyanogriseus* WH1-2216-6 and displayed weak cytotoxic effects towards K562, MCF-7, KB, and the MDR cell lines K562/A02, MCF-7/Adr and KB/VCR (IC_50_ 12.9, 18.5, 16.8, 10.2, 36.0 and 25.6 μM, respectively) and could reverse the multidrug resistance of K562/A02, MCF-7/Adr, and KB/VCR cells (Figure 128) [299]. *N*-Formyllapatin A (**703**) was identified from *Penicillium adametzioides* and did not show any appreciable antibacterial activity [300].

**Figure 129:**
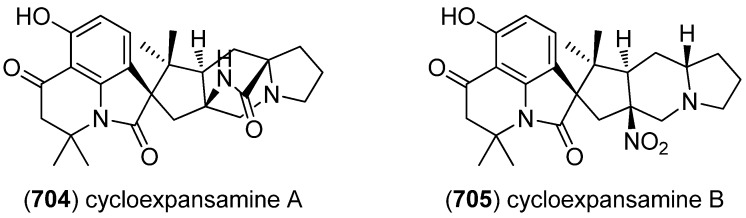
Cycloexpansamines A and B.

Spiroindolinone alkaloids cycloexpansamines A (**704**) and B (**705**) were obtained from a marine isolate of *Penicillium* sp. (SF-5292) (Figure 129) [301].

**Figure 130:**
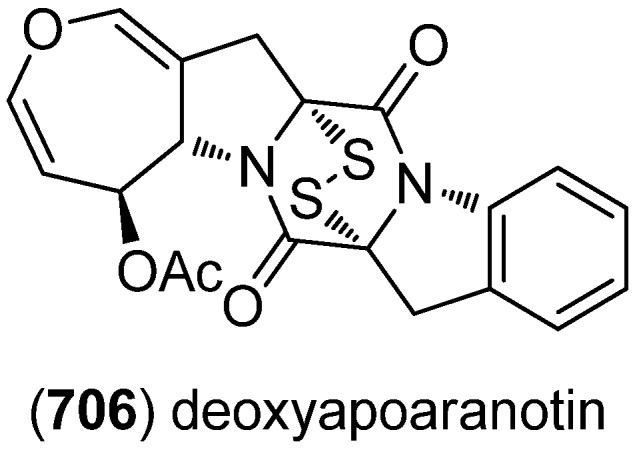
Deoxyapoaranotin.

The Epidithiodiketopiperazine deoxyapoaranotin (**706**), together with acetylaranotin and acetylapoaranotin, was separated from *Aspergillus* sp. KMD 901, which was obtained from a marine sediment from the East Sea of Korea (Figure 130). Deoxyapoaranotin (**706**) was found to have cytotoxic activity towards HCT-116 colon cancer cell line via apoptosis-inducing effects [302].

**Figure 131:**
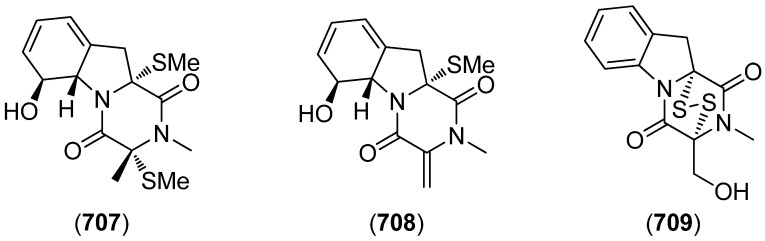
Gliotoxin derivatives.

The gliotoxin derivative dehydroxy*bis*dethio*bis*(methylthio)gliotoxin (**707**) was isolated from the marine-derived fungus *Pseudallescheria* sp. (Figure 131) [303]. Bis(dethio)-10a-methylthio-3a-deoxy-3,3a-didehydrogliotoxin (**708**) and 6-deoxy-5a,6-didehydrogliotoxin (**709**), were obtained from the Japanese deep sea-derived fungus *Penicillium* sp. strain JMF034 and 6-deoxy-5a,6-didehydrogliotoxin (**709**) showed cytotoxic effects against P388 murine leukemia cells (IC_50_ 3.4 and 0.058 µM) [304].

**Figure 132:**
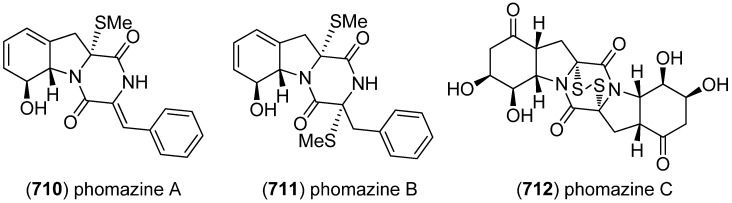
Phomazines A–C.

Phomazines A–C (**710**–**712**) were isolated from the mangrove-derived endophytic fungus, *Phoma* sp. OUCMDZ-1847 (Figure 132). Phomazine B (**711**) exhibited moderate cytotoxic activities towards cancer cell lines HL-60, HCT-116, K562, MGC-803 and A549 (IC_50_ between 8.5 and >10 µM) [305].

#### β-Carbolines

β-Carbolines are a large group of indole alkaloids and widespread in nature. Their producers include plants, insects, marine organisms, *etc.* They provide a large spectrum of pharmacological activities [306].

**Figure 133:**
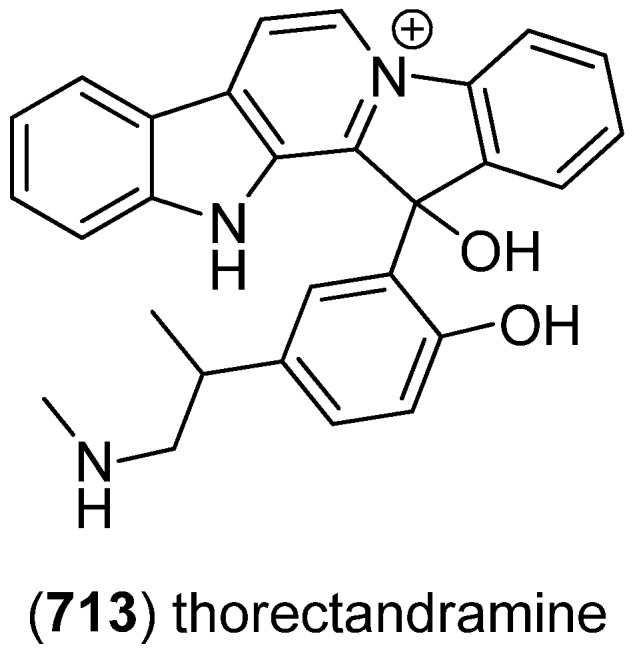
Thorectandramine.

Thorectandramine (**713**) was obtained from the Palauan sponge *Thorectandra* sp. and exhibited weak cytotoxic activity towards MCF-7, OVCAR-3 and A549 cell lines (Figure 133) [307].

**Figure 134:**
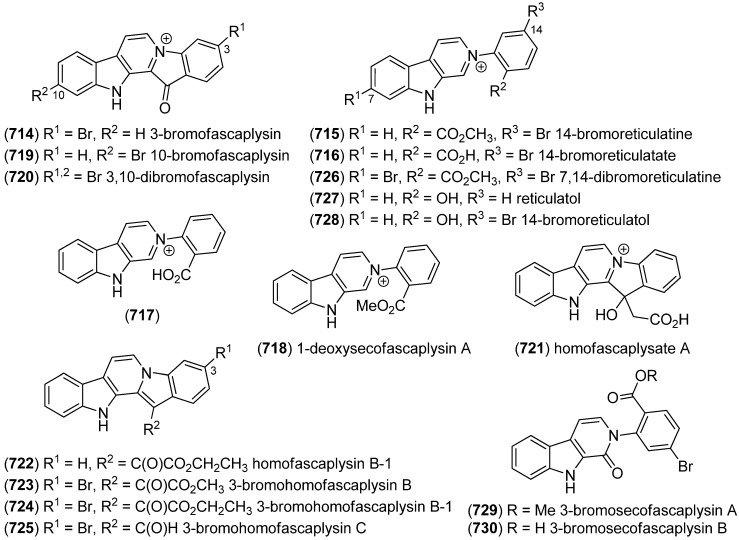
Fascaplysin and reticulatine derivatives.

3-Bromofascaplysin (**714**), 14-bromoreticulatine (**715**) and 14-bromoreticulatate (**716**) were isolated from collections of the sponge *Fascaplysinopsis reticulata* and the tunicate *Didemnum* sp. (Figure 134) [308]. 3-Bromofascaplysin (**714**) was found to exhibit anticancer activity towards the cell lines HL-60, THP-1, HeLa, MDA-MB-231, DLD-1, SNU-C4 and SK-MEL-28, which was identified to be caspase-3, -8 and -9-mediated [309]. **717** and 1-deoxysecofascaplysin A (**718**) were obtained from the marine sponge *Thorectandra* sp. and showed cytotoxic activities [310]. 10-Bromofascaplysin (**719**), 3,10-dibromofascaplysin (**720**), homofascaplysate A (**721**), homofascaplysin B-1 (**722**), 3-bromohomofascaplysins B (**723**), B-1 (**724**) and C (**725**), 7,14-dibromoreticulatine (**726**), reticulatol (**727**), 14-bromoreticulatol (**728**) and 3-bromosecofascaplysins A (**729**) and B (**730**) were isolated from four collections of sponge *Fascaplysinosis reticulata* and two *Didemnum* sp. tunicates [311].

**Figure 135:**
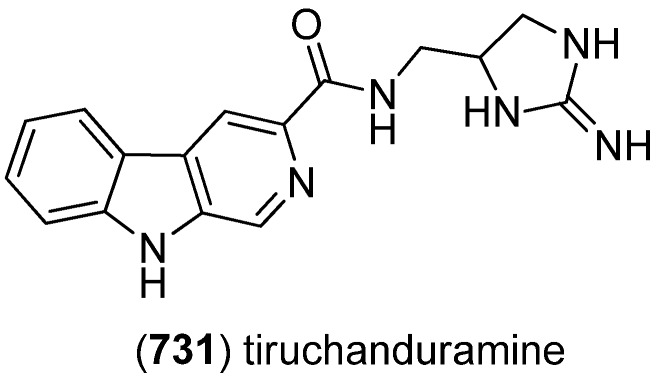
Tiruchanduramine.

Tiruchanduramine (**731**) was isolated from the Indian ascidian *Synoicum macroglossum* and was reported to be a promising α-glucosidase inhibitor (IC_50_ 78.2 μg/mL) (Figure 135) [312].

**Figure 136:**
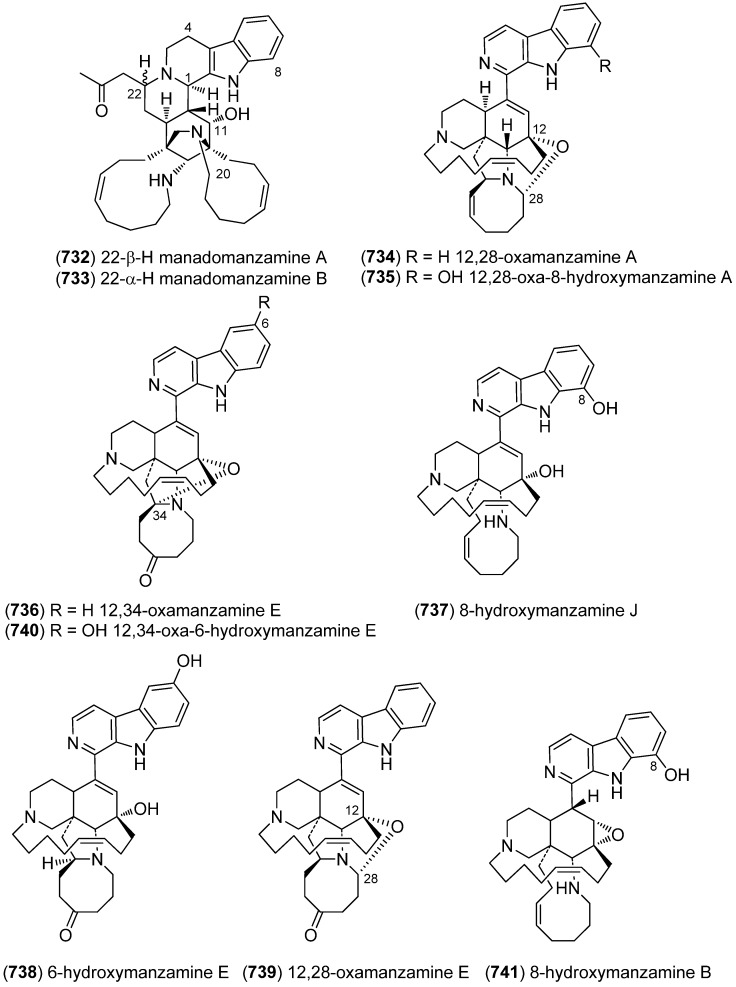
Manadomanzamines A and B and manzamine derivatives.

Manadomanzamines A (**732**) and B (**733**) were obtained from the Indo-Pacific sponge *Acanthostrongylophora* sp. and were found to exhibit strong activity against *Mycobacterium tuberculosis* and HIV-1 (Figure 136). Additionally, they show antifungal and cytotoxic, but only weak antimalarial, activity [313]. Furthermore, manzamine-type alkaloids 12,28-oxamanzamine A (**734**), 12,28-oxa-8-hydroxymanzamine A (**735**), 12,34-oxamanzamine E (**736**), 8-hydroxymanzamine J (**737**), 6-hydroxymanzamine E (**738**), 12,28-oxamanzamine E (**739**), 12,34-oxa-6-hydroxymanzamine E (**740**) and 8-hydroxymanzamine B (**741**) have been isolated from *Acanthostrongylophora* sp. [314,315,316].

**Figure 137:**
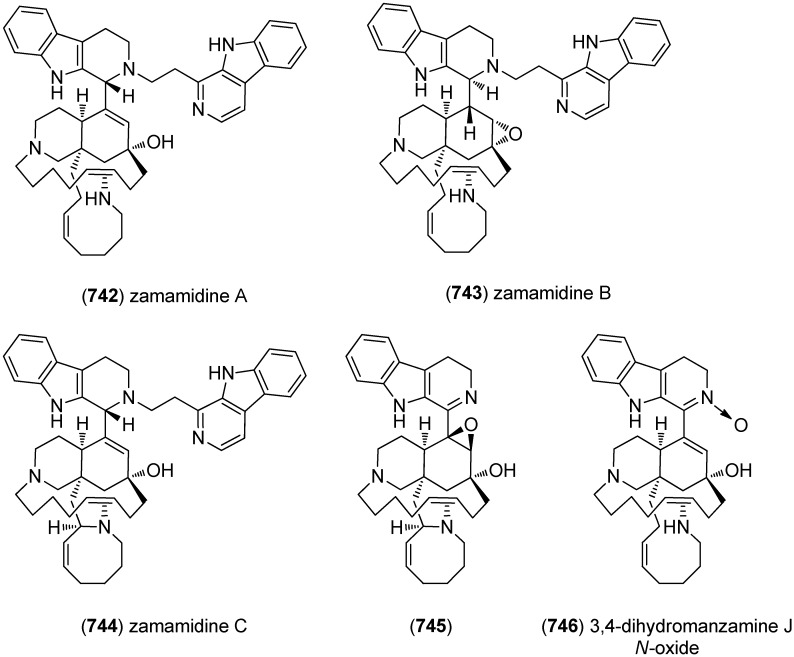
Zamamidines A–C and manzamine derivatives.

Zamamidines A–C (**742**–**744**), 3,4-dihydro-6-hydroxy-10,11-epoxymanzamine A (**745**) and 3,4-dihydromanzamine J *N*-oxide (**746**) were obtained from the Okinawan marine sponge *Amphimedon* sp. (Figure 137). Zamamidines A (**742**) and B (**743**) exhibit cytotoxic activity against P388 murine leukemia (IC_50_ 13.8 and 14.8 μg/mL, respectively), but not against KB human epidermoid carcinoma cells. Zamamidine C (**744**), 3,4-dihydro-6-hydroxy-10,11-epoxymanzamine A (**745**), and 3,4-dihydromanzamine J *N*-oxide (**746**) displayed cytotoxic effects towards P388, L1210 and KB cell lines. Zamamidines **342**–**344** and 3,4-dihydromanzamine J *N*-oxide (**746**) exhibited antitrypanosomal activity against *Trypanosoma brucei brucei* and antimalarial activity against *Plasmodium falciparum* [317,318].

**Figure 138:**
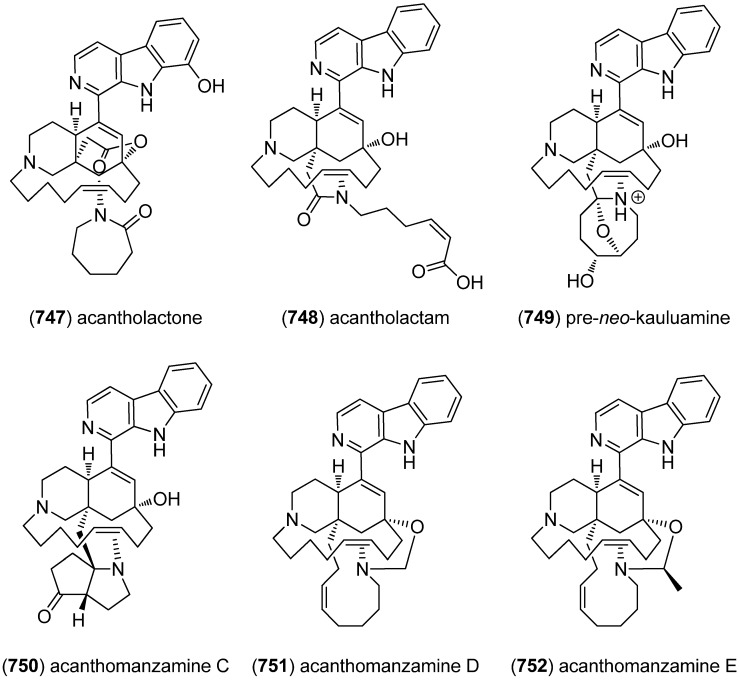
Acantholactone, acantholactam, pre-*neo*-kauluamine and acanthomanzamines C–E.

Acantholactone (**747**) was isolated from *Acanthostrongylophora* sp. and bears δ-lactone and ε-lactam rings (Figure 138) [319]. Acantholactam (**748**) and pre-*neo*-kauluamine (**749**) were obtained from the marine sponge *Acanthostrongylophora ingens*. Acantholactam (**748**) exhibited inhibitory effects against the accumulation of cholesterol esters in macrophages, whereas pre-*neo*-kauluamine (**749**) showed proteasome inhibitory activity [320]. Further manzamine derivatives, acanthomanzamines A–E, were obtained *A. ingens*. Acanthomanzamines A and B possess tetrahydroisoquinoline moieties and only acanthomanzamines C–E (**750**–**752**) are indole-derived. Acanthomanzamines were found to have cytotoxic and inhibitory activity on accumulation of cholesterol ester in macrophages [321].

**Figure 139:**
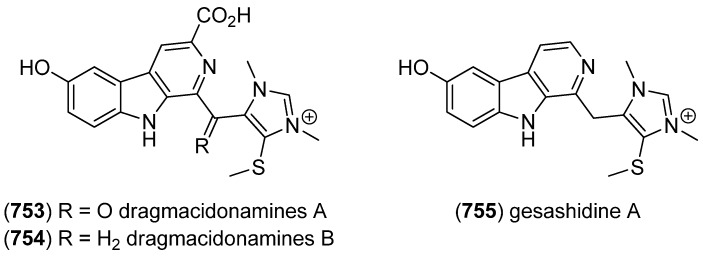
Dragmacidonamines A and B and gesashidine A.

Dragmacidonamines A (**753**) and B (**754**) were obtained from the sponge *Dragmacidon* sp. Dragmacidonamine A (**753**) exhibited cytotoxicity against cell line L5178Y (Figure 139) [322]. Gesashidine A (**755**) was isolated from the Okinawan marine sponge *Thorectidae* sp. (SS-1035) and showed antibacterial activity against *Micrococcus luteus*, but no cytotoxicity [323].

**Figure 140:**
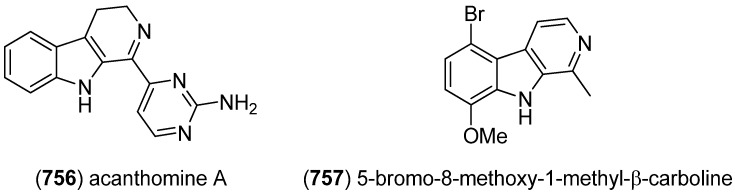
Acanthomine A and 5-bromo-8-methoxy-1-methyl-β-carboline.

Acanthomine A (**756**) was obtained from the marine sponge *Acanthostrongylophora ingens* (Figure 140) [324]. 5-Bromo-8-methoxy-1-methyl-β-carboline (**757**) was isolated from the New Zealand marine bryozoan *Pterocella vesiculosa* and displayed cytotoxicity against the P388 murine leukemia cell line and inhibitory activity against *Bacillus subtilis*, *Candida albicans* and *Trichophyton mentagrophytes* [325].

**Figure 141:**
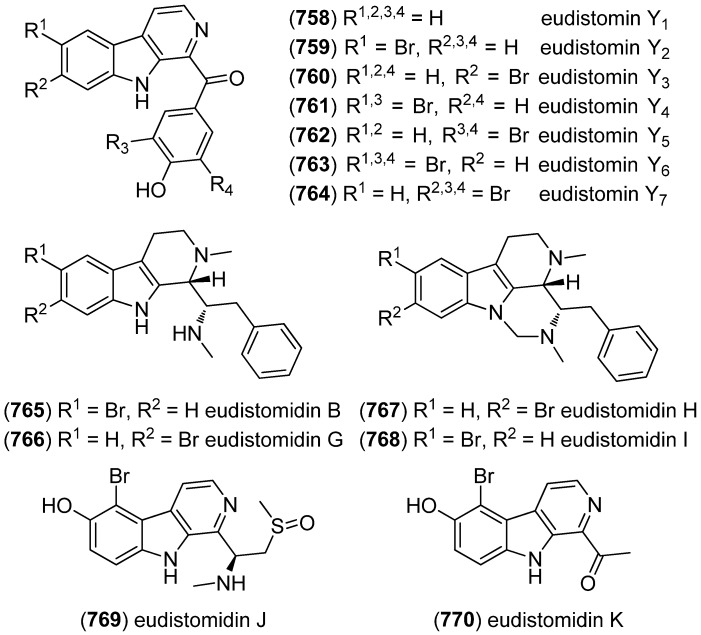
Eudistomins Y_1_–Y_7_ and eudistomidins B and G–K.

Eudistomins Y_1_–Y_7_ (**758**–**764**) were isolated from the marine tunicate *Eudistoma* sp. (Korea) and revealed antibacterial activity against *Staphylococcus epidermis* and *Bacillus subtilis* (Figure 141) [326]. Eudistomidins G–K (**766**–**770**) were obtained from the Okinawan tunicate *Eudistoma glaucus* and the structure of eudistomidin B (**765**) was revised. Eudistomidins G (**766**) and B (**765**) showed cytotoxic activity towards murine leukemia cells L1210 (IC_50_ 4.8 and 4.7 μg/mL, respectively), whereas eudistomidin J (**769**) was active against murine leukemia cells P388 (IC_50_, 0.043 μg/mL) and L1210 (IC_50_ 0.047 μg/mL) and human epidermoid carcinoma cells KB (IC_50_ 0.063 μg/mL) [327,328].

**Figure 142:**
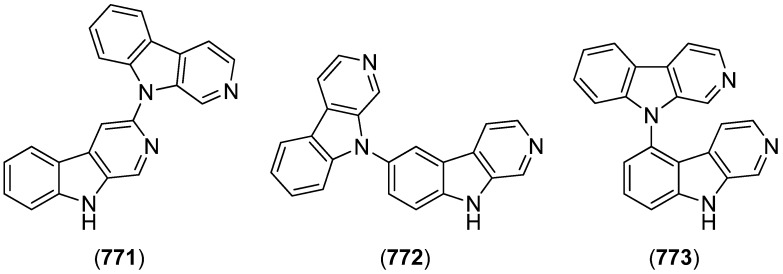
β-Carboline dimers.

β-Carboline dimers **771**–**773** were isolated from an ascidian *Didemnum* sp. (Figure 142) [329].

**Figure 143:**
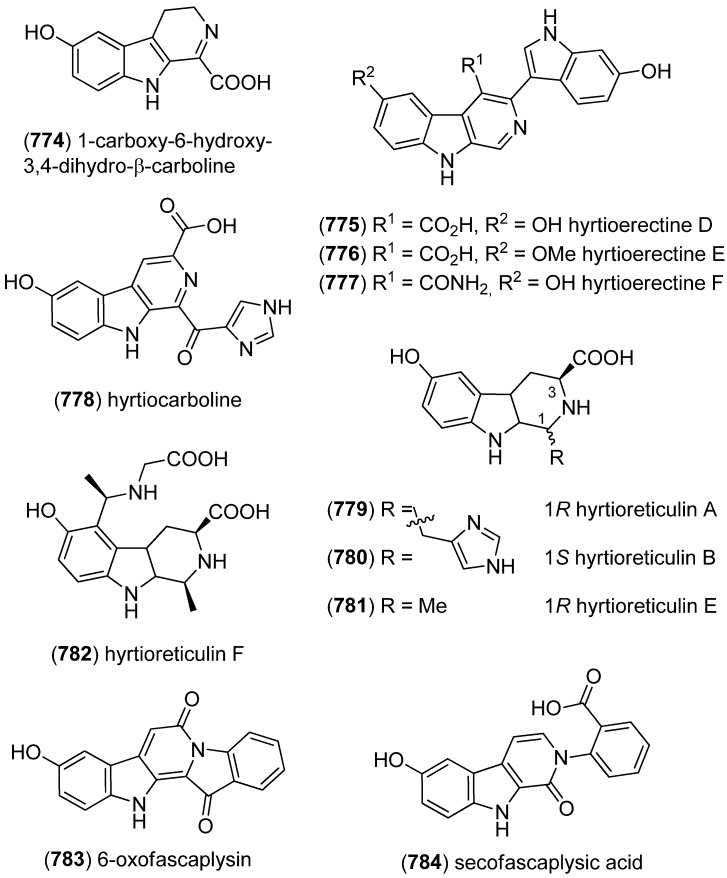
Carboline derivatives from *Hyrtios* species.

1-Carboxy-6-hydroxy-3,4-dihydro-β-carboline (**774**) [330] and hyrtioerectines D–F (**775**–**777**) [331] have been isolated from the marine sponge *Hyrtios* sp. (Figure 143). 1-Carboxy-6-hydroxy-3,4-dihydro-β-carboline (**774**) showed only weak activity against the isocitrate lyase (ICL) of *Candida albicans* (ATCC 10231) [330]. Hyrtioerectines D–F (**775**–**777**) displayed antimicrobial activity against *C. albicans* and *Staphylococcus aureus*, as well as free radical scavenging and anticancer (A549, HT-29, MDA-MB-231) activities. Diphenolic compounds (**775**) and (**777**) showed higher activities than (**776**) [331]. Hyrtiocarboline (**778**) was obtained from the marine sponge *Hyrtios reticulatus* (Papua New Guinea) and exhibited selective antiproliferative activity towards the tumor cell lines H522-T1, MDA-MB-435 and U937 [332]. Studies of the same organism led to the isolation of hyrtioreticulins A–F. Hyrtioreticulins A, B, E and F (**779**–**782**) possess a β-carboline framework while hyrtioreticulins C (**699**) and D (**700**) belong to the group of azepino-indole-type alkaloids (Figure 126). In addition, hyrtioreticulins A (**780**) and B (**781**) were found to inhibit ubiquitin-activating enzyme E1 [296,297]. 6-Oxofascaplysin (**783**) and secofascaplysic acid (**784**) were obtained from an Australian *Hyrtios* sp. Both exhibited low cytotoxic activity towards LNCaP and NFF cell lines [333].

**Figure 144:**
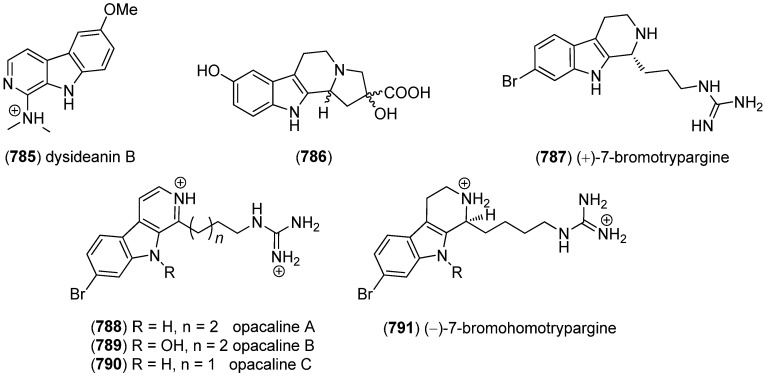
Carboline alkaloids from *Dysidea* sp., *Isis minorbrachyblasta*, *Ancorina* sp., and *Pseudodistoma opacum*.

Dysideanin B (**785**) was obtained from the marine sponge *Dysidea* sp. and showed antibacterial activity against *Bacillus subtilis*, *Staphylococcus aureus*, *Escherichia coli* and *Vibrio alginolyticus* (Figure 144) [334]. 2,3,5,6,11,11*b*-Hexahydro-2-hydroxy-1*H*-indolizino[8,7-*b*]indole-2-carboxylic acid (**786**) was isolated from extracts of the South China Sea gorgonian *Isis minorbrachyblasta* [335]. Examination of the Australian marine sponge *Ancorina* sp. led to discovery of (+)-7-bromotrypargine (**787**), which showed growth inhibitory effects towards two *Plasmodium falciparum* strains (Dd2 and 3D7) [336]. Opacalines A–C (**788**–**790**) and (−)-7-bromohomotrypargine (**791**) were obtained from the New Zealand ascidian *Pseudodistoma opacum.* Opacalines B (**789**) and C (**790**) showed antimalarial activity against a chloroquine-resistant strain of *Plasmodium falciparum* [337].

**Figure 145:**
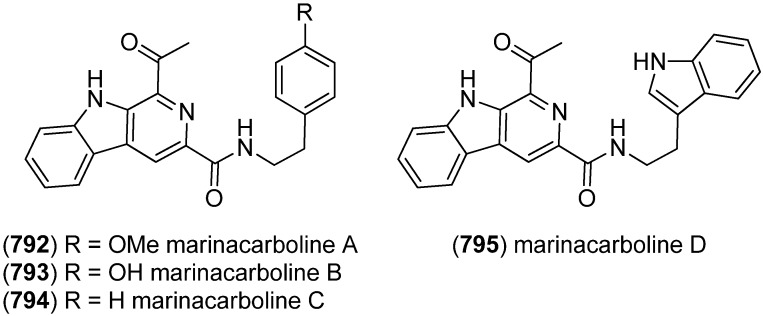
Marinacarbolines A–D.

Marinacarbolines A–D (**792**–**795**) together with prenylated 13-*N*-demethyl-methylpendolmycin (**749**) and methylpendolmycin-14-*O*-α-glucoside (**453**, see Figure 72) were isolated from the actinomycete *Marinactinospora thermotolerans* SCSIO 00652 (Figure 145). They did not exhibit cytotoxic activities, but showed antiplasmodial activities against *Plasmodium falciparum* strains 3D7 and Dd2 [195].

**Figure 146:**
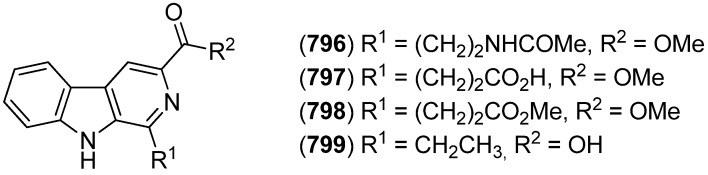
β-Carboline-3-carboxylates.

Examination of the actinomycete *Actinomadura* BCC 24717 led to the isolation of β-carbolines methyl 1-(2-methyl carbamate)ethyl-β-carboline-3-carboxylate (**796**), methyl 1-(propionic acid)-β-carboline-3-carboxylate (**797**), methyl 1-(methyl propionate)-β-carboline-3-carboxylate (**798**) and 1-ethyl-β-carboline-3-carboxylic acid (**799**) (Figure 146). Compound **799** showed cytotoxic activity towards Vero cells [91]. Compound **797** was also isolated from *Microbispora* sp. LGMB259, an endophytic actinomycete isolated from *Vochysia divergens.* It neither displayed antibacterial or antifungal activity, nor cytotoxicity against the human cancer cell lines PC3 and A549 [338].

**Figure 147:**
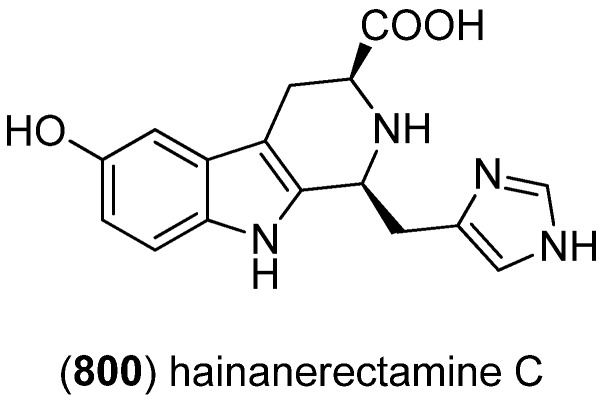
Hainanerectamine C.

Hainanerectamine C (**800**), from the Hainan marine sponge *Hyrtios erectus* displays inhibitory effects on serine/threonine kinase Aurora A, which is involved in cell division regulation, yet it had no cytotoxic effects on the tumor cell lines A549 and HT-29 (Figure 147) [98].

## 3. Conclusions

In the twelve year period since the last review on marine indole alkaloids, the numbers of known compounds of this class has increased dramatically. Intense research, mainly driven by teams from the Asian pacific region, has provided the scientific community with new representatives of formerly known structural families but also provided entirely new chemotypes. This gain of structural knowledge is likely to trigger activities in the areas of synthetic organic chemistry, pharmacology and medicinal chemistry as the indole skeleton is the basis of many important drugs and experimental compounds in the biomedical field.
